# Merging metabolomics and genomics provides a catalog of genetic factors that influence molecular phenotypes in pigs linking relevant metabolic pathways

**DOI:** 10.1186/s12711-025-00960-8

**Published:** 2025-03-06

**Authors:** Samuele Bovo, Anisa Ribani, Flaminia Fanelli, Giuliano Galimberti, Pier Luigi Martelli, Paolo Trevisi, Francesca Bertolini, Matteo Bolner, Rita Casadio, Stefania Dall’Olio, Maurizio Gallo, Diana Luise, Gianluca Mazzoni, Giuseppina Schiavo, Valeria Taurisano, Paolo Zambonelli, Paolo Bosi, Uberto Pagotto, Luca Fontanesi

**Affiliations:** 1 Animal and Food Genomics Group, Division of Animal Sciences, Department of Agricultural and Food Sciences, University of Bologna, Bologna, Italy; 2 Endocrinology Research Group, Center for Applied Biomedical Research, Department of Medical and Surgical Sciences, University of Bologna, Bologna, Italy; 3 Division of Endocrinology and Prevention and Care of Diabetes, IRCCS Azienda Ospedaliero-Universitaria di Bologna, Policlinico di Sant’Orsola, Bologna, Italy; 4 Department of Statistical Sciences “Paolo Fortunati”, University of Bologna, Bologna, Italy; 5 Biocomputing Group, Department of Pharmacology and Biotechnology, University of Bologna, Bologna, Italy; 6 Laboratory on Animal Nutrition and Feeding for Livestock Sustainability and Resilience, Division of Animal Sciences, Department of Agricultural and Food Sciences, University of Bologna, Bologna, Italy; 7 Associazione Nazionale Allevatori Suini, Rome, Italy

## Abstract

**Background:**

Metabolomics opens novel avenues to study the basic biological mechanisms underlying complex traits, starting from characterization of metabolites. Metabolites and their levels in a biofluid represent simple molecular phenotypes (metabotypes) that are direct products of enzyme activities and relate to all metabolic pathways, including catabolism and anabolism of nutrients. In this study, we demonstrated the utility of merging metabolomics and genomics in pigs to uncover a large list of genetic factors that influence mammalian metabolism.

**Results:**

We obtained targeted characterization of the plasma metabolome of more than 1300 pigs from two populations of Large White and Duroc pig breeds. The metabolomic profiles of these pigs were used to identify genetically influenced metabolites by estimating the heritability of the level of 188 metabolites. Then, combining breed-specific genome-wide association studies of single metabolites and their ratios and across breed meta-analyses, we identified a total of 97 metabolite quantitative trait loci (mQTL), associated with 126 metabolites. Using these results, we constructed a human-pig comparative catalog of genetic factors influencing the metabolomic profile. Whole genome resequencing data identified several putative causative mutations for these mQTL. Additionally, based on a major mQTL for kynurenine level, we designed a nutrigenetic study feeding piglets that carried different genotypes at the candidate gene kynurenine 3-monooxygenase (*KMO*) varying levels of tryptophan and demonstrated the effect of this genetic factor on the kynurenine pathway. Furthermore, we used metabolomic profiles of Large White and Duroc pigs to reconstruct metabolic pathways using Gaussian Graphical Models, which included perturbation of the identified mQTL.

**Conclusions:**

This study has provided the first catalog of genetic factors affecting molecular phenotypes that describe the pig blood metabolome, with links to important metabolic pathways, opening novel avenues to merge genetics and nutrition in this livestock species. The obtained results are relevant for basic and applied biology and to evaluate the pig as a biomedical model. Genetically influenced metabolites can be further exploited in nutrigenetic approaches in pigs. The described molecular phenotypes can be useful to dissect complex traits and design novel feeding, breeding and selection programs in pigs.

**Supplementary Information:**

The online version contains supplementary material available at 10.1186/s12711-025-00960-8.

## Background

The pig (*Sus scrofa*) is one of the most economically important livestock species, serving as a primary supplier of meat for human consumption. Additionally, it is considered one of the most important non-rodent biomedical models due to its physiological similarities to humans [1]. Specifically, as the pig is a single-stomached omnivore with gut physiology and metabolism comparable to humans, it is a valuable model for studying nutrition and metabolic disorders that are relevant to humans.

Metabolomics is the study of the plethora of metabolites, which are small biological molecules that act as intermediates or end products of chemical reactions in an organism [2]. Metabolites represent molecular phenotypes (also indicated as metabotypes) that are the direct products of the activities of enzymes pertaining to metabolic pathways, including catabolism and anabolism of nutrients. The level of a metabolite in a biofluid, its uptake, its transfer and its regulatory mechanisms are therefore components of a metabotype. Therefore, metabotypes are considered simple and “internal” phenotypes that can be used to dissect more complex “external” phenotypes or end phenotypes [3] that have relevant and direct economic values. In pigs, production performances (e.g., growth rate, fat deposition, lean meat deposition, feed conversion rate) are the end phenotypes [4]. Metabolic profiles are quite stable, meaning that their baseline levels are only partially affected by environmental perturbations. As a result, the heritability of many metabotypes can be quite high, providing opportunities to uncover the genetic factors responsible for their variation across individuals in a population [5].

In humans, the combination of metabolomics for blood-derived biofluids and genome-wide association studies (GWAS) has already proven successful in identifying genetic factors that influence metabotypes, also known as genetically influenced metabolites (GIM) [6]. This information can be used to decipher the genetic mechanisms that affect metabolism and to better describe other complex physiological conditions and diseases [7–10]. Since metabolites offer molecular phenotypes that are close to an individual’s genotype profile, a relatively smaller number of individuals (typically a few hundred) is required to obtain meaningful results than would be needed in a GWAS for more complex phenotypes [11]. Genetically influenced metabolites identified in humans have been shown to generally have greater effect sizes than most other complex traits and diseases and can be explained by loci that are often located in or near genes that encode enzymes, metabolite transporters, and regulators of metabolism [7, 8, 11].

A few studies have reported preliminary characterizations of the livestock metabolomes by investigating different biofluids for various purposes [4, 12]. These studies focused primarily on specific questions about the physiological and health status of the animals and the effects of feeding on their metabolic patterns, leading to the identification of some biomarkers that are useful for monitoring and diagnostic purposes (e.g., [13–17]). Other studies have investigated how genetic factors can influence animal metabolomes. Initial studies focused on breed differences in metabolomic profiles and later studies defined how genomic variants are associated with the levels of certain metabolites in plasma, serum, milk, and in various tissues [18–24].

In this study, we demonstrated the utility of merging metabolomics and genomics in pigs to uncover some of the genetic factors that influence mammalian metabolism. We obtained a targeted characterization of the plasma metabolome for two different Western pig breeds, Large White and Duroc, using sib-tested animals, reared in standard environments. We then used the metabolomic profiles of these pigs to identify GIM (i) by estimating the heritability of metabolite levels, (ii) identifying genomic regions associated with metabolite levels (defined as metabolite Quantitative Trait Loci or mQTL) through GWAS, (iii) performing systems biology analyses of metabolic pathways, and (iv) constructing a human-pig comparative catalog of genetic factors influencing the metabolomic profile. Additionally, we designed a nutrigenetic study based on a major mQTL to further validate its effects. Considering that many metabolites are relevant in animal nutrition, this study may open important opportunities for using GIM to integrate genetic information into pig nutrition.

## Methods

### Large White and Duroc pigs, blood collection and liver samples

A total of 920 Large White pigs (303 castrated males and 617 gilts, obtained from 86 boars and 358 litters) and 389 Duroc pigs (120 castrated males and 269 gilts, obtained from 66 boars and 189 litters) were sampled across 26 different slaughtering days. The animals were from triplets of siblings from the same litter, consisting of 2 females and 1 castrated male. These pigs were individually performance tested at the Central Station of the National Pig Breeder Association (ANAS) for genetic evaluation of a boar from the same litter (sib-testing). Pigs started their performance evaluation at 30–45 days of age and continued until they reached a live weight of 155 ± 5 kg [25]. All animals were fed the same standard commercial feed for fattening pigs, following the production rules of the Parma and San Daniele dry-cured ham consortia. At the end of the evaluation, the animals were transported to the same commercial abattoir, where they were slaughtered in the morning (07.00–08.00 a.m.), following standard procedures, including a 12-h overnight fasting period and the use of electrical stunning.

For each pig, 2 aliquots of blood were collected at the abattoir just after jugulation, directly from the draining carotid artery into a tube with ethylenediaminetetraacetic acid (EDTA) to prevent coagulation for the preparation of plasma (Vacutest Kima, Padua, Italy). After collection, blood samples were refrigerated on ice for 2 h. One aliquot was then stored at − 20 °C for subsequent DNA extraction. The other aliquot was used for the preparation of plasma after centrifugation at 3000 rpm for 10 min at + 4 °C. The plasma was then divided in several additional sub-aliquots that were stored at − 80 °C for metabolomic analysis. At the abattoir, liver samples were collected from the same pigs and immediately frozen in liquid nitrogen. These samples were then stored at − 80 °C.

### Whole genome genotyping and data filtering

DNA was extracted from blood samples stored at − 20 °C using the Wizard Genomic DNA Purification kit (Promega Corporation, Madison, WI, USA). All Large White and Duroc pigs were then genotyped with the Illumina PorcineSNP60 BeadChip v.2 (Illumina Inc., San Diego, CA, USA), which analyzes 61,565 single nucleotide polymorphisms (SNPs). This SNP panel was also used to genotype the piglets included in the nutrigenetic longitudinal study (see below). Genotyping data were filtered using PLINK v.1.9, discarding animals with a call rate < 0.9 and SNPs with a call rate < 0.95, minor allele frequency (MAF) < 0.05, and Hardy–Weinberg equilibrium *P* < 0.001, and SNPs that are located on sex chromosomes or not uniquely mapped on the Sscrofa11.1 genome version [26].

### Targeted metabolomics and data cleaning

Metabolomics measurements of plasma metabolites were carried out using the Biocrates AbsoluteIDQ p180 Kit (Biocrates Life Science AG, Innsbruck, Austria), which allows for the quantification of a panel of 186 metabolites (or 188 metabolites, based on a subsequent upgrade), including 21 amino acids, 19 (or n. 21) biogenic amines, 1 hexoses’ pool, 40 acylcarnitines, 15 sphingomyelins, 76 phosphatidylcholines, and 14 lysophosphatidylcholines. The list of all metabolites with the full biochemical name and abbreviation is in Additional file 1: Table S1. The analytical platform consisted of a Serie 200 HPLC system (PerkinElmer, Waltham, Massachusetts, USA) coupled with an API 4000 QTrap mass spectrometer (AB-Sciex, Foster City, CA, USA). Plate preparation followed the manufacturer’s instructions (Biocrates Life Sciences AG). In house quality controls, obtained by pooling equal volumes of plasma from 10 randomly chosen and unrelated pigs, were included in each of the 19 analyzed plates. The analytical process was carried out using the MetIQ software package, which is an integral part of the AbsoluteIDQ p180 Kit (Biocrates Life Science AG). Concentrations of the analyzed metabolites were reported in μM units.

Data quality control was carried out as previously described [27]. In summary: (i) metabolites with all missing values (NA) or all zero values in at least one plasma pool were excluded; (ii) samples were identified as outliers if measured concentration for the sample deviated 1.5 times the interquartile range below or above the corresponding median for > 30% of the analyzed metabolites; (iii) animals with a missing value for at least one of the analyzed metabolites were eliminated; and (iv) metabolites with an inter-plate coefficient of variation < 30%, as estimated by the in-house quality controls, were removed. Subsequently, for each metabolite, zero values were imputed using random values generated from a uniform distribution ranging from zero to the minimum non-zero measured concentration.

The metabolomics and genomics data were merged, resulting in a final dataset for Large White pigs that included 787 animals (256 castrated males and 531 gilts) × 169 metabolites × 45,423 SNPs, whereas for Duroc pigs the dataset included 286 animals (87 castrated males and 199 gilts) × 164 metabolites × and 38,631 SNPs. Ratios between metabolite concentrations were also calculated, resulting in 14,196 and 13,366 ratios for Large White and Duroc breeds, respectively.

Data were processed in the R v.4.2.2 [28].

### Metabolomics data processing

For GWAS, the Large White and Duroc datasets were processed separately, using the R v.4.2.2 using the function “boxcox” of the “MASS” package [28]. Briefly, data were normalized using a Box-Cox transformation (in a regression model) and then cleaned using linear regression models to remove the effects of systematic environmental and technical factors, as previously described [27]. For Box-Cox transformation, selection of the λ parameter followed a grid search (3001 tested values in the range [− 3, + 3]) using maximum likelihood for a regression model that included sex, carcass weight, and blood collection date. To remove the effects of systematic environmental and technical factors, Box-Cox transformed data were regressed on covariates (fixed effects: animal sex, animal carcass weight, and blood collection date) and residuals were obtained for GWAS, using the following model:

1 $${y}_{i}= {\beta }_{0}+{\beta }_{w}{w}_{i}+{\beta }_{s}{s}_{i}+\sum_{j=1}^{J-1}{\beta }_{Cj}{d}_{ij}+{\varepsilon }_{i},$$

where *y*_*i*_ is the level of the metabolite for the *i*th animal, *β*_*0*_ is the intercept term, *w*_*i*_ indicates the carcass weight of the *i*th animal, *s*_*i*_ is a dummy variable representing the sex of the *i*th animal, *d*_*i1*_*,…,d*_*i(J-1)*_ is a set of *J* = 26 dummy variables coding the blood collection date for the *i*th animal, while *β*_*w*_, *β*_*s*_ and *β*_*C*j_ are the corresponding regression coefficients, and ε_i_ is the residual. Metabolite levels adjusted for confounding effects were obtained by estimating the residuals as:

2 $${{e}_{i}=y}_{i}-{\widehat{y}}_{i},$$

with:

3 $${\widehat{y}}_{i}={b}_{0}+{b}_{w}{w}_{i}+{b}_{s}{s}_{i}+\sum_{j=1}^{J-1}{b}_{Cj}{d}_{ij},$$

where *b*_*0*_, *b*_*w*_, *b*_*s*,_ *b*_*Cj*_ (*j* = *1,…, J−1*) are the least squares estimates of model parameters.

For network generation, data were processed as described above but using a common λ value to normalize the entire metabolic profile. This common λ value was that which was shared by most metabolites based on the set of λ values within the 95% confidence interval of each tested λ value. Then, the transformed data were regressed on covariates as described above (Eqs. (1)–(3)) and residuals were obtained.

Data were processed both separately for each breed and combined across breeds. For the combined dataset, breed was added as a covariate in Eq. (1).

### GWAS, meta-GWAS and heritability estimation

We carried out breed specific GWAS and then subjected the results to meta-analysis. Association studies were based on the additive genetic model. Using GEMMA v.0.94.1, we implemented a univariate linear mixed effect model that accounted for population stratification through the generation and inclusion of a centered genomic relationship matrix (**K**) [29]. The following linear mixed effect model was adopted:

4 $$\mathbf{y}=\mathbf{W}{\varvec{\upalpha}}+\mathbf{x}\beta +\mathbf{g}+\mathbf{e},$$

where ***y*** (*n* × 1) is a vector containing the metabolite level for the *n* animals (residuals of the normalized metabolite level; Eq. (1) to Eq. (3)), **W** (*n* × *k*) is a covariate matrix with *k* = 1 (a column of 1 s) and **α** is the *k*-dimensional vector of covariate effects, **x** (*n* × 1) is the vector containing genotypes for the *i*th SNP (coded as 0, 1, 2, according to the number of copies of the minor allele), *β* is the additive fixed effect of the *i*th SNP on the metabolite levels, **g** ~ *N*(**0**, σ^2^_g_ **K**) is a multivariate Gaussian polygenic effect, with covariance matrix proportional to the relationship matrix **K** (*n* × *n*), and **e** ~ *N*(**0**, σ^2^_e_ **I**) is a multivariate Gaussian vector of uncorrelated residuals. The assessment of the association between each SNP and the metabolite level was obtained by testing the null hypothesis H_0_: β = 0 using the Wald test. GWAS results were processed in the R v.4.2.2 environment to generate Manhattan plots [28]. The percentage of variance explained (PVE) by a given SNP was calculated as described in Shin et al. [30]. Briefly, PVE was estimated as follows:

5 $$\text{PVE}= \frac{2{\widehat{\beta }}^{2}\times \text{MAF}\times (1-\text{MAF})}{2{\widehat{\beta }}^{2}\times \text{MAF}\times \left(1-\text{MAF}\right)+{(\text{se}(\widehat{\beta }))}^{2}\times 2\text{N}\times \text{MAF}\times (1-\text{MAF})},$$

where: $\widehat{\beta }$, se($\widehat{\beta }$), and MAF are, respectively, the effect size estimate, the standard error of the effect size estimate, and the minor allele frequency of a given SNP. N represents the sample size.

Significant associations in single metabolite GWAS were detected by adopting a linkage disequilibrium (LD)-based Bonferroni correction to identify suggestive associations (assuming a level of α = 0.05 and a number of independent tests equal to the number of SNPs with an LD *r*^*2*^ < 0.25 [31]). Determination of LD was carried out as described in Monir and Zhu [31] using PLINK v.1.9 [26]. This analysis returned 23,076 and 13,269 independent SNPs for Large White and Duroc pigs, respectively. Significance of metabolite ratios for GWAS considered the p-gain statistics, estimating the critical level for the p-gain (threshold) as 10 times the number of tested ratios (assuming a level of α = 0.05) [32].

GWAS results were then combined in a meta-analysis (meta-GWAS) using the weighted Z-score model implemented in Metal software (release 2011) [33]. The model considered the direction of effect (β) and P-value of the association of a given SNP that was obtained for each breed by combining them with weights based on the sample sizes. The Bonferroni corrected hypothesis test for association with a given metabolite accounted for the 31,007 SNPs that were shared and tested in both breeds, whereas the LD-based Bonferroni correction accounted for 20,200 independent SNPs. The critical level for p-gain in meta-GWAS of metabolite ratios was set at 10 times the number of tested ratios (assuming a level of α = 0.05).

Narrow sense heritability, based on pedigree records of a total of four generations (h^2^_P_), was estimated using the R package “gap” v.1.5-3. For this estimation, we used Eq. (4) including a kinship matrix. Genomic heritability (h^2^_SNP_) was estimated from the univariate linear mixed effect model (Eq. (4)) including a genomic relationship matrix, as implemented in GEMMA v.0.94.1 [29].

### Annotation of GWAS results

Distinct mQTL regions on the same chromosomes were declared when significant SNPs (identified as described above, combining the different approaches) were separated by non-significant SNPs for a region of at least 1 Megabase (Mb) in the same breed(s) in which the mQTL were identified (considering the high level of LD in these pig populations) [34, 35]. When an mQTL was identified in different breeds and for different metabolites, we considered a distance of 0.5 Mb. The most significant SNP-metabolite pair (or ratio, considering the p-gain) was then used to identify the corresponding mQTL.

GWAS results were annotated by retrieving the set of annotated protein coding genes located in the ± 500-kb flanking regions of the significant SNP from the Sscrofa11.1 National Center for Biotechnology Information’s (NCBI) GFF file. Functional relevance of genes was evaluated by detailed analysis of the scientific literature, the Gene Cards database, and known metabolite-gene associations retrieved from the GWAS Catalog, PhenoScanner V2, KEGG, HMDB, and PubChem Chemical Co-occurrences in Literature database [36–41].

### Whole genome resequencing, variant calling and linkage disequilibrium analyses

A total of 88 Large White, 35 Duroc and 35 Landrace pigs underwent whole genome resequencing at ~ 20 ×, with individual DNA extracted using the protocol described above. The Large White and Duroc pigs were a subset of the performance tested pigs described above, chosen including pigs from different litters. The Landrace pigs were other performance tested animals provided by ANAS (these pigs were not used for metabolomic analyses). The inclusion of Landrace pigs was specifically for comparative analyses with the other two breeds for the chromosome region that includes a major mQTL, as described later. Genomic DNA was extracted and purified as described by Bovo et al. [25] Sequencing libraries were produced (150-bp paired ends; 400-bp insert size) and sequenced on a BGISeq500 machine. Reads were mapped on the Sscrofa11.1 reference genome using the BWA v.0.7.17 and then deduplicated with Picard v.2.1.1 (https://broadinstitute.github.io/picard/) [42]. Variant calling and filtering were performed using GATK4 haplotypecaller and variantfiltration (hard-filter; basic filtering thresholds for SNP and insertions/deletions or indel, as recommended in the manual), respectively [43]. Only bi-allelic variants located within the ± 500-kb flanking regions of each significantly associated SNP were retained for further analyses. Allele frequencies were then estimated for each population. The Variant Effect Predictor (VEP) tool was utilized to map gene positions and predict the effect of each variant (in conjunction with the SIFT tool for assessing potential deleterious effects of missense variants on translated proteins) [44]. Subsequently, LD analyses were carried out between the SNPs identified in the GWAS (mQTL) and the variants found in the candidate genes. These analyses were carried out separately for each breed using PLINK v.1.9 [26].

### Identification and analyses of variants in the kynurenine 3-monooxygenase (*KMO*) gene for a major mQTL

Haplotype information for the *KMO* gene was reconstructed using whole genome resequencing data obtained from the whole genome sequence of the Large White, Duroc, and Landrace pigs described above. Two major haplotypes were identified and named based on the two alleles of the lead SNP (rs81278711-A and rs81278711-G) associated with the level of plasma kynurenine, as determined in the GWAS. The whole genome resequence data was also used to obtain allele frequencies in the same breeds.

Whole genome sequencing datasets produced from 20 different DNA pools were obtained from Bovo et al. [45], each containing DNA of 30–35 pigs, representing 19 different European local pig breeds (Alentejana and Bísara from Portugal; Majorcan Black from Spain; Basque and Gascon from France; Apulo-Calabrese, Casertana, Cinta Senese, Mora Romagnola, Nero Siciliano and Sarda from Italy; Krškopolje pig from Slovenia; Black Slavonian and Turopolje from Croatia; Moravka and Swallow-Bellied Mangalitsa from Serbia; Schwäbisch-Hällisches Schwein from Germany; Lithuanian indigenous wattle and Lithuanian White old type from Lithuania), as well as a European wild pig population. Polymorphisms in the *KMO* gene region were identified and annotated as described previously. Allele frequencies at the polymorphic sites were estimated by counting the number of reads that cover the variant positions.

Using *KMO* missense polymorphisms identified from whole genome sequence of the Large White, Duroc, and Landrace pigs and of the DNA pools, we evaluated single amino acid polymorphisms in relation to both (i) the KMO protein sequence through an analysis based on InterPro (ii) and the KMO protein structure, based on the model retrieved from the SwissModel repository [46, 47].

Genotyping data for the *KMO* exon 17 indel (that adds/eliminates one amino acid; g.12489135_12489136insACC) in the piglets included in the longitudinal nutrigenetic study (see below) was obtained by Sanger sequencing of a 414 bp amplicon produced by PCR amplification using primers designed on exon 17 of the same gene (forward: 5ʹ-CAGGACTTCAGCTAGTGGTCA-3ʹ; reverse: 5ʹ-ATTTTGATCCTGTTTTGGTCAC-3ʹ). PCR was performed on an Applied Biosystem SimpliAmp Thermal Cycler (Thermo Fisher Scientific Inc., Waltham, MA, USA) in a total reaction volume of 14 µL including: 2 × Kapa Hifi HotStart ReadyMix PCR kit (Kapa Biosystems, Boston, MA, USA); 20–50 ng of template DNA; 10 pmol of each primer. PCR profile was as follows: an initial denaturation step at 95 °C for 5 min; 35 cycles of alternate temperatures (30 s at 95 °C, 30 s at 60 °C; 30 s at 72 °C) followed by a final extension step at 72 °C for 5 min.

### Pigs included in the longitudinal nutrigenetic study and analysis of kynurenine pathway metabolites

A total of 16 weaned Large White × Landrace crossbred piglets were included in the nutrigenetic experiment. Genotyping data for these piglets derived from the Illumina PorcineSNP60 BeadChip v.2 (Illumina Inc.; see above). Eight of these piglets were homozygous for the rs81278711-A allele and the exon 17 deletion, while the other 8 were homozygous for the rs81278711-G allele and for the exon 17 insertion. These piglets came from 4 different litters, with the alternative genotypes evenly distributed within each litter (2 + 2). The piglets were weaned at 28 days of age (day 0 of the trial, with average bodyweight = 6.825 ± 1.690 kg) and then penned in individual cages. Throughout the experiment, unless otherwise specified, all piglets were fed a standard post-weaning diet, ad libitum. The diet was formulated to be slightly deficient compared to the NRC 2012 feeding requirements, but adequate for the European standards, and without antimicrobial additives or pharmaceuticals [48]. More specifically, the diet was formulated to contain a 16.5% standard ileal digestible Trp to Lys ratio, a value marginally low, kept to emphasizing the potential effects of *KMO* genotypes and the subsequent addition of Trp to the diet. Information on the diet and related amino acid content is available in Additional file 2: Tables S2, S3. On day 7, all piglets (with an average bodyweight of 7.04 ± 1.71 kg) had their first blood sample taken after the morning meal. They were then fed the next meal, which contained a quantity of Trp equal to twice the required amount by doubling the supplementation compared to the basic diet. After 3 h, a second blood sample was collected. All blood samples were obtained through venipuncture in the vena cava, collected in EDTA tubes, which were then centrifuged at 3000×*g* for 10 min at 4 °C. Plasma samples were aliquoted and then stored at − 80 °C for subsequent targeted metabolomic analyses, using an LC–MS/MS platform at Bevital SA (Bergen, Norway) to quantify several key metabolites of the kynurenine pathway [see Additional file 3: Figure S1], as previously described: tryptophan (Trp), kynurenine (KYN), 3-hydroxykynurenine (HK), kynurenic acid (KA), xanthurenic acid (XA), anthranilic acid (AA), 3-hydroxyanthranilic acid (HAA), and quinolinic acid (QUIN) [49]. The piglets were sacrificed on day 10 with an intracardiac injection of Tanax^®^ (0.5 mL/kg bodyweight) after being anesthetized with Zoletil 100 (15 mg/kg bodyweight). Liver samples were then collected, immediately frozen in liquid nitrogen and stored at − 80 °C for subsequent analyses.

### Mathematical modelling of the kynurenine pathway (KP)

Levels of KP metabolites between the two *KMO* genotypes (as derived from the nutrigenetic experimental design) were compared using the Wilcoxon Rank Sum Test. Results with *P* < 0.05 were considered statistically significant [see Additional file 2: Tables S4]. The KP metabolite levels were further analyzed using a kinetic modeling approach based on a set of ordinary differential equations (ODE). The model took all reactions mediated by enzymes endowed with a Michaelis–Menten kinetics into account and all the enzymes operating in their first-order regime. In this model, each reaction was described with a reaction rate (velocity) defined as:

6 $$v= \frac{{v}_{max}\cdot [\text{S}]}{{K}_{M}+[S]}\approx k\cdot \left[S\right],$$

provided that $\left[S\right]\ll {K}_{M}$, where *K*_*M*_ indicates the Michealis-Menten constant (affinity for the substrate), *v*_*max*_ is the maximal velocity and [S] is the substrate concentration. Considering the dependence from the enzyme levels, this equation can be rewritten as:

7 $$v= \frac{{k}_{cat} }{{K}_{M}}\cdot \left[S\right]\cdot \left[E\right],$$

where *k*_*cat*_ identifies the turnover number and [E] is the total amount of enzyme. Based on the reactions scheme shown in Additional file 3: Figure S1, we set up a system of ODE [see Additional file 2: Table S5] that describes the levels of KP metabolites at the steady states [see Additional file 2: Table S6]. The 3-hydroxykynurenine (HK) levels did not present any difference in concentration between the 2 *KMO* genotypes (*P* > 0.05), allowing us to assume the following equivalence:

8 $$[{HK}_{\text{rs}81278711-\text{GG}}^{ss}]={[HK}_{\text{rs}81278711-\text{AA}}^{ss}].$$

Considering the study of metabolites at the steady state, we can express the dependence of HK concentrations as a function of Trp concentrations as:

9 $$\left[{HK}^{ss}\right]=\frac{{k}_{\text{KMO}} \cdot \left[\text{KYN}\right]}{{k}_{\text{KYNU}2}} = \frac{{k}_{\text{TDO}/\text{IDO}} }{{k}_{\text{KYNU}2}+{k}_{\text{KAT}2}} \cdot \frac{{k}_{\text{KMO}}}{{k}_{\text{KYNU}1}+ {k}_{\text{KMO}}+{k}_{\text{KAT}1}} \cdot \left[\text{Trp}\right],$$

where each *k* represents a kinetic constant specific for each enzyme entering the KP route. Under the hypothesis that only *k*_KMO_ is different in the 2 pig groups, the Eq. (8) and Eq. (9) can be combined, and the equivalence can be simplified and expressed as:

10 $$\frac{{k}_{KMO}^{\text{rs}81278711-\text{GG}}}{({k}_{KYNU1}+{k}_{KAT1}+{k}_{KMO}^{\text{rs}81278711-\text{GG}})}=\frac{{k}_{KMO}^{\text{rs}81278711-\text{AA}}}{({k}_{KYNU1}+{k}_{KAT1}+{k}_{KMO}^{\text{rs}81278711-\text{AA}})}.$$

This implies that equal levels of 3-hydroxykynurenine are maintained only if ${k}_{KMO}\gg {k}_{KYNU1}+{k}_{KAT1}$ (and considering equal enzyme concentrations). As these constants are not available for porcine enzymes, we relied on values derived in humans by Stavrum et al. [50] [see Additional file 2: Table S7] that confirmed this inequality (*k*_KMO_ = 22 s^−1^ mM^−1^, *k*_KYNU1_ 0.46 s^−1^ mM^−1^ and *k*_KAT1_ = 2.08 s^−1^ mM^−1^). Considering that ${k}_{KMO}= \frac{{k}_{cat} }{{K}_{M}}$, changes in ${v}_{KMO}$ can be attributed to changes in affinity for the substrate (*K*_M_), or to the turnover number (*k*_cat_), or to the total amount of enzyme ([E]).

### Quantitative real time PCR (qPCR) and Western blotting analyses of KMO

Total RNA was extracted from liver samples of the 16 piglets in the longitudinal nutrigenetic study (8 piglets homozygous for the rs81278711-A allele and 8 homozygous for the rs81278711-G allele), as well as from an additional 8 Large White pigs that were used for metabolomic analyses with the Biocrates AbsoluteIDQ p180 Kit (Biocrates Life Science AG; see above): 4 gilts for each of the two homozygous genotypes that were all slaughtered on the same day (description of these pigs and their diet is reported above). The RNA was reverse transcribed and used for qPCR of *KMO*, with beta-2-microglobulin (*B2M*) serving as the housekeeping gene. qPCR reactions were performed in triplicate for each sample using the Kapa SYBR Fast qPCR Master Mix kit (Kapa Biosystems, Roche, Basel, Switzerland) on a QuantStudio 7 instrument (Thermo Fisher Scientific, Waltham, MA, USA). Average Cts were calculated for the pigs with the two *KMO* genotypes and the relative gene expression was calculated as 2^−∆∆Ct^ for each experimental design.

Proteins were extracted from the liver tissues of the same pigs, with 3 technical replicates for each sample. A total of 20 µg of proteins was separated by Sodium Dodecyl Sulphate—PolyAcrylamide Gel Electrophoresis (SDS-PAGE) using 12% polyacrylamide resolving gels with a 4% stacking gel (Gibco BRL/Thermo Fisher Scientific) for each sample. The proteins were electrophoretically transferred to 0.45 µm PVDF membranes and then incubated overnight at 2–8 °C with the primary Anti-KMO antibodies (Abcam, UK, ab130959) at a concentration of 2 µg/mL. After washing, the membranes were incubated with the secondary antibody. Subsequently, membranes were scanned, and the average band density was normalized to the average band density. Additional details on qPCR, Western blotting analyses and the results obtained are provided in Additional file 4: Text S1.

### Correlation networks and Gaussian graphical models

Metabolomic data from the Large White and Duroc pigs were used to assess the dependence between metabolite concentrations through Gaussian Graphical Models (GGM), which are undirect probabilistic graphical networks that estimate the conditional dependence structure among variables [12, 49]. GGM are based on partial correlation coefficients (PCC), which are pairwise Pearson’s correlation coefficients (r) corrected for all remaining variables. For comparison of the results, we calculated both simple Pearson’s correlation coefficients and full-order partial correlations by a matrix inversion operation. GGM were constructed both for each breed separately and for the combined breeds. Because Large White and Duroc had different numbers of animals evaluated (787 and 286, respectively), potentially affecting the estimation of PCC and the related statistical significance, 2 metabolites were considered linked if they had a PCC > 0.3 [7]. Additionally, a GGM was constructed separately for each breed, incorporating the genetic effects of the specific mQTL that were identified in the meta-GWAS for that breed. Briefly, metabolites were also regressed against the identified SNPs (coded as dummy variables) and then simple Pearson’s correlations and PCC were calculated. PCC were computed with the package R package ppcor v1.1. The networks were visualized using Cytoscape v.3.0.1 [51].

## Results

### Metabolites have a broad range of heritability in pigs

**Fig. 1:**
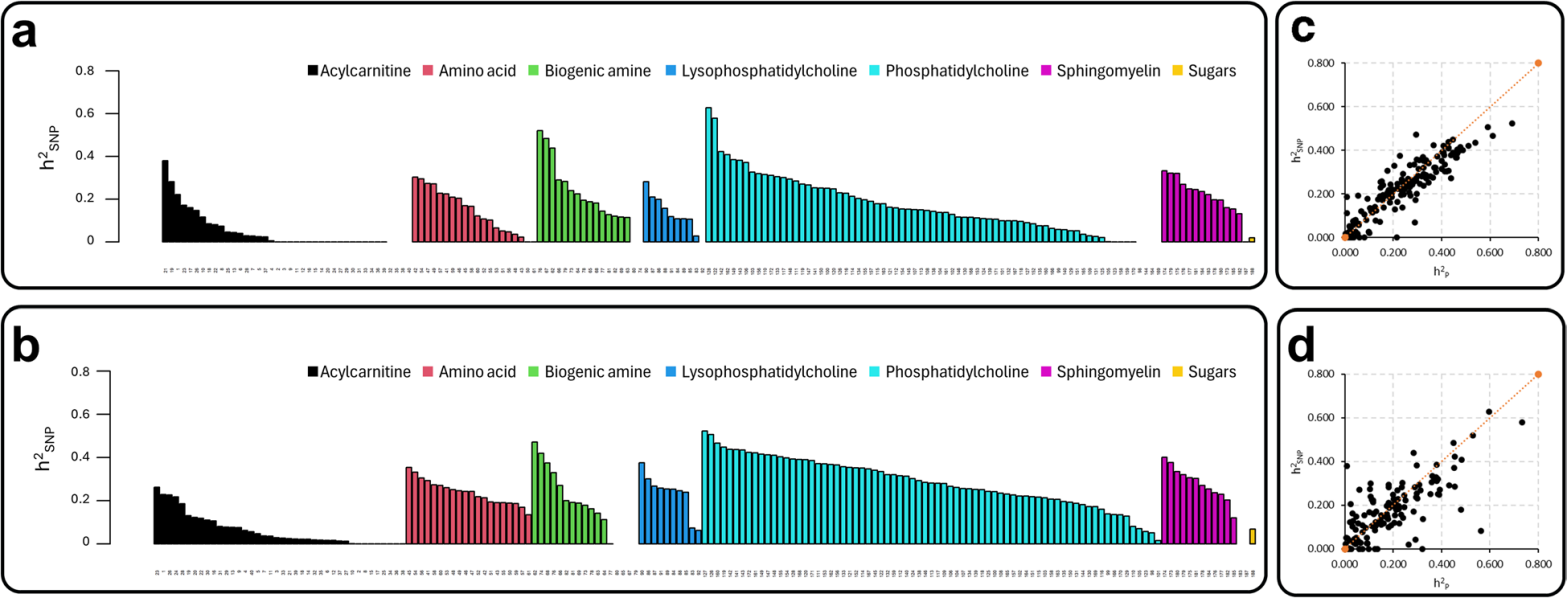
Heritability estimate profiles of different groups of metabolites in the two breeds. **a** Genomic heritability (h^2^_SNP_) in Large White pigs. **b** Genomic heritability (h^2^_SNP_) in Duroc pigs. **c**, **d** Scatter plots correlating narrow sense heritability (h^2^_P_) and genomic heritability (h^2^_SNP_) in Large White and Duroc pigs, respectively (Pearson’s correlation between the two measures of heritability were 0.90 in Large White and 0.76 in Duroc). All details, including information on h^2^_P_, are reported in Additional file 1: Table S8

We produced targeted plasma metabolomic profiles, including metabolites from six analyte classes [acylcarnitines, AC; amino acids, AAc; biogenic amines, BA; hexoses (including glucose), HE; glycerophospholipids (including phosphatidylcholines, PC; and lysophosphatidylcholines, lysoPC); and sphingomyelins, SM; see Additional file 1: Table S1], in Large White and Duroc breeds. We estimated the narrow sense heritability (h^2^_P_) of these metabolites using mixed linear models with pedigree data and then compared this information with the genomic heritability (h^2^_SNP_), estimated using genotyping data obtained from a 60 k SNP panel (Fig. 1a–d; see Additional file 2: Table S8). The heritability estimates averaged by metabolite class is reported in Table 1. Heritabilities estimated using the two approaches were similar, with average values across breeds of 0.22 ± 0.15 (h^2^_P_) and 0.19 ± 0.13 (h^2^_SNP_). The highest heritability estimates were observed in Large White pigs for phosphatidylcholine PC aa C40:5 (h^2^_P_ = 0.69 ± 0.10, h^2^_SNP_ = 0.52 ± 0.06) and in Duroc pigs for PC aa C38:6 (h^2^_P_ = 0.73 ± 0.16, h^2^_SNP_ = 0.58 ± 0.12) and PC aa C40:6 (h^2^_P_ = 0.60 ± 0.16, h^2^_SNP_ = 0.63 ± 0.11). When heritability estimates were related to the number of carbon atoms and double bounds of 3 analyzed metabolite classes (acylcarnitines, glycerophospholipids and sphingomyelins), some correlations emerged [see Additional file 3: Figures S2 and S3]. Other details on the relationship between the metabolite chemical structure and their heritability estimates are reported in Additional file 4: Text S2.

**Table 1:** Estimates of pedigree-based heritabilities (h^2^_P_) and of genomic heritabilities (h^2^_SNP_) of different metabolite classes in the Large White and Duroc breeds

Metabolite classes	Large White		Duroc	
	h^2^_P_	h^2^_SNP_	h^2^_P_	h^2^_SNP_
Amino acids	0.241 ± 0.079	0.238 ± 0.055	0.097 ± 0.095	0.145 ± 0.100
Essential amino acids^a^	0.221 ± 0.078	0.211 ± 0.041	0.067 ± 0.080	0.108 ± 0.088
Nonessential amino acids^b^	0.249 ± 0.078	0.242 ± 0.042	0.097 ± 0.091	0.162 ± 0.102
Acylcarnitines	0.054 ± 0.066	0.063 ± 0.073	0.080 ± 0.118	0.056 ± 0.089
Biogenic amines	0.192 ± 0.108	0.234 ± 0.128	0.210 ± 0.151	0.245 ± 0.131
Glycerophospholipids	0.300 ± 0.136	0.277 ± 0.112	0.192 ± 0.156	0.172 ± 0.127
Sphingomyelins	0.331 ± 0.108	0.280 ± 0.074	0.207 ± 0.121	0.217 ± 0.085
Sugars	0.290	0.069	0.262	0.020

The mean and standard deviation are reported

^a^His, Ile, Leu, Lys, Met, Phe, Thr, Trp, Val

^b^Ala, Arg, Asn, Gln, Glu, Gly, Pro, Ser, Tyr

### GWAS for metabolite traits in pigs identified numerous mQTL

**Fig. 2:**
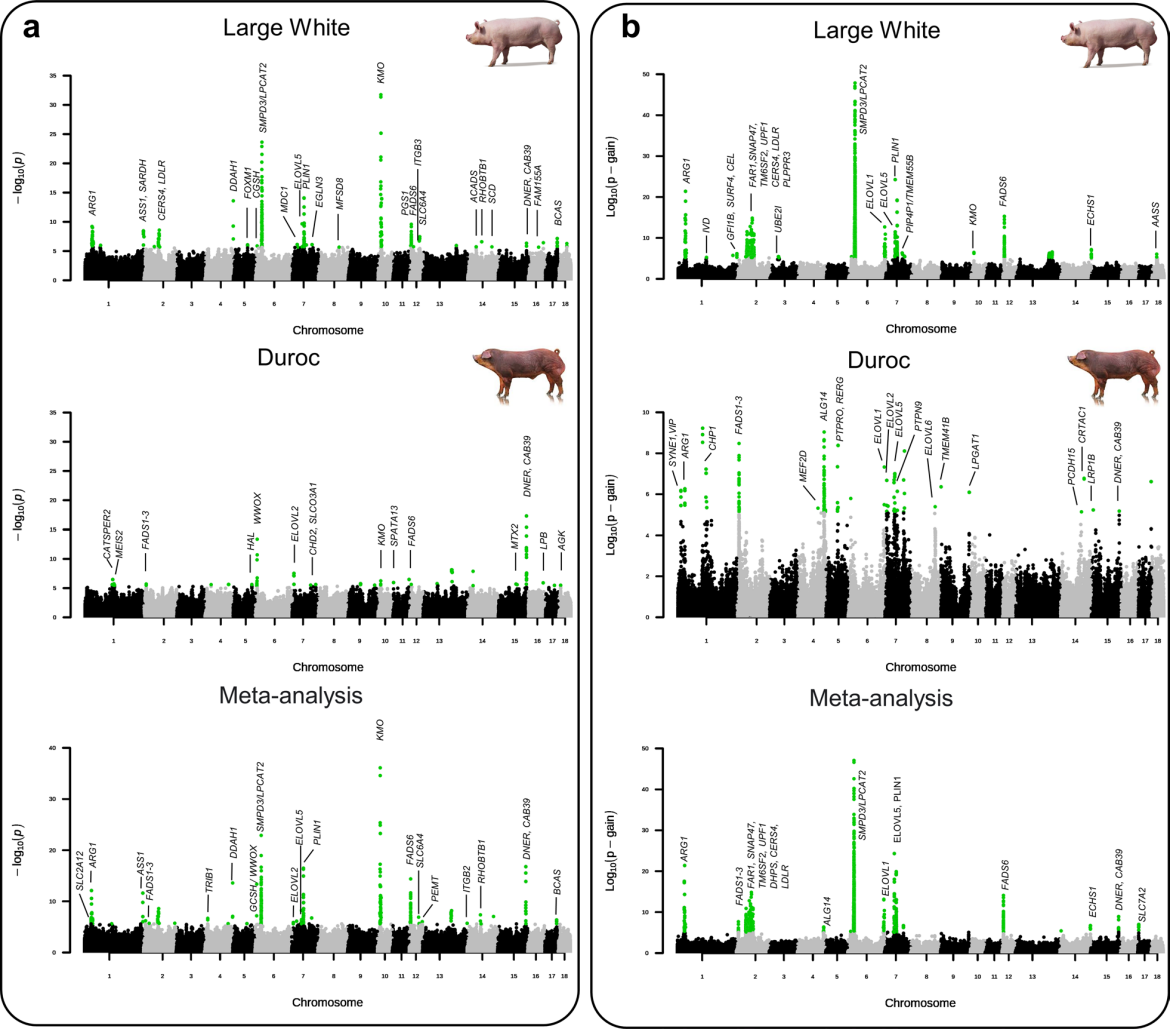
Joint Manhattan plots summarizing the results of the GWAS. GWAS are reported for the single metabolites and their ratios in the two breeds. **a** GWAS for single metabolite levels in Large White pigs, Duroc pigs, and in meta-analysis. **b** GWAS for metabolite ratio levels in Large White pigs, Duroc pigs, and in meta-analysis. Each dot represents a SNP. Suggestive and significant associations are highlighted in green. The candidate genes at the identified mQTL are listed above the signals

We initially conducted GWAS for the concentration of the analyzed metabolites separately in Large White pigs and Duroc pigs, followed by a combined meta-analysis. In Large White pigs we identified significant associations for 56 metabolites (accounting for 68 association signals, totaling 33 different mQTL) and in Duroc pigs for 22 metabolites (accounting for 25 association signals, totaling 24 different mQTL) (see Table 2 and Additional file 1: Table S9). Nine metabolites showed significant associations in both breeds: for 6 of these, different mQTL were identified in the 2 breeds, while for the other 3 metabolites, the same locus was identified in both breeds (Fig. 2a). The meta-analysis provided associations for a total of 42 metabolites with 48 different associations across 26 mQTL. Among these associations, 14 were found to be novel in the combined dataset (Fig. 2a; see Additional file 1: Table S10), while 26, 5, and 3 were also identified in, respectively, the Large White, the Duroc, and in both breeds (Table 2).

**Table 2:** Summary of genetic association signals for metabolites (mQTL) identified in Large White and Duroc pigs

SSC:position^a^	Single metabolite analyses			Metabolite ratio analyses			Candidate gene(s)^h^
	Metabolite^b^	*P*^c^	GWAS^d^	Metabolite ratios^e^	*p*-gain^f^	GWAS^g^	
1:13637577	–	–	–	PC aa C32:0/PC aa C34:4; PC aa C32:0/PC ae C32:2	1.52 × 10^6^	D	*SYNE1*^r^, *VIP*^r^
1:30232849	Ile	2.08 × 10^–07^	Meta	–	–	–	*SLC2A12*^t^
1:31868855	Orn, Arg	7.71 × 10^–13^	Meta, LW	Arg/Orn; Arg/Lys	2.49 × 10^21^	LW, D, Meta	*ARG1*^e^
1:127957580	PC ae C34:3	3.39 × 10^–07^	D	–	–	–	*CATSPER2*^r^
1:130488207	–	–	–	SM C16:0/SM C18:0	1.67 × 10^7^	D	*CHP1*^r^
1:131201231	–	–	–	C4/C5	1.78 × 10^5^	LW	*IVD*^e^
1:133696865	PC ae C34:3	1.83 × 10^–06^	D	–	–	–	*MEIS2*^r^
1:270725631	Cit	1.44 × 10^–12^	Meta, LW	–	–	–	*ASS1*^e^
1:272712128	–	–	–	PC ae C38:4/PC ae C40:5	1.44 × 10^6^	LW	*GFI1B*^r^, *SURF4*^r^, *CEL*^e^
1:273242436	Sarcosine	3.49 × 10^–08^	LW	–	–	–	*SARDH*^e^
2:9548890	PC ae C42:0	6.08 × 10^–07^	Meta, D	PC aa C38:3/PC aa C40:4 (9)	3.01 × 10^8^	D	*FADS1*^e^, *FADS2*^e^, *FADS3*^e^
2:45628435	SM C18:0	1.70 × 10^–06^	LW	PC aa C32:0/PC ae C34:1 (37)	3.42 × 10^11^	LW, Meta	*FAR1*^e^
2:50110622	–	–	–	SM C16:1/SM C18:1 (1)	1.02 × 10^08^	LW, Meta	*SNAP47*^r^
2:60455290	–	–	–	SM C16:1/SM C18:1 (5)	6.30 × 10^12^	LW, Meta	*TM6SF2*^r^, *UPF1*^r^
2:66008692	SM C18:1, SM C18:0	3.0 × 10^–07^	LW	SM C16:1/SM C18:1 (10)	3.94 × 10^10^	LW, Meta	*DHPS*^e^
2:70649393	SM C18:0, SM C18:1	2.60 × 10^–09^	LW	SM C16:1/SM C18:1 (9)	7.04 × 10^14^	LW, Meta	*CERS4*^e^, *LDLR*^r^
2:77810469	–	–	–	SM C16:1/SM C18:1 (5)	9.40 × 10^10^	LW	*PLPPR3*^r^
3:40354181	–	–	–	ADMA/total-DMA	2.74 × 10^5^	LW	*UBE2I*^e^
4:14576462	SM C20:2	2.07 × 10^–07^	Meta	–	–	–	*TRIB1*^r^
4:93440441	–	–	–	PC ae C38:2/SM (OH) C14:1	2.07 × 10^5^	D	MEF2D^r^
4:122058519	–	–	–	PC aa C34:1/PC aa C36:1 (4)	1.09 × 10^9^	D, Meta	*ALG14*^e^
4:130543416	ADMA, total-DMA	2.55 × 10^–14^	LW, Meta	–	–	–	*DDAH1*^e^
5:56960624	–	–	–	PC aa C32:3/PC aa C36:1 (2)	2.41 × 10^8^	D	*PTPRO*^r^, *RERG*^r^
5:66734388	Alpha-AAA, Ile, Phe, kynurenine*	8.48 × 10^–07^	LW, Meta	–	–	–	*TSPAN9*^r^, *TSPAN11*^r^
5:87611954	His	2.44 × 10^–06^	D	–	–	–	*HAL*^e^
6:7037256	Gly	1.30 × 10^–06^	LW	–	–	–	*GCSH*^e^
6:8449207	Ac-Orn	4.29 × 10^–14^	D, Meta	–	–	–	*WWOX*^r^
6:29486603	PC aa C40:5 (other 12)	2.35 × 10^–24^	LW, Meta	PC aa C38:4/PC aa C38:5 (459)	7.37 × 10^47^	LW, Meta	*LPCAT2*^e^, *SMPD3*^e^
6:167797522	–	–	–	SM (OH) C22:1/SM C24:0 (7)	1.40 × 10^13^	Meta, LW, D	*ELOVL1*^e^
7:8566130	PC aa C42:5, PC aa C40:6	2.95 × 10^–08^	D, Meta	PC ae C38:5/PC ae C40:6; PC aa C38:4/PC aa C42:5	4.73 × 10^6^	D	*ELOVL2*^e^
7:23110906	Cit	7.57 × 10^–07^	LW	–	–	–	*MDC1*^r^
7:45991916	PC ae C44:6, PC ae C40:1	2.21 × 10^–10^	LW, Meta	PC ae C44:5/PC ae C44:6 (18)	2.07 × 10^24^	Meta, LW, D	*ELOVL5*^e^
7:54919473	PC ae C40:1 (other 5)	3.31 × 10^–17^	Meta, LW	PC aa C38:4/PC ae C42:2 (26)	7.78 × 10^19^	Meta, LW	*PLIN1*^r^
7:57650479	–	–	–	PC aa C42:0/PC ae C40:1 (1)	1.38 × 10^6^	D	*PTPN9*^r^
7:65570498	Pro	9.04 × 10^–07^	LW	–	–	–	*EGLN3*^r^
7:78429656	–	–	–	PC aa C38:0/PC ae C40:1	2.13 × 10^6^	LW	*PIP4P1*^e^, *TMEM55B*^e^
7:86348536	LysoPC a C16:0	3.09 × 10^–06^	D	PC ae C34:1/PC ae C38:6	4.5 × 10^6^	D	*CHD2*^r^, *SLCO3A1*^r^
7:89764753	–	–	–	PC ae C34:2/PC ae C36:5 (4)	5.23 × 10^6^	Meta, LW	*TMEM229B*^r^
8:96490961	LysoPC a C20:3	2.11 × 10^–06^	LW	–	–	–	*MFSD8*^t^
8:111825944	–	–	–	PC aa C32:2/SM C18:1	2.44 × 10^5^	D	*ELOVL6*^e^, *EGF*^r^
9:482531	–	–	–	PC ae C36:1/PC ae C36:2	2.29 × 10^6^	D	*TMEM41B*^r^
10:12447567	Kynurenine	7.88 × 10^–37^	Meta, LW, D	Phe/kynurenine	2.97 × 10^6^	LW	*KMO*^e^
11:2901405	LysoPC a C16:0	1.11 × 10^–06^	D	–	–	–	*SPATA13*^r^
12:3934836	C0	1.17 × 10^–06^	LW	–	–	–	*PGS1*^e^
12:6356756	PC ae C40:6 (10)	4.09 × 10^–15^	Meta, LW	PC ae C36:5/PC ae C38:6 (32)	2.01 × 10^15^	LW, Meta	*FADS6*^e^
12:16761024	PC ae C42:5	1.80 × 10^–06^	LW	–	–	–	*ITGB3*^r^
12:45808302	Serotonin	3.47 × 10^–08^	LW, Meta	–	–	–	*SLC6A4*^t^
12:60396907	PC aa C38:4 (PC aa C34:4)	9.22 × 10^–07^	Meta	–	–	–	*PEMT*^e^
13:207771882	LysoPC a C20:4	1.95 × 10^–06^	Meta	–	–	–	*ITGB2*^r^
14:64397658	PC ae C44:5	2.57 × 10^–07^	LW, Meta	–	–	–	*RHOBTB1*^r^
14:96845763	–	–	–	PC aa C32:3/PC ae C38:1	1.37 × 10^5^	D	*PCDH15*^r^
14:108968359	–	–	–	PC aa C32:3/PC aa C36:1	5.98 × 10^6^	D	*CRTAC1*^r^
14:111732013	SM C20:2	1.89 × 10^–06^	LW	–	–	–	*SCD*^e^
14:122538710	PC aa C40:4	9.05 × 10^–08^	Meta	–	–	–	*GPAM*^e^, *ACSL5*^e^
14:141410475	–	–	–	SM (OH) C24:1/SM C16:0 (10)	1.48 × 10^7^	LW	*ECHS1*^e^
15:10146862	–	–	–	lysoPC a C18:0/PC aa C34:2	1.70 × 10^5^	D	*LRP1B*^r^
15:82279524	Alpha-AAA	2.01 × 10^–06^	D	–	–	–	*MTX2*^r^
15:131052021	C18:2	4.99 × 10^–18^	D, LW, Meta	C14:1/C18:2; C16/C18:2	8.24 × 10^8^	Meta	*DNER*^r^, *CAB39*^r^
16:47077521	C3	2.16 × 10^–06^	LW	–	–	–	*FAM155A*^r^
17:5154217	–	–	–	Arg/Thr; Lys/Thr	9.92 × 10^6^	Meta	*SLC7A2*^t^
17:41835566	PC aa C42:5	3.39 × 10^–06^	D	–	–	–	*LBP*^r^
17:54676990	LysoPC a C16:1 (other 3)	7.13 × 10^–08^	LW, Meta	–	–	–	*BCAS1*^r^
18:8294159	PC aa C36:5	3.21 × 10^–06^	D	–	–	–	*AGK*^e^
18:22275108	–	–	–	Lys/Met; Lys/Orn	1.10 × 10^6^	LW	*AASS*^e^

The reported results are from single metabolite and metabolite ratio analyses that pointed at some candidate genes. The complete list of all 97 mQTL and other details are given in Additional file 1, Table S11

^a^*Sus scrofa* chromosome (SSC) and the position of the most significant marker on Sscrofa11.1 genome version

^b^The names or acronyms of the most significant metabolites for the corresponding mQTL region identified by the reported tag SNP are listed. When more than one metabolite was significant, the metabolites are listed from the most significant. If more than four metabolites have been identified for the same mQTL, the number of significant metabolites in addition to the top one is indicated in parenthesis

^c^At each mQTL, the *P* of association is reported for the most significant metabolite for the indicated chromosome variant position

^d^Significant results obtained in the GWAS for the Large White breed (LW), Duroc breed (D) and in the meta-analysis (Meta) (listed from the most significant, when more than one reported significant results)

^e^The most significant ratios are reported. When more than two significant ratios were identified for the corresponding QTL, the number of additional ratios is reported in parenthesis

^f^The highest p-gain value (most significant results in the ratio analyses) is reported

^g^Significant results obtained in the GWAS for the Large White breed (LW), Duroc breed (D) and in the meta-analysis (Meta) (listed from the GWAS that reported the most significant results, when more than one analyses reported significant results)

^h^The candidate genes potentially explaining a functional effect on the identified mQTL are defined as indicated in Methods. The type of encoded protein by the candidate genes is also indicated: r = regulatory; e = enzyme; t = transporter

Summarizing the results of these three GWAS, associated SNPs were identified for ~ 46% of the investigated metabolites, totaling 63 different mQTL. Multiple loci were identified for 23 metabolites (up to 4 for citrulline). One locus on *Sus scrofa* chromosome (SSC) 6, one on SSC7, and one on SSC12 were associated with the concentration of 13 glycerophospholipids (all in Large White pigs and meta-analysis), 6 glycerophospholipids (identified in meta-analysis and Large White pigs), and 11 glycerophospholipids (one also in Duroc pigs), respectively. On average, the percentage of variance explained (PVE) for the peak SNP underlying the identified mQTL was 5.45 ± 3.40%, with higher values observed in Duroc (mean = 9.45 ± 3.51%) compared to Large White pigs (mean = 4.26 ± 2.26%): the top three values were for acylcarnitine C18:2 (23.1%) and acetylornithine (Ac-Orn: 18.1%) in Duroc pigs and kynurenine in Large White pigs (16.5%). The most significant association was found for kynurenine with SNPs on SSC10, in both the Large White breed and in the meta-analysis (*P* = 1.83 × 10^–32^ and *P* = 7.88 × 10^–37^). The most significant association in Duroc pigs was identified for acylcarnitine C18:2 on SSC15 (*P* = 4.99 × 10^–18^), again matching the highest values of the PVE for the same SNP [see Additional file 1: Table S9].

To confirm and further expand these results, we also analyzed the ratios of metabolite concentrations as metabolite traits. Similar studies in humans have already shown that these combined metabotypes can significantly reduce variation [2]. This is especially true when a pair of metabolites are closely interdependent, acting as substrates and/or products in the same enzymatic reaction or pathway or are connected through a common regulatory system [2]. A total of 594 and 39 ratios showed significant associations (considering a stringent *p*-gain threshold; Fig. 2b) in Large White and Duroc populations, respectively, which identified a total of 49 mQTL. Of these, 34 were not previously identified with the single metabolite approach: 15 in Large White, 4 of which were also found in the meta-analysis; 18 in Duroc, 2 of which were also found in meta-analysis; and one only in the meta-analysis (see Additional file 1: Tables S9 and S10). Of the large number of significant mQTL identified for Large White pigs, many ratios involved at least one glycerophospholipid (570 ratios). Most of these ratios (n. 470) were identified for the same SSC6 region that was also associated with single glycerophospholipids, further supporting the presence of a major mQTL in this chromosome region that affects the metabolism of these molecules.

By combing the results obtained for the metabolite ratios with those obtained for single metabolites, we identified a total of 97 mQTL (Table 2; see Additional file 1: Table S11) for 126 metabolites, which accounted for 72.4% of the analyzed molecules: 18 mQTL were associated with amino acids; 9 with acylcarnitines; 13 with biogenic amines; 38 with phosphatidylcholines; 13 with lysophosphatidylcholines; and 20 with sphingomyelins.

### Putative causal genes for mQTL involved in biochemical and regulatory pathways

To prioritize likely causal genes for the identified mQTL, we adapted the strategies proposed by previous GWAS for metabolites in humans (e.g., [52, 53]). We retrieved information from: (i) a hypothesis-free genetic approach based on genomic annotations within 1 Mb centered at the lead SNP; (ii) specific metabolite-gene associations obtained from 6 databases and a manually curated literature survey. Since most of the available biological knowledge is derived from human studies, we utilized this information to conduct a first human-pig comparative analysis of GIM, as outlined below. From this analysis, approximately two thirds of the mQTL identified potential causal genes: one gene was deemed plausible for 51 mQTL, while for the remaining 13 mQTL, 2 or 3 genes could be equally plausible. Thirty-four of these genes encode enzymes that are directly involved in metabolic processes/transformations that include the associated metabolites, 4 encode transporters and the remaining genes encode regulatory proteins/signaling transmitters or structural elements. For the other 33 mQTL, as no obvious candidates could be identified, novel causal genes not yet described in other species may be included in the highlighted genomic regions.

We can list a few examples of candidate genes for the identified mQTL. Among the mQTL for the concentration of a few amino acids, 6 genes encode enzymes or transporters that may directly alter the corresponding amino acid concentration or ratio (Table 2): (i) arginase 1 (*ARG1*, on SSC1), which encodes the enzyme that catalyzes the hydrolysis of arginine to ornithine and urea, may explain an mQTL for the level of arginine and ornithine and their ratio; (ii) argininosuccinate synthase 1 (*ASS1*, on SSC1), which encodes the enyme that catalyzes the formation of arginosuccinate from aspartate, citrulline and ATP, may explain an mQTL, for the level of citrulline; (iii) histidine ammonia-lyase (*HAL*, on SSC5), which encodes the enzyme that catalyzes the first reaction of the histidine catabolism, may explain an mQTL for the level of histidine; (iv) glycine cleavage system protein H (*GCSH*, on SSC6), which encodes the transporter that shuttles the methylamine group of glycine from the P protein (GLDC) to the T protein (GCST), may explain an mQTL for the level of glycine; (v) solute carrier family 7 member 2 (*SLC7A2*, on SSC17), which encodes a cationic amino acid transporter responsible for the uptake of arginine (Arg), lysine (Lys) and ornithine (Orn), may explain an mQTL for the Arg/Threonine (Thr) and Lys/Thr ratios; (vi) aminoadipate-semialdehyde synthase (*AASS*, on SSC18), which encodes the enzyme that catalyzes the first 2 steps in the lysine degradation pathway may explain an mQTL for the Lys/Met and Lys/Orn ratios.

The ratio between 2 acylcarnitines (butyrylcarnitine and valerylcarnitine; C4/C5) was found to be associated with SNPs near the isovaleryl-CoA dehydrogenase gene (*IVD*, on SSC1). This gene encodes the enzyme responsible for catalyzing the third step of leucine catabolism.

The mQTL identified for several biogenic amines may involve candidate genes found in the considered genomic window (Table 2): (i) sarcosine dehydrogenase (*SARDH*, on SSC1), associated with the level of sarcosine, encodes a mitochondrial enzyme that catalyzes the oxidative demethylation of sarcosine; (ii) dimethylarginine dimethylaminohydrolase 1 (*DDAH1*, on SSC4), associated with the level of asymmetric dimethylarginine (ADMA), encodes the enzyme that hydrolyzes ADMA; (iii) kynurenine 3-monooxygenase (*KMO*, on SSC10), associated with the level of kynurenine, encodes a key enzyme in tryptophan catabolism, which catalyzes the hydroxylation of l-kynurenine to form 3-hydroxy-l-kynurenine; (iv) solute carrier family 6 member 4 (*SLC6A4*, on SSC12), also known as 5-hydroxytryptamine (serotonin) transporter (*5-HTT*), affecting the level of serotonin, encodes an integral membrane protein that transports this neurotransmitter from synaptic spaces into presynaptic neurons.

mQTL identified for the levels of different lipid groups (phosphatidylcholines, lysophospatidylcholines and sphingomyelins) enabled the identification of several genes that encode enzymes involved in various metabolic pathways of these metabolite groups (Table 2): (i) carboxyl ester lipase (*CEL*, on SSC1), which encodes a pancreatic enzyme that catalyzes the hydrolysis of a wide range of lipid substrates; (ii) members of the fatty acid desaturase gene family on SSC2 (*FADS1*/*FADS2*/ *FADS3*) and SSC12 (*FADS6*), which encode desaturase enzymes that regulate the unsaturation of fatty acids; (iii) fatty acyl-CoA reductase 1 (*FAR1*, on SSC2), identified through the ratios between glycerophospholipids, which encodes an enzyme involved in the reduction of saturated and unsaturated fatty acyl-CoA to fatty alcohols; (iv) low density lipoprotein receptor (*LDLR*), which encodes the receptor for the major cholesterol-carrying lipoprotein of plasma, known to be involved in sphingomyelin regulation, may explain an mQTL on SSC2, together with the closely located ceramide synthase 4 (*CERS4*), which encodes an endoplasmic reticulum membrane component with sphingosine *N*-acyltransferase activity involved in sphingolipid metabolism; (v) lysophosphatidylcholine acyltransferase 2 (*LPCAT2*) and sphingomyelin phosphodiesterase 3 (*SMPD3*), both in the SSC6 region associated with the largest number of glycerophospholipid ratios (and ratios with sphingomyelins), which encode an enzyme exhibiting both acyltransferase and acetyltransferase activities involved in phosphatidylcholine acyl-chain remodeling and an enzyme that hydrolyzes sphingosylphosphocholines, respectively; (vi) members of the ELOVL fatty acid elongase gene family (*ELOVL1* on SSC6, *ELOVL2* and *ELOVL5* on two SSC7 regions and *EVOVL6* on SSC8), which encode endoplasmic reticulum-bound enzymes that catalyze key reactions in the long-chain fatty acids elongation cycle; (vii) perilipin 1 (*PLIN1*, on SSC7), which encode a coat protein of lipid storage droplets that modulates adipocyte lipid metabolism; (viii) phosphatidylethanolamine N-methyltransferase (*PEMT*, on SSC12), which encodes the enzyme that converts intracellular choline and phosphatidylethanolamine to phosphatidylcholine in different processes; (ix) stearoyl-CoA desaturase (*SCD*, on SSC14), which encodes an enzyme involved in fatty acid biosynthesis; (x) enoyl-CoA hydratase, short chain 1 (*ECHS1*, on SSC14), which encodes an enzyme involved in the mitochondrial fatty acid beta-oxidation pathway.

### Profiling associations among metabolite groups reveals cascade effects of candidate genes

**Fig. 3:**
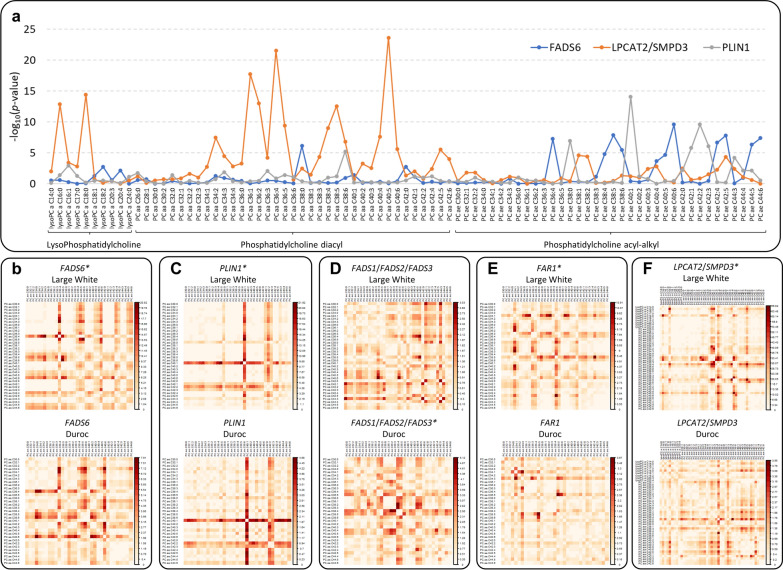
Profiles of associations at selected mQTL across metabolites and pairs of metabolites of the glycerophospholipid family. **a** The patterns are obtained for the mQTL regions encompassing the *FADS6*, *LPCAT2/SMPD3* and *PLIN1* genes in Large White pigs. The *P*-value of association is shown for the lead SNP of the respective mQTL. **b**–**f** Two-dimensional profiles in Large White and Duroc pigs for the candidate genes: *FADS6*, *PLIN1*, *FADS1/FADS2/FADS3*, *FAR1*, and *LPCAT2/SMPD3.* The − log_10_(*P*-value) is reported for acyl-alkyl phosphatidylcholines (PC ae Cx:x), lysophosphatidylcholines (lysoPC) and acyl-alkyl phosphatidylcholines (diacyl PC aa Cx:x). mQTL that were also identified with single metabolite analyses in one of the breeds are marked with an asterisk (*). All these mQTL were also identified in the single metabolite meta-analyses

Considering that the applied metabolomic approach can analyze various metabolites within the same subgroups, we then tested the hypothesis that association results could reveal information on the cascade effects of one locus across several related metabolites, as proposed by Rhee et al. [54]. For example, in Fig. 3a, the *P*-value for association is shown across 10 lysophosphatidylcholines and 73 phosphatidylcholines for the leading SNP for the mQTL with *FADS6*, *LPCAT2*/*SMPD3,* and *PLIN1* as identified candidate genes in Large White pigs. It can be observed from this figure that these 3 mQTL may have complementary roles in the overall profile of these metabolites, with little overlap, suggesting that they may influence different pathways, distinguished by the number of carbon atoms and the level of unsaturation of the analyzed molecules.

We then used two-dimensional representations based on metabolite ratios to disclose additional information about the cascade effects of gene markers on multiple metabolites (Fig. 3b–f; see Additional file 3: Figures S4–S8). For instance, in Duroc pigs, *FADS6* and *PLIN1*, which were not significantly associated with any metabolites in single metabolite analyses, were found to have similarities with the profile ratios observed in the Large White breed when using metabolite ratios. For both breeds, at the *FADS6* mQTL, the top ratios included the phosphatidylcholines PC ae C36:4 and PC ae C38 with 4 to 6 double bonds, and PC ae C38, with 4 to 6 double bonds*.* At the *PLIN1* mQTL, the top ratios were for PC ae C38:0, PC ae C40:1 and PC ae C42, with one to three double bonds. These two-dimensional pictures based on metabolite ratios confirmed the results of the meta-analyses, supporting the fact that the same two mQTL segregate in both breeds, although signals did not reach the significance threshold in Duroc breed, likely due to the lower power of the GWAS for this breed. For the mQTL identified in Duroc pigs in the correspondence of the *FADS1*/*FADS2*/*FADS3* genes, and in Large White pigs in the region of the *FAR1* and *LPCAT2*/*SMPD3* genes (all confirmed in meta-analyses), the two-dimensional pictures based on the metabolite ratios suggest that the identified mQTL may have different cascade effects in the two breeds. In Large White pigs, this ratio-based analysis identified clear effects of *FAR1* on PC ae C34:1, PC ae C36:3, and PC ae C38:5, which did not emerge in the single metabolite analysis. These findings further complement the effects on the glycerophospholipid family that have already been shown for *FADS6*, *LPCAT2*/*SMPD3,* and *PLIN1* using the single-marker analyses (Fig. 3a).

### GWAS results provide comparative pig-human insights

We compared the GWAS results we obtained in pigs with the results reported for the same metabolites in humans [7, 9–11, 55–59]. A summary of this comparison is presented in Additional file 4: Text S3. Out of the 64 mQTL for which we identified candidate genes, 41 included genes that were associated with the same metabolites or with metabolites of the same family in both pigs and humans. This provides indirect inter-species confirmation of our results and those previously identified in humans [see Additional file 1: Tables S11 and S12]. This can be also used to further support the candidacy of the reported genes which, in turn, may reveal some potential novel putative functions of the corresponding genes that have not yet been well defined. An example is the mQTL on SSC1 for isoleucine concentration, for which the solute carrier family 2 member 12 gene (*SLC2A12*) was identified as candidate gene. This gene encodes glucose transporter 12 (GLUT12), which acts as a sugar and urate transporter, suggesting that isoleucine could be a marker for the role of this transporter protein [60], as GWAS in humans have also reported that variability in this gene may affect blood isoleucine level [see Additional file 1: Tables S11, S12]. Another example comes from the mQTL on SSC1, which encompasses the vasoactive intestinal peptide gene (*VIP*), which is associated with phosphatidylcholine concentrations. Variability in the same gene in humans has been shown to be associated with the level of phosphatidylcholines [see Additional file 1: Table S11, S12]. VIP function has been shown to promote the synthesis of pulmonary surfactant phospholipids, which might be linked to the cascade pathways that regulate the production of the associated phosphatidylcholines [61, 62].

We also combined the GWAS results we obtained in pigs with other sources of information, which sheds new light on the putative function of several other genes. One example comes from the major facilitator superfamily domain containing 8 (*MFSD8*) gene located in a region on SSC8 that is associated with the level of a lysophosphatidylcholine. In humans, a form of the neuronal ceroid lipofuscinosis (neuronal ceroid lipofuscinosis 7, CLN7) is associated with pathogenic variants in *MFSD8*, which encodes an MFS transporter that moves small solutes (yet to be identified) across membranes [63, 64]. The major facilitator superfamily domain-containing protein 2A gene (*MFSD2A*), a close paralog of *MFSD8*, is known to be a component of the blood–brain barrier that transports lysophosphatidylcholines into the central nervous system [65]. Adding our results to the information derived from these other studies suggests that MFSD8 may use lysophosphatidylcholines as substrates that are actively transported from the blood to the brain. This hypothesis could potentially be useful in explaining the molecular mechanisms underlying CLN7 disease.

Multiple GWAS in humans have confirmed associations between metabolites and genes that we also report here for pigs. In most cases, these genes are well characterized, with well-established direct relationships to the associated metabolites (such as *ARG1*, *IVD*, *SARDH*, *FADS1*/*FADS2*/*FADS3*, *CERS4*, *DDAH1*, *HAL*, *GCSH*, *ELOVL2*, *KMO,* and *SLC7A2*), as previously described. However, in the case of the tribbles pseudokinase 1 gene (*TRIB1*), which we identified to be associated with sphingomyelins, and of the transmembrane protein 229B gene (*TMEM229B*), associated with phosphatidylcholine ratios in our results, the connections may be indirect, involving different regulatory pathways.

Several strong candidate genes with roles that have been already clearly defined have not been associated with the same metabolites in humans as we report here for pigs [Additional file 1: Table S11]. This list includes, several genes involved in the glycerophospholipid biosynthesis and fatty acid metabolism super-pathways: *FAR1*, associated in pigs with several phosphatidylcholine ratios; *LPCAT2* and *SMPD3*, included in the SSC6 mQTL with the largest number of glycerophospholipid ratios; *ELOVL1*, *ELOVL5,* and *ELOVL6* which are associated in pigs with sphingomyelins or phosphatidylcholines; *FADS6,* associated in pigs with phosphatidylcholines and various glycerophospholipid ratios; *SCD,* associated in pigs with sphingomyelins, whereas in humans it was associated with other lipids produced within the common glycerophospholipid biosynthesis super-pathway, i.e. phosphatidylcholines and lysophosphatidylcholines; and *ECHS1,* associated in pigs with sphingomyelins. Furthermore, variability in the *SLC6A4* gene region, which we identified to be associated with plasma serotonin levels in pigs, has not demonstrated the same association with this biogenic amine in any human biofluids, to the best of our knowledge.

### Identification of candidate causative mutations in pigs based on whole genome resequencing

We conducted whole genome resequencing for 88 Large White and 35 Duroc pigs from the same metabolized pig populations, as well as for additional 35 Landrace pigs for comparative analyses. Among the 1,420,757 variants [see Additional file 2, Table S13; Additional file 4: Text S4] we identified in the 97 mQTL regions, and considering those variants that alter the protein coding sequence [see Additional file 1: Table S14], only few of these potentially disrupting mutations had a moderate to high breed specific linkage disequilibrium (LD; *r*^2^ > 0.5) with the lead SNP for the corresponding mQTL. Therefore, only variants in 6 genes (missense mutations in *ARG1*, *GFI1B*, *PTPRO*, *MDC1*, and *FADS6,* and one in-frame insertion in *KMO*) can be considered compatible with a putative causative role for the identified mQTL based on their estimated minor allele frequency, LD level, and potential functional effects on the encoded protein (Table 3).

**Table 3:** Relevant variants identified in the candidate genes

mQTL^a^	SSC:position^b^	Ref/Alt^c^	rs number	Gene	Consequence^d^	Protein position^e^	SAP^f^	Genotypes^g^		LD (*r*^2^)^h^	
								Large White	Duroc	Large White	Duroc
3	1:32016023	G/A	rs320005747	*ARG1*	Missense	31	S/L (deleterious)	52/28/8	20/14/1	0.562^*^	0.537*
13	1:272703230	C/T	rs325802595	*GFI1B*	Missense	86	A/V (deleterious)	16/34/38	1/14/20	0.709*	1.000
33	5:57031576	T/TC	–	*PTPRO*	Frameshift	1110	E/GX	0/2/86	1/10/24	1.000	1.000*
44	7:23235098	T/C	rs331543534	*MDC1*	Missense	1323	T/A (deleterious)	56/25/4	35/0/0	0.703*	-
44	7:23240513	C/A	rs691602895	*MDC1*	Missense	331	A/S (deleterious)	54/30/4	35/0/0	0.819*	-
63	10:12489135	A/ACCT	**-**	*KMO*	In-frame insertion	439	Y/YL	67/19/2	2/13/20	1.000*	1.000^*^
67	12:6362988	A/G	rs342255296	*FADS6*	Missense	126	K/E (deleterious)	48/37/3	22/13/0	1.000^*^	1.000^*^

Reported information includes minor allele frequency, linkage disequilibrium level, and/or consequence on the encoded protein that could be compatible with a functional role for the corresponding identified mQTL

^a^mQTL no. reported in Additional file 2: Table S11

^b^*Sus scrofa* chromosome (SSC): position of the reference nucleotide (based on the Sscrofa11.1 reference genome)

^c^Reference allele (Sscrofa11.1)/alternative allele

^d^Consequence of the alternative allele as derived from the Variant Predictor Effect (VEP)

^e^Positions refer to the following UniProt accessions (accessed on: 28 March 2024): A0A5S8LD81 (*ARG1*), F1S0S2 (*GFI1B*), F1RKS7 (*FADS2*), F1SQX1 (*PTPRO*), A0A5G2QL96 (*MDC1*), Q9MZS9 (*KMO*) and A0A8D0V8P9 (*FADS6*)

^f^Single amino acid polymorphism: prediction of the effect with SIFT, when the information was available for the missense mutations

^g^No. of pigs carrying the three possible genotypes based on the whole-genome sequenced animals. Genotypes are: homozygous for the reference allele/heterozygous/homozygous for the alternative allele

^h^Linkage Disequilibrium (LD) measured between the variant and the top associated SNP pointing out the reported gene as candidate

*The corresponding mQTL has been identified in the indicated breed (based on single marker, ratio and/or meta-analyses)

Focusing on the sequence structure of the *KMO* gene, the in-frame insertion that segregates in the three cosmopolitan breeds sequenced in this study (Large White, Duroc, and Landrace) had an LD value of 1.00 with the most significant SSC10 SNP for the level of kynurenine and with 2 other *KMO* missense mutations that were present in all three breeds [see Additional file 2: Table S15]. These findings indicate that two major haplotypes (named rs81278711-A and rs81278711-G, according to the tag SNP associated with the level of kynurenine in the GWAS) are present at the *KMO* in these three breeds. These *KMO* haplotypes have opposite frequencies in the Large White and Duroc breeds [see Additional file 2: Tables S15 and S16].

We also report the frequencies of *KMO* nonsynonymous mutations in 22 different pig breeds (including the 3 cosmopolitan breeds: Large White, Duroc and Landrace; and 19 autochthonous breeds from 9 European countries) and in European wild boars [see Additional file 2: Table S16]. The most frequent haplotype presents in the Large White breed (rs81278711-A) may be considered the wild-type form, as it was fixed in European wild boars. This form was the most frequent in most local breeds, except for the Mora Romagnola breed, which experienced introgression from Duroc pigs in the past [45].

### A case study: *KMO* haplotypes affect metabolites of the kynurenine pathway based on a nutrigenetic study

**Fig. 4:**
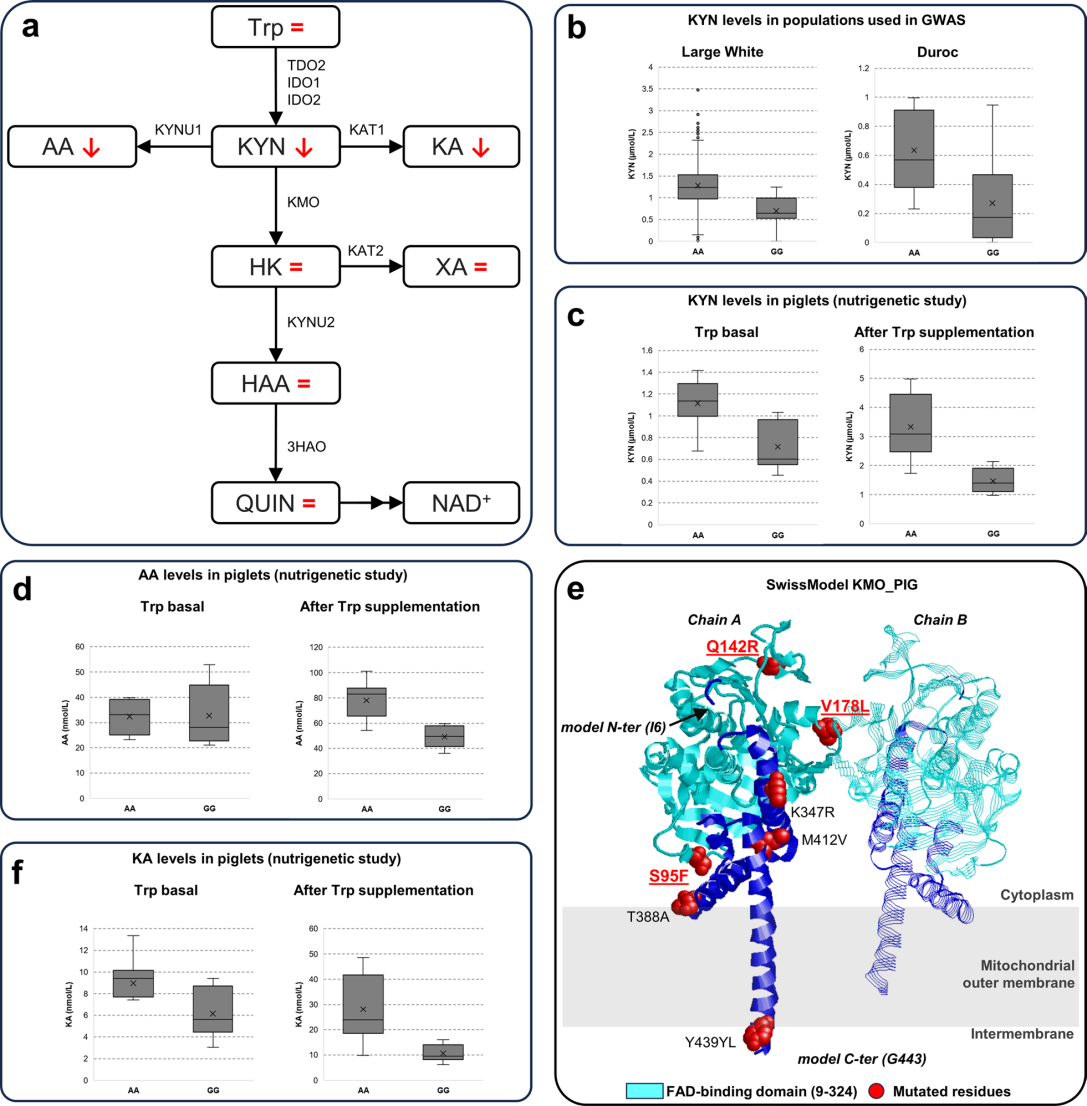
Information on the kynurenine pathway and KMO. **a** Simplified representation of the kynurenine pathway. Metabolites are shown in boxes and reaction directions are shown by arrows labelled with enzyme acronyms. The red symbols “↓”, “↑” and “ = ” indicate the decrease, increase, and equal metabolite concentration in piglets homozygous for the *KMO* rs81278711-G or the rs81278711-A alleles, as observed in the longitudinal nutrigenetic study (the SNP refers to that associated with kynurenine level in the GWAS; these two alleles indicate the two major *KMO* haplotypes). Abbreviations for the metabolites: Trp, tryptophan; KYN, kynurenine; XA, xanthurenic acid; AA, anthranilic acid; KA, kynurenic acid; HK, 3-hydroxykynurenine; HAA, 3-hydroxyanthranilic acid; QUIN, quinolinic acid. Abbreviations for the enzymes: TDO, tryptophan 2,3-dioxygenase; KYNU1, kynureninase; KYNU2, kynureninase; KMO, kynurenine 3-monoxygenase; KAT1, kynurenine aminotransferase; KAT2, kynurenine aminotransferase; 3HAO, 3-hydroxyanthranilate 3,4-dioxygenase. **b**, **c** Plasma kynurenine (KYN) concentration in Large White and Duroc pigs included in the GWAS and in the Large White × Landrace piglets homozygous for the alternative *KMO* alleles. **d**, **e** Plasma anthranilic acid (AA) and kynurenic acid (KA) concentrations in the piglets homozygous for the alternative *KMO* haplotypes. **f** Model of the pig KMO protein as retrieved from the SwissModel repository and based on rat KMO (PDB: 6LKD). Chains A and B of the functional dimeric structure are shown in cartoons and ribbons representations, respectively. The FAD-binding domain detected by the PFAM entry PF01494 is depicted in cyan. Positions corresponding to the differences in the two *KMO* haplotypes are represented in spacefill and coloured in red. Variations (S95F, Q135R, and V178L) occurring in the FAD-binding domain are evidenced

The kynurenine (KYN) pathway (KP) is an alternate tryptophan (Trp) catabolic pathway that, under physiological conditions, accounts for ~ 95% of the overall breakdown of this essential amino acid, resulting in the downstream production of KYN and other immunoregulatory and neuroactive metabolites, including the important redox cofactor nicotinamide adenine dinucleotide (NAD) [66]. In this pathway, Trp is converted into KYN, which is then metabolized through three routes. One of these routes involves conversion of KYN into 3-hydroxykynurenine (HK) by the enzyme kynurenine 3-monoxygenase (KMO). This conversion leads to the production of quinolinic acid (QUIN), which is further transformed into NAD. A simplified representation of the KP is shown in Fig. 4a.

After identifying *KMO* as the candidate gene affecting the level of KYN in pig plasma (Fig. 4b), we conducted a longitudinal nutrigenetic study to examine the impact of the two specific *KMO* haplotypes on KYN and several other metabolites of the same pathways. We used the targeted Bevital platform (see Additional file 2: Table S4; the Biocrates platform did not include all these metabolites) [49] to measure all major KP intermediate metabolites in the plasma of two groups of eight weaned Large White × Landrace piglets. Each group was homozygous for one of the two *KMO* haplotypes (rs81278711-A and rs81278711-G), which correspond to two different KMO deduced protein forms, as determined by a few single amino acid polymorphisms [see Additional file 2: Table S15].

All animals were subsequently fed with standard diet and then with a double diet supplementation of Trp to potentially boost the putative effects of the 2 *KMO* haplotypes on the KP components in relation to the basal state. At both basal and Trp supplementation time points, the piglets of the two groups had different plasma KYN concentrations, in the same direction as expected based on the GWAS results, with the rs81278711-AA genotype having a higher KYN level than the rs81278711-GG genotype (Fig. 4b, c).

The Trp load in the piglets’ diet magnified the difference in KYN concentration between the animals carrying the two opposite genotypes (*P* = 6.22 × 10^–04^), with a large increase of KYN in the rs81278711-GG piglets. The same effect was also evident on the levels of kynurenic acid (KA) and anthranilic acid (AA) (Fig. 4d, e; see Additional file 2: Table S4), which defines the two alternative routes of transformation of KYN that are not catalyzed by KMO. The level of 3-hydroxykynurenine (HK), whose production from KYN is catalyzed by KMO, did not differ between the piglets with different genotypes. The same was true for all other metabolites [see Additional file 2: Table S4; Additional file 3: Figures S9 and S10]. Given these results, using a steady-state kinetic model, we tested whether the most parsimonious explanation for the differences in the levels of the considered metabolites in the two groups of piglets based on their different *KMO* haplotypes could be attributed to differences in the reaction rate (velocity) of the KMO enzyme (*v*_KMO_). Specifically, the model assessed whether an increase of *v*_KMO_ in the KMO^rs81278711−GG^ pigs could result in the observed differences in KYN levels (as well as KA and AA) [see Additional file 2: Tables S4 and S6; Additional file 3: Figures S9-S11] due to an increased production rate of HK. This modeling indicated that the observed differences could be due to differences in one of the following parameters: the affinity for the substrate (*K*_M_), the turnover number (*k*_cat_), or the total amount of enzyme ([E]). We then analyzed *KMO* gene expression and protein levels in the liver (the main tissue involved in the KP) of pigs with the two *KMO* genotypes, using qPCR and Western blotting. No differences were observed in both analyses between the two groups of pigs [see Additional file 3: Figure S12], ruling out the possibility that the estimated difference in *v*_KMO_ could be attributed to differences in [E]. In silico modeling and investigation of the KMO protein structure revealed three variations that characterize the two haplotypes (S95F, Q135R, and V178L), all three in the FAD-binding domain (Fig. 4f; Additional file 3: Figure S13; Additional file 4: Text S5). Therefore, considering all these results, it is reasonable to suggest that the differences in the two KMO protein forms, as defined by the 2 haplotypes, can alter metabolite affinity to the encoded enzyme or affect the cofactor binding and, therefore, the enzyme kinetics.

### Gaussian graphical models can reconstruct metabolic pathways in pigs

**Fig. 5:**
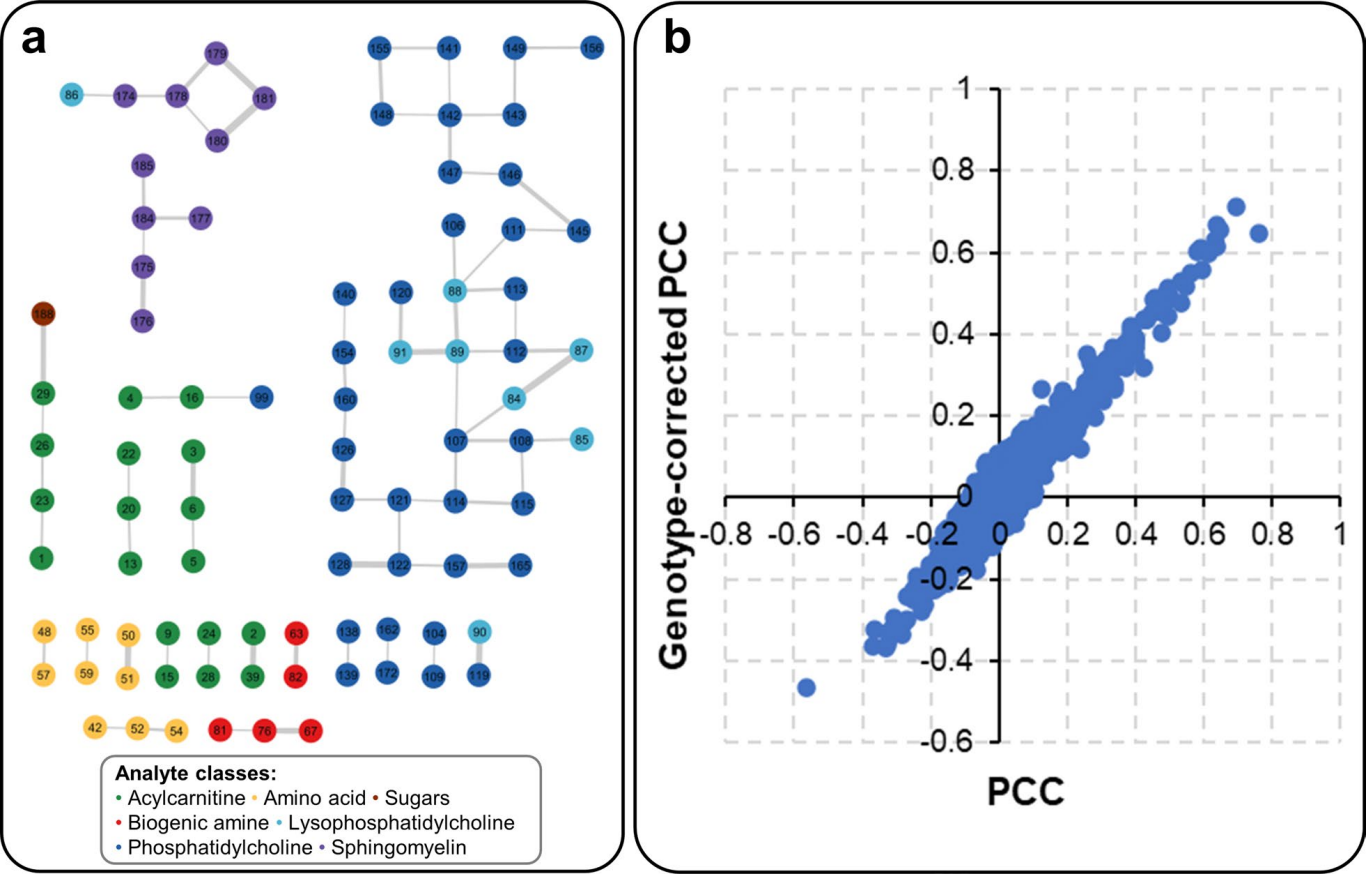
Gaussian Graphical Model (GGM) obtained using metabolomic information in pigs. **a** Network presentation of Partial Correlation Coefficients (PCC) > 0.3 (88 metabolites connected by 73 edges) in the Large White and Duroc populations. Singletons (n = 71) are not shown. Each node represents a metabolite, and the line width of edges indicates the PCC strength. Node labels are given in Additional file 1: Table S1. **b** Comparison between PCC in Large White pigs when excluding and including the genetic effects defined by the identified mQTL

Following the reasoning that metabolomic data can be used to deduce biochemical information, we went back to the 164 metabolites from the 6 analyte classes that were measured using the Biocrates approach in all Large White and Duroc pigs. Our goal was to identify correlations between metabolites and construct a pig-specific Gaussian Graphical Model (GGM). In GGM, edges represent correlations between two variables conditional on all other variables (i.e., all metabolites not included in the pair) to calculate Partial Correlation Coefficients (PCC) [67]. High PCC values between metabolite pairs typically indicate closely related metabolites that are separated by one or a few enzymatic steps (pathway distance = 1, 2, 3…). This allows for the reconstruction of known (as proof-of-concept) or unknown (discovery) steps and biological connections in metabolic pathways, including co-regulations [29, 42]. The GGM consisted of a total of 159 nodes: 88 metabolites connected by 73 edges (PCC > 0.3) and 71 singletons (Fig. 5a). Integrating GWAS information into the GGM did not significantly alter the metabolite networks obtained (Fig. 5b; see Additional file 2: Table S17; Additional file 3: Figure S14; Additional file 4: Text S6). Several examples on the inclusion of the effect of specific mQTL genotypes in estimation of metabolite correlations are shown in Additional file 3: Figures S15 and S16.

The 73 significant PCC of the GGM obtained with all pigs are reported in Table 4 and Additional file 1: Table S18, along with comparative information from similar human metabolomic datasets retrieved from previous studies [55, 56, 67]. The proposed metabolic pathway explanations are also included. As expected, metabolites of the same family tended to be more interconnected with each other than with metabolites of other classes, as also observed in previous studies [55, 56, 67]. Only 3 of the 73 significant PCC included metabolites from different families [see Additional file 1: Table S18]. Several detailed examples of partial networks that include closely related metabolites of the same classes are reported in Additional file 3, Figures S16 and S17 and are illustrated in Additional file 4: Text S7.

**Table 4:** Top 30 Gaussian Graphical Model (GGM) edge weights

Rank^a^	Metabolite 1	Metabolite 2	Pig PCC	Human PCC^b^	mQTL^c^	Metabolic pathways/reactions
1	SM C18:0	SM C18:1	0.784	0.767^‡^	19–23	One desaturation
2	lysoPC a C16:0	lysoPC a C18:0	0.722	0.731^†^	51, 64	One elongation
3	SM C16:1	SM C18:1	0.689	0.765^†^	19–23	One elongation
4	PC aa C38:6	PC aa C40:6	0.678	0.709^†^	41, 67	One elongation
5	PC aa C38:3	lysoPC a C20:3	0.669	0.5–0.7^‡^	41	Lands cycle
6	Ile	Leu	0.664	0.506^§^	2, 34	Branched-chain amino acids
7	Histamine	Serotonin	0.634	–	69	Co-regulation
8	C10	C8	0.627	0.735^†^	55	β-oxidation step—β-oxidation intermediate
9	lysoPC a C18:2	lysoPC a C20:4	0.622	–	74	One elongation and two desaturations
10	PC ae C40:1	PC ae C42:2	0.599	–	46, 47	One elongation and one desaturation
11	C4:1	H1	0.589	–	–	β-oxidation and energy homeostasis
12	SM (OH) C22:1	SM (OH) C22:2	0.589	≥ 0.7^‡^	–	Sphingolipid-specific desaturation
13	PC aa C40:4	PC aa C40:5	0.573	0.5–0.7^‡^	16, 82	One desaturation
14	C10:1	C12:1	0.569	0.3–0.5^‡^	–	β-oxidation intermediates
15	PC ae C36:1	PC ae C36:2	0.538	–	38, 59	Desaturation
16	ADMA	Total-DMA	0.533	–	32, 56	Arginine *N*-methyltransferases (PRMTs) – total.DMA = ADMA + SDMA
17	SM C16:0	SM C16:1	0.524	0.5–0.7^‡^	–	Sphingolipid-specific desaturation
18	lysoPC a C18:1	lysoPC a C18:2	0.507	0.5–0.7^‡^	–	One desaturation
19	PC aa C36:2	lysoPC a C18:0	0.481	0.3–0.5^‡^	–	Lands cycle
20	PC ae C36:4	PC ae C38:5	0.469	–	52	One desaturation
21	PC aa C38:4	lysoPC a C20:4	0.467	0.3–0.5^‡^	70, 74	Lands cycle
22	SM C24:0	SM (OH) C24:1	0.455	–	–	One hydroxylation and one desaturation
23	PC aa C34:2	PC aa C34:3	0.449	–	–	One desaturation
24	PC aa C36:4	PC aa C36:5	0.444	–	15, 95	One desaturation
25	PC ae C34:2	PC ae C36:3	0.443	–	18	One elongation and one desaturation
26	SM C24:0	SM C24:1	0.440	0.577^†^	–	Sphingolipid-specific desaturation
27	PC aa C36:3	lysoPC a C18:1	0.440	–	–	Lands cycle
28	PC aa C34:2	PC aa C36:4	0.406	–	–	One elongation and two desaturations
29	Lys	Orn	0.405	–	3, 4, 65, 75, 96	Biosynthesis of amino acids
30	PC ae C38:4	PC ae C40:4	0.400	0.3–0.5^‡^	41	One elongation

The reported results are ranked based on the Partial Correlation Coefficient (PCC) obtained from the metabolomic profiles of the investigated Large White and Duroc pigs. The table also includes PCC information reported in previous studies in humans for the same metabolite pairs, the linked mQTL identified in pigs and the proposed metabolic pathway explanations

^a^The complete list of significant PCC is reported in Additional file 1: Table S18

^b^PCC reported for the same pair of metabolites in humans

^c^mQTL number correspondence is reported in Additional file 1: Table S11

^‡^Information from Krumsiek et al. [67]

^§^information from Mittelstrass et al. [55]

^¶^Information from Krumsiek et al. [56]

The top four PCC values were for metabolite pairs (sphingomyelins, lysophosphatidylcholines or phosphatidylcholines) that are separated by just one enzymatic step of desaturation or elongation. The 5th ranked PCC involves metabolites that belong to two subgroups of the glycerophospholipid class (PC aa C38:3and lysoPC a C20:3; PCC = 0.669) and that differ by a C18∶0 fatty acid residue, probably linked by still-uncharacterized step(s) of the Lands cycle. All of these top ranked metabolite pairs have also been reported to have high PCC values in humans [55, 56, 67].

One of the highest PCC values was obtained for the pair histamine-serotonin (PCC = 0.63). Histamine is a biogenic amine, with a variety of important functions, and is mainly synthesized in basophils and mast cells. Serotonin (also known as 5-hydroxytryptamine or 5-HT) is another biogenic amine and a well-known neurotransmitter. Biochemically, histamine and serotonin are not directly linked, so another explanation for this high PCC value should be considered. Histamine can inhibit serotonin release in some neural tissues via histamine H3 receptors (H_3_Rs) [68, 69]. Recently, the relation between these two metabolites has been suggested to be due to a novel mechanism that regulates activity of the serotonin transporter (SERT, also known as SERT1 or SLC6A4) by the H_3_R-mediated CaMKII/calcineurin pathway that controls reuptake and clearance of released serotonin in the central nervous system [70]. It remains to be evaluated whether these regulatory steps can explain the high PCC value observed in pigs. *SLC6A4* is also the candidate gene associated with the level of serotonin reported here for the GWAS for Large White pigs (Table 2).

Another interesting result included, again, serotonin, which had a significant PCC value with taurine (PCC = 0.306) [see Additional file 1: Table S18]. Taurine, one of the most abundant free amino acids in vertebrates, plays various important physiological roles, including in development and neuronal activity. This significant PCC value observed in pigs may be due to an indirect mechanism that involves regulation of the serotonin receptor and its release, as suggested by preliminary results from a zebrafish model, in which taurine-mediated aggression is abolished via serotonin receptor antagonism [71]. The indirect relationships between these three metabolites (serotonin, taurine, and histamine), as identified through the PCC of the latter two with serotonin, could be attributed to some not yet defined feedback regulation systems that involve these three metabolites. It is worth noting that in Large White pigs, the lead SNP near *SLC6A4* for the mQTL for serotonin was also suggestively associated with taurine, further suggesting a potential indirect biological connection between these metabolites (Tables 2 and 4; see Additional file 3: Figure S19).

## Discussion

Our study represents the largest investigation that has merged metabolomics and genomics in pigs to date. By combining information from heritability estimation and GWAS, we report that many basic components of pig metabolism, defined in this study as plasma metabolites from various biochemical classes, are influenced, at least in part, by genetic factors that contribute to modifying their circulating concentration and can, therefore, be indicated as genetically influenced metabolites or GIM. These genetic factors may have cascade effects on several pathways or metabolic steps. This aligns with results reported in humans [7, 9–11, 55–59]. In this context, adding data from another mammalian species, such as the pig, provides comparative information that contributes to uncover the complexity of the mammalian metabolomic landscape. This can lead to the development of new concepts and hypotheses that support the role of various metabolites in fundamental biological processes, which may also be valuable in explaining their significance in human diseases.

Based on our results, we conducted a human-pig comparative analysis of the estimated heritability of various metabolite classes. This analysis utilized information from the review study on humans by Hagenbeek et al. [8], which included studies based on the same targeted metabolomic approach that we employed in pigs. Despite methodological differences in the collection and analysis of data and in the number of individuals investigated, several key points are worth discussing. The levels of essential amino acids had lower heritability estimates than the levels of nonessential amino acids in both humans and pigs, although this difference was not significant in either species. It has been suggested that essential amino acids, which cannot be synthesized directly by the organism, may generally have lower heritability than nonessential amino acids, because the latter can be influenced directly by the metabolism of the organism [72]. However, this hypothesis requires further support from additional studies. From the human-pig comparative analysis that related heritability estimates with the complexity of the chemical structure of the metabolites [see Additional file 4: Text S2], it became evident that a more detailed examination of various groups of biomolecules is necessary to draw any conclusions. This analysis should consider the number of biochemical steps needed to metabolize these molecules, as well as the major mQTL that have been identified in both species.

Summarizing the results obtained from the two divergent Western pig breeds analyzed here (Large White and Duroc), we identified genome-wide associations for a total of 126 metabolites from 5 analyte classes. These results pointed to a total of 97 mQTL regions, distributed across all porcine autosomes. It is also worth mentioning that the high level of LD presents in pig populations [34, 35] can make it difficult to evaluate if the associations are due to one or more loci, especially for the most extended regions. Further studies would be needed to analyze some of the mQTL regions. Of the 97 mQTL regions identified in this study, 29 were only found in Large White pigs, and 33 were only found in Duroc pigs. The remaining mQTL were discovered in both breeds (n = 5), in one breed or the other and in meta-analyses (15 and 5, respectively, also identified in either the Large White or the Duroc breed), or solely in meta-analyses (n = 10). Some of the mQTL that emerged from meta-analyses showed clear segregation in the two breeds when breed-specific two-dimensional pictures based on metabolite ratios were included. Other mQTL that were identified with meta-analyses could result from the combination of different breed-specific effects of the same or closely linked loci, as the two-dimensional analyses produced from the metabolite showed different patterns in the two breeds, in particular for mQTL for which more than one candidate gene was highlighted (i.e. Fig. 4c–e). We therefore demonstrated for the first time in this study that ratio-based analyses can be useful in extracting additional information for interpreting and defining the role of certain mQTL, and can contribute to establishing correspondence between different populations.

Summarizing these results, we can clearly state that the mQTL patterns in Large White and Duroc breeds are largely different. When the mQTL segregate in both breeds, allele frequencies of the lead SNP are usually opposite in the two breeds [see Additional file 1: Tables S9; Additional file 2: Table S15], further supporting various metabolic differences between Large White and Duroc pigs. In many pig production systems, these two breeds are utilized to form lines that are then crossed to exploit heterosis, which can be the result of, at least in part, the combination of different genetic factors that are involved in basic metabolic processes. It was interesting to note that the number of breed specific mQTL was similar for the two breeds, despite the different number of animals investigated in the Large White and Duroc breeds. In Duroc pigs, the use of metabolite ratios was very effective in revealing novel (and independent) breed specific mQTL that did not emerge in the single metabolite analysis (15 out of 33 mQTL were Duroc specific). Although the number of significant metabolite ratios was largest for Large White pigs (mainly involving phosphatidylcholines), most of them identified the same few major mQTL already identified in single metabolite GWAS. The utility of metabolite ratios has already been demonstrated in human GWAS to reveal several mQTL that could not be identified by using single metabolite information [7, 53, 73]. This suggests that ratios can contribute to identifying genetic determinants that affect specific reaction steps or groups of metabolites within the same metabolic pathways or that may be co-regulated.

For 66% of the 97 mQTL regions, one or more potential effector genes could be identified. Many of these genes can be considered obvious candidates based on already well-established information, due to their direct involvement in the metabolic steps and biological mechanisms that include the corresponding metabolites. The identification of strong candidate genes was also possible within the Duroc breed, in which GWAS were based on a relatively low number of pigs. This suggests that the use of molecular phenotypes (i.e. the metabotypes) that are closely linked to genetic variation is able to provide a snapshot of the genetic determinants that affect metabolism in relatively small experimental designs.

We then obtained comparative human-pig information for the identified mQTL, providing inter-species confirmation of candidate genes and opening new windows on the biological roles of the encoded enzymes or transporters. For example, considering the findings in pigs, it would be intriguing to investigate further if altered lysophosphatidylcholine accumulation [63, 64] could be the molecular mechanism that links *MFSD8* variants to the predisposition to neuronal ceroid lipofuscinoses in humans.

When we attempted to assess whether causative mutations of the identified mQTL would be determined by variants that affect the encoded protein structures based on concordant evidence, including the predicted effects of the variants, segregation in the breed where the mQTL was identified, and LD with the lead SNP, we could only assert this in a very few cases. Therefore, we can hypothesize that most of the mQTL are better explained by regulatory variants that may alter the expression of the identified genes, which aligns with what has already been suggested in humans [53]. Therefore, complementing genotype-tissue expression datasets in pigs [74] with metabolomic data can provide additional information to elucidate the biological diversity in this livestock species, ultimately leading to a better understanding of basic biological processes.

We then further investigated one mQTL, due to its relevance in affecting a key component of the kynurenine pathway, which is linked to tryptophan catabolism, an essential amino acid that is a limiting factor in pig nutrition. Although additional functional studies are needed, we suggest that the *KMO* haplotypes that produce two different protein forms (distinguished by a few residues, including an insertion/deletion on an additional residue) can be the genetic determinants for the different levels of plasma kynurenine. The two haplotypes are not differentially expressed, and the two derived protein forms are not differentially abundant in the liver, which is one of the most important tissues where KMO works. We then designed a nutrigenetic study in which we fed piglets with opposite *KMO* genotypes varying levels of tryptophan. By evaluating the cascade effects on other metabolites in the kynurenine pathway, we were able to model the KMO reaction kinetics. Further studies are needed to evaluate how pigs with different *KMO* genotypes respond in terms of growth performances and amino acid uptake when fed different levels of tryptophan.

Thus, studies in pigs focused on the nutritional need for tryptophan should also consider the *KMO* genotypes of the animals. Since our results indicate that the levels of several other amino acids are associated with genes encoding specific enzymes involved in their metabolism, additional nutrigenetic studies can be developed based on the information obtained from the GWAS in the two breeds. Therefore, these results, focusing on important elements in pig nutrition, can open new research avenues that integrate pig nutrition and genetics, paving the way for the development of innovative concepts in precision feeding.

The metabolomic profiles we obtained in pigs were also used to reconstruct metabolic relationships using a GGM and to evaluate the impact of specific mQTL on their networks. The correlations observed in the metabolomic data confirm direct relationships between many metabolites, separated by one or a few reaction steps. Based on these expected results that indirectly confirm the ability of GGM to capture metabolic pathway information, the high PCC values that we identified for several non-obvious pairs of metabolites create opportunities for further investigations to clarify potential novel metabolic relationships. Of note, the triangulation between serotonin, taurine, and histamine that we observed in their respective pairwise correlations can be useful in clarifying their interplay and evaluating their involvement in explaining the genetic components of behavioral traits in pigs. This information may be highly relevant in pig breeding to improve animal welfare.

Our study has several limitations that must be pointed out. Metabolomics profiles of the animals used for all genetic analyses were obtained at the end of the production life of the sib-tested pigs, at 9 months of age, after slaughter. Therefore, this final time point, which also includes stressful conditions for the pigs, such as movement, loading, transportation, and then slaughtering procedures, may have altered the basal level of several metabolites. This could also have affected the potential effects of some genetic factors on metabolite levels. On the other hand, all of these factors, including feeding and pre-slaughter feed deprivation of the animals, were applied in a consistent and controlled manner to all animals. This eliminated some of the confounding elements that typically reduce the power of experimental designs in metabolomics studies involving humans. Nevertheless, the metabolomic profiles of other developmental and growth phases of the pigs were not explored. Conducting more comprehensive measurements of the pig metabolome, which would involve additional animals, different breeds, and other metabolomic approaches that are capable of detecting more metabolites will provide further insight into the genetic factors influencing the metabolism in this livestock species.

## Conclusions

This study has provided the first catalog of genetic factors affecting the pig blood metabolome. This information allowed for comparisons with what is already known in humans. The findings help in understanding the genetic regulation of metabolism in pigs, provide several hypothesis-generating elements, and strengthen the relevance of the pig as biomedical model. The identification of many mQTL in pigs that highlighted gene-metabolite associations showcases the usefulness of merging metabolomics and genomics to identify genetically influenced metabolites and use metabotypes to help dissect several production traits and link genetics and nutrition in this species. Other nutrigenetic experiments can be developed based on genetically influenced metabolites that also constitute important nutrients, paving the way for a systematic exploration of the results obtained in this study and ultimately designing novel nutrition strategies in pigs. Further studies will be designed considering genetically influenced metabolites as molecular phenotypes useful for predicting production traits with the final aim of integrating them into novel breeding and selection programs in pigs.

## Supplementary Information

Additional file 1: Table S1. Metabolites included in the study. Summary statistics of metabolite levels for the Large White and Duroc pig populations are provided. Table S2. List of the components of the basic diet for the piglets involved in the nutrigenetic longitudinal study. Table S3. Analyzed composition of the basic diet for the piglets involved in the nutrigenetic longitudinal study. Table S8. Narrow sense heritability (h^2^_P_) and genomic heritability (h^2^_SNP_) of the metabolite level. Description: Estimates are provided for the Large White and Duroc pig populations. Negative estimates are not reported. Table S9. All genetic association signals obtained in the Large White and Duroc pig populations. Description: Results include the study of both single metabolites and metabolite ratios. Table S10. All genetic association signals obtained in the meta-analyses. Description: Results include the study of both single metabolites and metabolite ratios. Table S11. Complete list of mQTL identified in pigs and comparative analysis of mQTL between pigs and humans. Table S12. Extended information obtained from GWAS Catalog of mQTL identified in humans. Table S14. Genetic variants identified from whole-genome resequencing of Large White, Duroc and Landrace pigs. Description: Only missense variants, frameshift variants, in-frame deletions and insertions, stop gained and start lost variants are reported. For each pig breed (Large White, Duroc and Landrace), the number of animals carrying the three genotypes and allele frequencies are provided. Table S18. All significant Gaussian Graphical Model (GGM) edge weights (i.e. Partial Correlation Coefficients, PCC > 0.3) obtained from the metabolomic profiles of the investigated Large White and Duroc pigs. Description: Comparative information reported in previous studies in humans, the linked mQTL and the proposed metabolic pathway explanations are provided.

Additional file 2: Table S4. List of metabolites in the kynurenine pathway (KP) analyzed in the pigs included in the nutrigenetic study and statistics of metabolites stratified by genotype and metabolic condition at the basal and after the tryptophan (Trp) supplementation. Description: Metabolomic data are obtained from the Bevital platform on plasma of Large White × Landrace piglets. Table S5. Systems of ordinary differential equations (ODE) used to model the kynurenine pathway. Description: Metabolites are those reported in Additional file 2: Table S4. Table S6. Information used in the kinetic modeling of the kynurenine pathway. Table S7. Human kinetic constants. Table S13. Distribution of the predicted consequences for variants identified from whole-genome resequencing of Large White, Duroc and Landrace pigs. Table S15. Variants altering the protein coding sequence of the *KMO* gene. Description: Variant constituting two major haplotypes are those and forming two major haplotypes. Table S16. Allele frequency of the *KMO* polymorphisms identified in several pig breeds and in wild boars. Table S17. Partial Correlation Coefficients (PCC) in Large White pigs before and after the inclusion of the genetic effect from the mQTL (genotype-corrected PCC) in the GGM construction. Description: Underlined, the PPC >0.3.

Additional file 3: Figure S1. Simplified representation of the kynurenine pathway (KP), with information used in the kinetic modelling. Description: Metabolites are shown in boxes and reactions directions are shown by arrows labelled with the acronym of the enzymes. Abbreviations for the metabolites: Trp, tryptophan; KYN, kynurenine; XA, xanthurenic acid; AA, anthranilic acid; KA, kynurenic acid; HK, 3-hydroxykynurenine; HAA, 3-hydroxyanthranilic acid; QUIN, quinolinic acid. Abbreviations for the enzymes: TDO, tryptophan 2,3-dioxygenase; KYNU1, kynureninase; KYNU2, kynureninase; KMO, kynurenine 3-monoxygenase; KAT1, kynurenine aminotransferase; KAT2, kynurenine aminotransferase; 3HAO, 3-hydroxyanthranilate 3,4-dioxygenase. Figure S2. Relationship between heritability and number of carbon atoms (with only one double bound) present in acylcarnitines, glycerophospholipids and sphingomyelins. Figure S3. Relationship between heritability and number of carbon atoms or double bounds within the phosphatidylcholine group. Description: a) Phosphatidylcholine acyl-alkyls with three double bounds (PC ae CX:3). b) Phosphatidylcholines acyl-alkyls with 36 carbon atoms and 1 to 5 double bonds (PC ae C36:X, X=1,..,5). c) Phosphatidylcholines acyl-alkyls with 38 carbon atoms and 1 to 6 double bonds (PC ae C38:X, X=1,..,6). Figure S4. Complete profiles of associations of the mQTL (with *FADS6* as candidate gene) over the metabolite pairs (ratios) of lysophosphatidylcholines and phosphatidylcholines. Description: The -log10(P) is reported. Figure S5. Complete profiles of associations of the mQTL (with *PLIN1* as candidate gene) over metabolite pairs (ratios) of lysophosphatidylcholines and phosphatidylcholines. Description: The -log10(P) is reported. Figure S6. Complete profiles of associations of the mQTL (with *FADS1/FADS2/FADS3* as candidate genes) over metabolite pairs (ratios) of lysophosphatidylcholines and phosphatidylcholines. Description: The -log10(P) is reported. Figure S7. Complete profiles of associations of the mQTL (with *FAR1* as candidate gene) over metabolite pairs (ratios) of lysophosphatidylcholines and phosphatidylcholines. Description: The -log10(P) is reported. Figure S8. Complete profiles of associations of the mQTL (with *LPCAT2/SMPD3* as candidate genes) over metabolite pairs (ratios) of lysophosphatidylcholines and phosphatidylcholines. Description: The -log_10_(*P*) is reported. Figure S9. Plasma concentration of metabolites of the kynurenine pathway (KP) in Large White × Landrace piglets homozygous for the alternative *KMO* haplotypes (indicated with the tag SNP: rs81278711-AA and rs81278711-GG) at the basal tryptophan level (no tryptophan supplementation). Description: Abbreviations: Trp, tryptophan; KYN, kynurenine; XA, xanthurenic acid; AA, anthranilic acid; KA, kynurenic acid; HK, 3-hydroxykynurenine; HAA, 3-hydroxyanthranilic acid; QUIN, quinolinic acid. Figure S10. Plasma concentration of metabolites of the kynurenine pathway (KP) in Large White × Landrace piglets homozygous for the alternative *KMO* haplotypes (indicated with the tag SNP: rs81278711-AA and rs81278711-GG) after tryptophan supplementation. Description: Abbreviations: Trp, tryptophan; KYN, kynurenine; XA, xanthurenic acid; AA, anthranilic acid; KA, kynurenic acid; HK, 3-hydroxykynurenine; HAA, 3-hydroxyanthranilic acid; QUIN, quinolinic acid. Figure S11. Relationships between kynurenine (KYN) levels and its first neighbor metabolites as stratified for the alternative *KMO* haplotypes (indicated with the tag SNP: rs81278711-AA and rs81278711-GG). Description: Data are from the nutrigenetic study in the Large White × Landrace piglets, after tryptophan supplementation. a) Haplotypes differ in [KYN] but not in [Trp]. b) Haplotypes differ in [KYN] but not in [HK]. c) Haplotypes differ in [KYN] and in [KA]. d) Haplotypes differ in [KYN] and in [AA]. Abbreviations: Trp, tryptophan; KYN, kynurenine; AA, anthranilic acid; KA, kynurenic acid; HK, 3-hydroxykynurenine. Figure S12. Results of gene expression and Western blotting analyses for KMO. Description: Liver samples of pigs carrying two different *KMO* genotypes (rs81278711-AA and rs81278711-GG) were analysed. A) Results of the qPCR analyses including average Ct values for *KMO* and *B2M* genes obtained for the pigs with different *KMO* genotypes. The relative gene expression was obtained as 2−∆∆Ct and presented as averaged measures, considering all animals with the same genotype (as no gene expression differences were observed between the two genotypes in both experimental designs, namely the performance tested Large White gilts and the crossbred pigs of the nutrigenetic experiment; t-test, P = 0.65). B) Examples of Western blot images from 6 pigs (pigs 1, 2 and 3 with genotype rs81278711-AA; pigs 4, 5 and 6 with genotype rs81278711-GG) and the corresponding band lines stained with Coomassie Brilliant Blue used to normalize the samples. The statistical analysis showed no significant band differences between the two *KMO* genotypes (P = 0.202). M: Molecular weight marker. Figure S13. Domains of the pig KMO protein. Description: Domains are annotated with Interpro tool on the protein KMO_PIG (Q9MZS9). FAD-binding domain is detected by the PFAM entry PF01494 (FAD_binding_3), a member of the clan NADP_Rossmann. Positions corresponding to the differences in the two haplotypes are indicated. The variations S95F, Q135R and V178L occur in the FAD binding domain and may influence the cofactor binding. Figure S14. Inclusion of the mQTL information in GGM and correlation analyses. Description: a) Partial Correlation Coefficients (PCC) *vs*. genotype-corrected PCC. b) Pearson’s correlations (r) *vs*. genotype-corrected Pearson’s correlations. Figure S15. Inclusion of the effect of the *SMPD3/LPCAT2* genotypes in the estimation of metabolite correlations. Description: a) Effect of the *SMPD3/LPCAT2* genotypes on PC aa C36:4 and PC aa C36:1 metabolites (different direction of the β of association). b) Relationship between PC aa C36:4 and PC aa C36:1 metabolites. c) Effect of the *SMPD3/LPCAT2* genotypes on PC aa C36:4 and PC aa C36:1 metabolites after inclusion of genetic information. d) Relationship between on PC aa C36:4 and PC aa C36:1 metabolites after inclusion of genetic information. In b) and d), different colors represent the pigs with different *SMPD3/LPCAT2* genotypes. Figure S16. Inclusion of the effect of the *SLC6A4* genotypes in the estimation of metabolite correlations. Description: a) Effect of the *SLC6A4* genotypes on serotonin and taurine metabolites (same direction of the β of association). b) Relationship between serotonin and taurine metabolites. c) Effect of the *SLC6A4* genotypes on serotonin and taurine metabolites after inclusion of genetic information. d) Relationship between serotonin and taurine metabolites after inclusion of genetic information. In b) and d), different colors represent the pigs with different *SLC6A4* genotypes. Figure S17. Comparative analysis of Partial Correlation Coefficients (PCC) and Pearson’s correlation coefficients between a few sphingomyelins and information on the associated genes as reported in the GWAS. Description: Results are from the Large White population. a) Partial correlation coefficients (|PCC|≥ 0.3). b) Correlation coefficients (|r|≥ 0.3). c) mQTL associated with single metabolites. Gray edges represent correlation coefficients. d) mQTL associated with metabolite ratios (arrows indicates affected metabolite pairs). Gray edges represent correlation coefficients. Information on the relevant mQTL number is reported in Additional file 1: Table S11. Figure S18. Comparative analysis of partial correlation coefficients (PCC) and Pearson’s correlation coefficients between a few lysophosphatidylcholines and information on the associated genes as reported in the GWAS. Description: Results are from the Large White population. Gray edges represent correlation coefficients. Metabolites and ratios targeted by mQTL 41 (*LPCAT2/SMPD3*) are highlighted by red and blue arrows, respectively. Information on the relevant mQTL number is reported in Additional file 1, Table S11. Figure S19. Manhattan plot results for serotonin and taurine GWAS in Large White pigs. Description: Green dots represent suggestively and significantly associated SNPs (P<5.0×10^-5^).

Additional file 4: Text S1. Detail on quantitative real time PCR and western blotting analyses of KMO. Text S2. Relationships between heritability estimates of some metabolites in pigs and their chemical structures. Text S3. Comparative information on candidate genes between humans and pigs. Text S4. Identification and annotation of variants from whole genome resequencing data. Text S5. Evaluation of KMO mutations in relation to its protein sequence and structure. Text S6. Inclusion of the genetic effect in the Gaussian Graphical Model (GGM) construction. Text S7. Merging GGM and GWAS results.

## Data Availability

Summary raw data on the concentration of the analyzed metabolites in Large White and Duroc pigs are available in Zenodo: 10.5281/zenodo.14046073. Other datasets used and/or analyzed during the current study are available from the corresponding author on reasonable request.
