# Supplementary material for: Merging metabolomics and genomics provides a catalog of genetic factors that influence molecular phenotypes in pigs linking relevant metabolic pathways
Source: Genet Sel Evol. 2025 Mar 6;57:11. doi: 10.1186/s12711-025-00960-8 (PMC11887101; doi:10.1186/s12711-025-00960-8)
Supplement: Supplementary file 2 — Additional file 2: Table S4. List of metabolites in the kynurenine pathway (KP) analyzed in the pigs included in the nutrigenetic study and statistics of metabolites stratified by genotype and metabolic condition at the basal and after the tryptophan (Trp) supplementation. Description: Metabolomic data are obtained from the Bevital platform on plasma of Large White × Landrace piglets. Table S5. Systems of ordinary differential equations (ODE) used to model the kynurenine pathway. Description: Metabolites are those reported in Additional file 2: Table S4. Table S6. Information used in the kinetic modeling of the kynurenine pathway. Table S7. Human kinetic constants. Table S13. Distribution of the predicted consequences for variants identified from whole-genome resequencing of Large White, Duroc and Landrace pigs. Table S15. Variants altering the protein coding sequence of the KMO gene. Description: Variant constituting two major haplotypes are those and forming two major haplotypes. Table S16. Allele frequency of the KMO polymorphisms identified in several pig breeds and in wild boars. Table S17. Partial Correlation Coefficients (PCC) in Large White pigs before and after the inclusion of the genetic effect from the mQTL (genotype-corrected PCC) in the GGM construction. Description: Underlined, the PPC >0.3. [file 12711_2025_960_MOESM2_ESM.docx]

Additional file 2

**Merging metabolomics and genomics provides a catalog of genetic factors that influence molecular phenotypes in pigs linking relevant metabolic pathways**

Samuele Bovo^1*^, Anisa Ribani^1^, Flaminia Fanelli^2,3^, Giuliano Galimberti^4^, Pier Luigi Martelli^5^, Paolo Trevisi^6^, Francesca Bertolini^1^, Matteo Bolner^1^, Rita Casadio^5^, Stefania Dall’Olio^1^, Maurizio Gallo^7^, Diana Luise^6^, Gianluca Mazzoni^1^, Giuseppina Schiavo^1^, Valeria Taurisano^1^, Paolo Zambonelli^1^, Paolo Bosi^6^, Uberto Pagotto^2,3^, Luca Fontanesi^1*^

^1^Animal and Food Genomics Group, Division of Animal Sciences, Department of Agricultural and Food Sciences, University of Bologna, Bologna, Italy

^2^Endocrinology Research Group, Center for Applied Biomedical Research, Department of Medical and Surgical Sciences, University of Bologna, Bologna, Italy

^3^Division of Endocrinology and Prevention and Care of Diabetes, IRCCS Azienda Ospedaliero-Universitaria di Bologna, Policlinico di Sant’Orsola, Bologna, Italy

^4^Department of Statistical Sciences “Paolo Fortunati”, University of Bologna, Bologna, Italy

^5^Biocomputing Group, Department of Pharmacology and Biotechnology, University of Bologna, Bologna, Italy

^6^Laboratory on Animal Nutrition and Feeding for Livestock Sustainability and Resilience, Division of Animal Sciences, Department of Agricultural and Food Sciences, University of Bologna, Bologna, Italy

^7^Associazione Nazionale Allevatori Suini, Roma, Italy

*Corresponding authors: Samuele Bovo (SB); Luca Fontanesi (LF).

E-mail addresses: [samuele.bovo@unibo.it](mailto:samuele.bovo@unibo.it); [luca.fontanesi@unibo.it](mailto:luca.fontanesi@unibo.it)

**Table S2.** **List of the components of the basic diet for the piglets involved in the nutrigenetic longitudinal study**.

| **Components** | **%, as-fed basis** |
| --- | --- |
| Corn | 25.49 |
| Barley | 23.70 |
| Soybean meal, 50 % crude protein | 21.50 |
| Whey, acid, skimmed, dehydrated | 11.85 |
| Wheat, soft | 7.00 |
| Soybean oil | 3.50 |
| Potato, protein concentrate | 3.50 |
| Dicalcium phosphate hydrated | 1.30 |
| Vitamin and trace mineral mixture^a^ | 0.60 |
| L-Lysine HCl | 0.57 |
| Calcium carbonate | 0.50 |
| DL-Methionine | 0.22 |
| L-Threonine | 0.21 |
| L-Tryptophan | 0.03 |
| L-Valine | 0.03 |

^a^ Provided per 1 kg of feed: vitamin A, 6,500 IU; vitamin D3, 2,000 IU; vitamin E , 50 mg; choline, 250 mg; vitamin B1, 2 mg; vitamin B2, 4 mg; vitamin B6, 3 mg; vitamin B12, 0.025 mg; vitamin K, 2.5 mg; niacin, 25 mg; pantothenic acid, 12.5 mg; biotin, 0.25 mg; copper, 20 mg; selenium, 0.25 mg; zinc, 125 mg; iron, 150 mg; manganese, 100 mg; iodine, 1.5 mg; phytase, 500 FTU.

**Table S3.** **Analyzed composition of the basic diet for the piglets involved in the nutrigenetic longitudinal study.**

| **Item** | **%, as-fed basis** |
| --- | --- |
| Dry Matter | 90.8 |
| Crude protein - TN x 6.25 | 19.0 |
| Lysine | 1.38 |
| Threonine | 0.91 |
| Methionine | 0.483 |
| Cystine + Cystein | 0.303 |
| Methionine + Cystine | 0.786 |
| Tryptophan | 0.236 |
| Valine | 0.88 |
| Isoleucine | 0.77 |
| Leucine | 1.42 |
| Arginine | 1.06 |
| Phenylalanine | 0.88 |
| Tyrosine | 0.64 |
| Histidine | 0.43 |
| Serine | 0.85 |
| Alanine | 0.81 |
| Aspartic Acid | 1.69 |
| Glutamic Acid | 3.13 |
| Glycine | 0.75 |
| Proline | 1.15 |

**Table S4.** **List of metabolites in the kynurenine pathway (KP) analyzed in the pigs included in the nutrigenetic study and statistics of metabolites stratified by genotype and metabolic condition at the basal and after the tryptophan (Trp) supplementation.** Metabolomic data are obtained from the Bevital platform on plasma of Large White × Landrace piglets.

|  | **Trp basal** | | | | | **After Trp supplementation** | | | | |
| --- | --- | --- | --- | --- | --- | --- | --- | --- | --- | --- |
|  | ***KMO*^rs81278711-AA^** | | ***KMO*^rs81278711-GG^** | |  | ***KMO*^rs81278711-AA^** | | ***KMO*^rs81278711-GG^** | |  |
| **Metabolite^a^** | **Mean** | **SD** | **Mean** | **SD** | ***P*^b^** | **Mean** | **SD** | **Mean** | **SD** | ***P*^c^** |
| Trp | 3.97E+01 | 9.62E+00 | 4.58E+01 | 1.65E+01 | 7.21E-01 | 7.42E+01 | 1.42E+01 | 7.79E+01 | 2.05E+01 | 4.42E-01 |
| KYN | 1.11E+00 | 2.27E-01 | 7.16E-01 | 2.26E-01 | 1.86E-03 | 3.33E+00 | 1.11E+00 | 1.46E+00 | 4.31E-01 | 6.22E-04 |
| HK | 5.45E+01 | 1.86E+01 | 5.89E+01 | 1.12E+01 | 4.29E-01 | 1.39E+02 | 5.37E+01 | 1.60E+02 | 3.75E+01 | 3.83E-01 |
| KA | 8.97E+00 | 2.78E+00 | 6.15E+00 | 2.30E+00 | 3.79E-02 | 2.81E+01 | 1.35E+01 | 1.06E+01 | 3.41E+00 | 1.86E-03 |
| XA | 1.86E+00 | 7.89E-01 | 1.96E+00 | 1.14E+00 | 7.98E-01 | 4.90E+00 | 2.45E+00 | 4.41E+00 | 2.25E+00 | 7.21E-01 |
| AA | 3.23E+01 | 7.57E+00 | 3.27E+01 | 1.27E+01 | 7.92E-01 | 7.80E+01 | 1.62E+01 | 4.92E+01 | 8.96E+00 | 4.08E-03 |
| HAA | 4.93E+01 | 1.22E+01 | 5.46E+01 | 1.31E+01 | 4.29E-01 | 1.21E+02 | 3.18E+01 | 1.04E+02 | 2.30E+01 | 3.18E-01 |
| QUIN | 5.38E+03 | 1.04E+03 | 4.79E+03 | 1.04E+03 | 2.48E-01 | 6.79E+03 | 7.12E+02 | 6.38E+03 | 6.55E+02 | 4.42E-01 |

^a^ Metabolites: Trp, tryptophan (µmol/L); KYN, kynurenine (µmol/L); HK, 3-hydroxykynurenine (nmol/L); KA, kynurenic acid (nmol/L); XA, xanthurenic acid (nmol/L); AA, anthranilic acid (nmol/L); HAA, 3-hydroxyanthranilate (nmol/L); QUIN, quinolinic acid (nmol/L). ^b^ *P*-value of Wilcoxon Rank Sum Test within the “Trp basal” condition. ^c^ *P*-value of Wilcoxon Rank Sum Test within the “Trp Supplementation” condition.

**Table S5.** **Systems of ordinary differential equations (ODE) used to model the kynurenine pathway**. Metabolites are those reported in Additional file 2, Table S4.

| **Equation number** | **Equation** |
| --- | --- |
| Equation S1 | $\frac{d[KYN]}{dt}= k_{TDO/IDO} \cdot\left[ \mathrm{Trp} \right] - k_{KYNU1} \cdot\left[ \mathrm{KYN} \right] - k_{KMO} \cdot\left[ \mathrm{KYN} \right] - k_{KAT1} \cdot\left[ \mathrm{KYN} \right]$ |
| Equation S2 | $\frac{d[AA]}{dt}= k_{KYNU1} \cdot\left[ \mathrm{KYN} \right] - k_{i} \cdot\left[ \mathrm{AA} \right]$ |
| Equation S3 | $\frac{d[HK]}{dt}= k_{KMO} \cdot\left[ \mathrm{KYN} \right] - \left[ \mathrm{HK} \right]\cdot(k_{KYNU1}+k_{KYNU2})$ |
| Equation S4 | $\frac{d[HAA]}{dt}=k_{KYNU2} \cdot\left[ HK \right] - k_{3HAO} \cdot[HAA]$ |
| Equation S5 | $\frac{\text{d[KA]}}{\text{dt}}\text{= }\text{k}_{\text{KAT1}}\text{ · }\left[ \text{KYN} \right]\text{ – }\text{k}_{\text{x}}\text{ · }\left[ \text{KA} \right]$ |
| Equation S6 | $\frac{d[XA]}{dt}= k_{KAT2} \cdot\left[ \mathrm{HK} \right] - k_{y} \cdot\left[ \mathrm{XA} \right]$ |
| Equation S7 | $\frac{d[QUIN]}{dt}= k_{3HAO} \cdot\left[ \mathrm{HAA} \right] - k_{z} \cdot\left[ \mathrm{QUIN} \right]$ |

**Table S6.** **Information used in the kinetic modeling of the kynurenine pathway.**

| **Metabolite^a^** | **Behavior^b^** | **Steady states metabolite levels^c^** | **Hypothesis^d^** |
| --- | --- | --- | --- |
| KYN | ↓ | $\left[ {KYN}^{ss} \right]=\frac{k_{TDO/IDO}}{k_{KYNU1}+ k_{\mathrm{KMO}}+ k_{KAT1}}\cdot[Trp$] | [KYN^ss^] decrease because k_TDO/IDO_ decreases or because *k*_KYNU1_ and/or *k*_KMO_ and/or *k*_KAT_ increase. |
| AA | ↓ | $\left[ {AA}^{ss} \right]=\frac{k_{KYNU1}}{k_{i}}\cdot[{KYN}^{ss}$] | [AA^ss^] follows the behavior of [KYN^ss^] if *k*_KYNU1_ and *k*_i_ do not change (or if their ratio is maintained). |
| KA | ↓ | $\left[ {KA}^{ss} \right]=\frac{k_{KAT1}}{k_{x}}\cdot[{KYN}^{ss}$] | [KA^ss^] follows the behavior of [KYN^ss^] if *k*_KAT1_ and *k*_x_ do not change (or if their ratio is maintained). |
| HK | = | $\left[ {HK}^{ss} \right]=\frac{k_{\mathrm{KMO}}}{k_{KYNU2}+k_{KAT2}}\cdot[{KYN}^{ss}]$ | Equal levels of [HK^ss^] are maintained, irrespectively of the concentration of [KYN^ss^], whether *k*_KMO_ increases or *k*_KYNU2_ + *k*_KAT2_ decreases. |
| HAA | = | $\left[ {HAA}^{ss} \right]=\frac{k_{TKYNU1}}{k_{3HAO}}\cdot[{HK}^{ss}]$ | [HAA^ss^] follows the behavior of [HK^ss^] if *k*_KYNU1_ and *k*_3HAO_ do not change (or if their ratio is maintained). |
| XA | = | $\left[ {XA}^{ss} \right]=\frac{k_{KAT2}}{k_{y}}\cdot[{HK}^{ss}$] | [XA^ss^] follows the behavior of [HK^ss^] if *k*_KAT2_ and *k*_y_ do not change (or if their ratio is maintained). |
| QUIN | = | $\left[ {QUIN}^{ss} \right]=\frac{k_{3HAO}}{k_{z}}\cdot[{HK}^{ss}$] | [QUIN^ss^] follows the behavior of [HAA^ss^] if *k*_3HAO_ and *k*_z_ do not change (or if their ratio is maintained). |

^a^Abbreviations: KYN, kynurenine; AA, anthranilic acid; KA, kynurenic acid; HK, 3-hydroxykynurenine; HAA, 3- hydroxyanthranilic acid; XA, xanthurenic acid; QUIN, quinolinic acid. ^b^ The symbol “=” indicates an equal level of the metabolite between the two piglet groups; the symbol “↓” indicates the decrease in metabolite concentration in the KMO^rs81278711-GG^ genotype in comparison with the KMO^rs81278711-AA^ genotype. ^c^ Steady state (ss) metabolite levels derived from the mathematical modelling of the kynurenine pathway (Figure 4a; Additional file 3, Figure S1 and Additional file 2, Table S5). ^d^ Hypothesis explaining the behavior of the metabolite based on the analysis at the steady state (ss).

**Table S7. Human kinetic constants.**

| **Enzyme^a^** | ***K*_M_ (mM)** | ***k*_cat_ (s^-1^)** | ***k*_cat_ /*K*_M_** |
| --- | --- | --- | --- |
| TDO | 0.222 | 1.4 | 6.31 |
| KMO | 0.1 | 2.2 | 22.00 |
| KYNU1 | 0.495 | 0.23 | 0.46 |
| KAT1 | 4.7 | 9.76 | 2.08 |
| KAT2 | 3.8 | 1.7 | 0.45 |
| KYNU2 | 0.028 | 3.5 | 125.00 |
| 3HAO | 0.016 | 64 | 4,000 |

^a^ Enzyme: TDO, tryptophan 2,3-dioxygenase; KMO, kynurenine 3-monoxygenase; KYNU1, kynureninase; KYNU2, kynureninase; KAT1, kynurenine aminotransferase; KAT2, kynurenine aminotransferase; 3HAO, 3-hydroxyanthranilate 3,4-dioxygenase.

**Table S13. Distribution of the predicted consequences for variants identified from whole-genome resequencing of Large White, Duroc and Landrace pigs.**

| **Consequence type** | **Count** |
| --- | --- |
| Intron variant | 2,596,807 |
| Intergenic variant | 542,960 |
| Downstream gene variant | 293,323 |
| Upstream gene variant | 288,293 |
| Non coding transcript variant | 141,485 |
| 3 prime UTR variant | 52,125 |
| Non coding transcript exon variant | 26,192 |
| Synonymous variant | 21,653 |
| 5 prime UTR variant | 15,407 |
| Missense variant | 14,569 |
| Splice region variant | 6,066 |
| Frameshift variant | 1,258 |
| Stop gained | 201 |
| In-frame deletion | 196 |
| In-frame insertion | 137 |
| Coding sequence variant | 62 |
| Start lost | 42 |
| Stop retained variant | 15 |
| Protein altering variant | 8 |
| Mature miRNA variant | 3 |
| Total^a^ | 4,000,802 |

^a^ VEP took as input 1,420,757 variants and gave as output a total of 4,000,802 annotations because co-location and multiple effects of variants.

**Table S15.** **Variants altering the protein coding sequence of the *KMO* gene.** Variant constituting two major haplotypes are those and forming two major haplotypes.

|  |  |  |  |  |  |  |  | **N. of pigs with different genotypes^g^** | | | **LD (*r*^2^)^h^** | | |
| --- | --- | --- | --- | --- | --- | --- | --- | --- | --- | --- | --- | --- | --- |
| **SSC^a^** | **Position^b^** | **Ref/Alt^c^** | **Consequence^d^** | **Protein position^e^** | **SAP^f^** | **rs number** | **SIFT prediction** | **Large White** | **Duroc** | **Landrace** | **Large White** | **Duroc** | **Landrace** |
| 10 | 12458867 | C/T | missense | 95 | S/F | rs321352761 | tolerated | 87/1/0 | 29/6/0 | 35/0/0 | 0.026 | 0.013 | - |
| 10 | 12470256 | A/G | missense | 135 | Q/R | rs332872547 | tolerated | 68/18/2 | 4/15/16 | 16/18/1 | 0.953 | 0.691 | 1.000 |
| 10 | 12471630 | G/T | missense | 178 | V/L | rs320554692 | tolerated | 68/18/2 | 4/15/16 | 16/18/1 | 0.953 | 0.691 | 1.000 |
| 10 | 12486397 | A/G | missense | 347 | K/R | rs333269485 | tolerated | 67/19/2 | 2/13/20 | 16/18/1 | 1.000 | 1.000 | 1.000 |
| 10 | 12488061 | A/G | missense | 388 | T/A | rs81216579 | tolerated | 67/19/2 | 2/13/20 | 16/18/1 | 1.000 | 1.000 | 1.000 |
| 10 | 12488223 | A/G | missense | 412 | M/V | rs327523644 | tolerated | 67/19/2 | 0/11/24 | 16/18/1 | 1.000 | 0.610 | 1.000 |
| 10 | 12489135 | A/ACCT | in-frame insertion | 439 | Y/YL | - | - | 67/19/2 | 2/13/20 | 16/18/1 | 1.000 | 1.000 | 1.000 |

^a^ *Sus scrofa* chromosome. ^b^ Position in Sscrofa11.1 genome version. Variant positions constituting two major haplotypes are underlined. These variants are defined according to their linkage disequilibrium equal to 1 with the rs81278711 SNP (identified in the GWAS study and associated with the level of plasma kynurenine in both Large and Duroc pigs) in all three breeds, based on the whole genome sequencing data. Two major haplotypes were identified in Large White and Duroc pigs: CAGAAAA (nucleotides of the 8 polymorphisms reported in this table), indicated as rs81278711-A; TGTGGG(ACCT), indicated as haplotype rs81278711-G. ^c^ Reference (Ref) and alternative (Alt) alleles. ^d^ Consequence as derived from the Variant Predictor Effect (VEP); ^e^ Positions refer to the UniProt accession no. Q9MZS9 (accessed on: 28 March 2024). ^f^ Single amino acid polymorphism. ^g^ Genotypes are: homozygous Ref/heterozygous/homozygous Alt. ^h^ Linkage Disequilibrium (LD) is measured between the variant and the top associated marker (rs81278711) with plasma kynurenine level.

**Table S16** **Allele frequency of the *KMO* polymorphisms identified in several pig breeds and in wild boars.**

| **Breed** | **Country** | **Sequencing (no. pigs)^a^** | **Type of breed** | ***KMO* nonsynonymous mutations^b^** | | | | | | |
| --- | --- | --- | --- | --- | --- | --- | --- | --- | --- | --- |
|  |  |  |  | **g.12458867C>T** | **g.12470256A>G** | **g.12471630G>T** | **g.12486397A>G** | **g.12488061A>G** | **g.12488223A>G** | **g.12489135_12489136insCCT** |
| Large White | Italy | Individual (88) | Cosmopolitan | 0.994 | 0.875 | 0.881 | 0.869 | 0.869 | 0.869 | 0.869 |
| Duroc | Italy | Individual (35) | Cosmopolitan | 0.914 | 0.329 | 0.343 | 0.243 | 0.243 | 0.157 | 0.243 |
| Landrace | Italy | Individual (35) | Cosmopolitan | 1.000 | 0.714 | 0.714 | 0.714 | 0.714 | 0.714 | 0.714 |
| Alentejano | Portugal | DNA pool | Autochthonous | 1.000 | 1.000 | 0.969 | 1.000 | 1.000 | 0.933 | 0.979 |
| Apulo Calabrese | Italy | DNA pool | Autochthonous | 1.000 | 1.000 | 0.976 | 1.000 | 0.976 | 1.000 | 1.000 |
| Basque | France | DNA pool | Autochthonous | 1.000 | 0.952 | 0.970 | 0.886 | 0.931 | 0.941 | 0.957 |
| Bisaro | Portugal | DNA pool | Autochthonous | 1.000 | 0.804 | 0.848 | 0.900 | 0.902 | 0.907 | 0.951 |
| Black Slavonian | Croatia | DNA pool | Autochthonous | 1.000 | 0.946 | 0.909 | 0.941 | 1.000 | 0.935 | 0.889 |
| Casertana | Italy | DNA pool | Autochthonous | 1.000 | 0.914 | 0.761 | 0.725 | 0.714 | 0.771 | 0.694 |
| Cinta Senese | Italy | DNA pool | Autochthonous | 0.923 | 0.952 | 0.972 | 0.875 | 0.838 | 0.867 | 0.918 |
| Gascon | France | DNA pool | Autochthonous | 1.000 | 0.974 | 1.000 | 0.971 | 0.968 | 1.000 | 1.000 |
| Krškopolje pig | Slovenia | DNA pool | Autochthonous | 0.870 | 0.622 | 0.618 | 0.474 | 0.522 | 0.500 | 0.632 |
| Lietuvos Baltosios | Lithuania | DNA pool | Autochthonous | 0.976 | 0.778 | 0.750 | 0.690 | 0.714 | 0.619 | 0.657 |
| Lietuvos Vietines | Lithuania | DNA pool | Autochthonous | 1.000 | 0.958 | 1.000 | 0.968 | 0.930 | 0.947 | 0.975 |
| Mora Romagnola | Italy | DNA pool | Autochthonous | 1.000 | 0.412 | 0.462 | 0.314 | 0.333 | 0.324 | 0.257 |
| Moravka | Serbia | DNA pool | Autochthonous | 1.000 | 0.860 | 0.971 | 0.892 | 0.833 | 0.909 | 0.795 |
| Majorcan Black | Spain | DNA pool | Autochthonous | 1.000 | 0.921 | 0.955 | 1.000 | 1.000 | 0.958 | 1.000 |
| Nero Siciliano | Italy | DNA pool | Autochthonous | 1.000 | 0.979 | 1.000 | 0.947 | 0.943 | 0.941 | 1.000 |
| Sarda | Italy | DNA pool | Autochthonous | 1.000 | 0.919 | 0.837 | 0.951 | 0.919 | 0.895 | 0.909 |
| Schwäbisch-Hällisches Schwein | Germany | DNA pool | Autochthonous | 1.000 | 0.972 | 1.000 | 1.000 | 0.939 | 0.931 | 0.892 |
| Swallow-Bellied Mangalitsa | Serbia | DNA pool | Autochthonous | 1.000 | 1.000 | 1.000 | 0.977 | 1.000 | 0.975 | 1.000 |
| Turopolje | Croatia | DNA pool | Autochthonous | 1.000 | 0.932 | 0.895 | 0.971 | 0.966 | 0.943 | 0.933 |
| Wild boar | Italy | DNA pool | Wild population | 1.000 | 1.000 | 1.000 | 1.000 | 1.000 | 1.000 | 1.000 |

^a^ The number of pigs individually sequenced is reported within brackets for the Cosmopolitan breeds. The number of pigs that constituted the DNA pools in the autochthonous breeds was 30-35 (see Bovo et al. [15]); ^b^ The first allele at the different polymorphic sites is the reference allele reported in Sscrofa11.1. Allele frequency is reported for the reference allele. The polymorphic sites are those reported in Additional file 2, Table S15.

**Table S17** **Partial Correlation Coefficients (PCC) in Large White pigs before and after the inclusion of the genetic effect from the mQTL (genotype-corrected PCC) in the GGM construction.** Underlined, the PPC >0.3.

| **Metabolite 1** | **Metabolite 2** | **PCC** | **Genotype-corrected PCC** | **ΔPCC** |
| --- | --- | --- | --- | --- |
| **PCC that increased their strength (ΔPCC>0.10)** | | | | |
| PC ae C36:4 | PC ae C36:5 | 0.125 | 0.267 | 0.142 |
| Arg | Orn | -0.034 | 0.089 | 0.123 |
| PC ae C44:6 | Sarcosine | -0.040 | 0.078 | 0.117 |
| Orn | Phe | -0.032 | 0.077 | 0.109 |
| PC aa C34:4 | Tyr | -0.001 | 0.108 | 0.109 |
| PC aa C42:4 | Kynurenine | -0.069 | 0.039 | 0.107 |
| PC ae C34:2 | SM C18:1 | -0.033 | 0.072 | 0.106 |
| **PCC that decreased their strength (ΔPCC< -0.10)** | | | | |
| PC aa.C36:2 | PC aa C38:4 | 0.242 | 0.122 | -0.120 |
| SM C18:0 | SM C18:1 | 0.763 | 0.647 | -0.116 |
| PC ae C36:4 | lysoPC a C20:3 | -0.067 | -0.178 | -0.111 |
| PC ae C44:6 | lysoPC a C16:1 | 0.050 | -0.061 | -0.111 |
| PC ae C36:5 | PC ae C38:6 | 0.425 | 0.318 | -0.107 |
| PC ae C34:2 | Orn | -0.018 | -0.121 | -0.104 |
| PC aa C42:0 | PC ae C44:6 | 0.103 | 0.000 | -0.103 |
| **PCC that increased their strength and reached a PCC value >0.3** | | | | |
| PC ae C44:5 | PC ae C44:6 | 0.257 | 0.349 | 0.092 |
| PC aa C34:1 | PC aa C36:1 | 0.267 | 0.330 | 0.063 |
| C2 | C3 | 0.277 | 0.309 | 0.031 |
| PC aa C32:1 | PC aa C34:1 | 0.290 | 0.318 | 0.028 |
| Spermidine | Spermine | 0.274 | 0.300 | 0.027 |
| PC aa C36:1 | PC aa C36:2 | 0.291 | 0.315 | 0.024 |
| Cit | Orn | 0.297 | 0.313 | 0.016 |
| PC aa C36:2 | PC aa C36:3 | 0.296 | 0.310 | 0.014 |
