# Supplementary material for: Merging metabolomics and genomics provides a catalog of genetic factors that influence molecular phenotypes in pigs linking relevant metabolic pathways
Source: Genet Sel Evol. 2025 Mar 6;57:11. doi: 10.1186/s12711-025-00960-8 (PMC11887101; doi:10.1186/s12711-025-00960-8)

Additional file 3

**Merging metabolomics and genomics provides a catalog of genetic factors that influence molecular phenotypes in pigs linking relevant metabolic pathways**

Samuele Bovo^1*^, Anisa Ribani^1^, Flaminia Fanelli^2,3^, Giuliano Galimberti^4^, Pier Luigi Martelli^5^, Paolo Trevisi^6^, Francesca Bertolini^1^, Matteo Bolner^1^, Rita Casadio^5^, Stefania Dall’Olio^1^, Maurizio Gallo^7^, Diana Luise^6^, Gianluca Mazzoni^1^, Giuseppina Schiavo^1^, Valeria Taurisano^1^, Paolo Zambonelli^1^, Paolo Bosi^6^, Uberto Pagotto^2,3^, Luca Fontanesi^1*^

^1^Animal and Food Genomics Group, Division of Animal Sciences, Department of Agricultural and Food Sciences, University of Bologna, Bologna, Italy

^2^Endocrinology Research Group, Center for Applied Biomedical Research, Department of Medical and Surgical Sciences, University of Bologna, Bologna, Italy

^3^Division of Endocrinology and Prevention and Care of Diabetes, IRCCS Azienda Ospedaliero-Universitaria di Bologna, Policlinico di Sant’Orsola, Bologna, Italy

^4^Department of Statistical Sciences “Paolo Fortunati”, University of Bologna, Bologna, Italy

^5^Biocomputing Group, Department of Pharmacology and Biotechnology, University of Bologna, Bologna, Italy

^6^Laboratory on Animal Nutrition and Feeding for Livestock Sustainability and Resilience, Division of Animal Sciences, Department of Agricultural and Food Sciences, University of Bologna, Bologna, Italy

^7^Associazione Nazionale Allevatori Suini, Roma, Italy

*Corresponding authors: Samuele Bovo (SB); Luca Fontanesi (LF).

E-mail addresses: [samuele.bovo@unibo.it](mailto:samuele.bovo@unibo.it); [luca.fontanesi@unibo.it](mailto:luca.fontanesi@unibo.it)

**Figure S1.** **Simplified representation of the kynurenine pathway (KP), with information used in the kinetic modelling.** Metabolites are shown in boxes and reactions directions are shown by arrows labelled with the acronym of the enzymes. Abbreviations for the metabolites: Trp, tryptophan; KYN, kynurenine; XA, xanthurenic acid; AA, anthranilic acid; KA, kynurenic acid; HK, 3-hydroxykynurenine; HAA, 3-hydroxyanthranilic acid; QUIN, quinolinic acid. Abbreviations for the enzymes: TDO, tryptophan 2,3-dioxygenase; KYNU1, kynureninase; KYNU2, kynureninase; KMO, kynurenine 3-monoxygenase; KAT1, kynurenine aminotransferase; KAT2, kynurenine aminotransferase; 3HAO, 3-hydroxyanthranilate 3,4-dioxygenase.


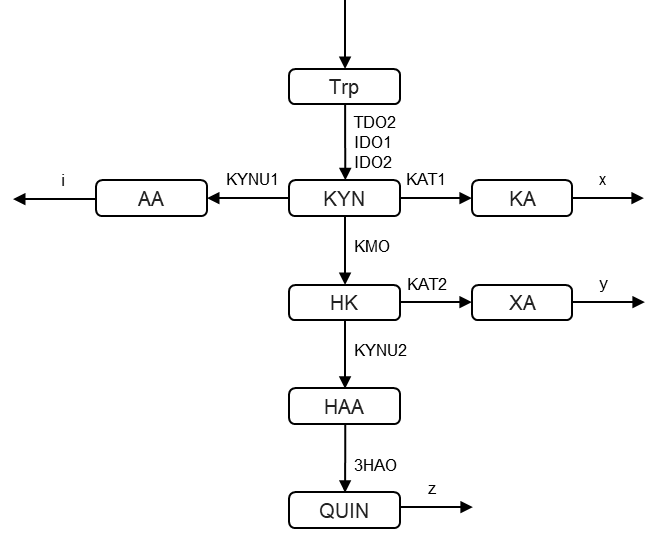


**Figure S2.** **Relationship between heritability and number of carbon atoms (with only one double bound) present in acylcarnitines, glycerophospholipids and sphingomyelins.**


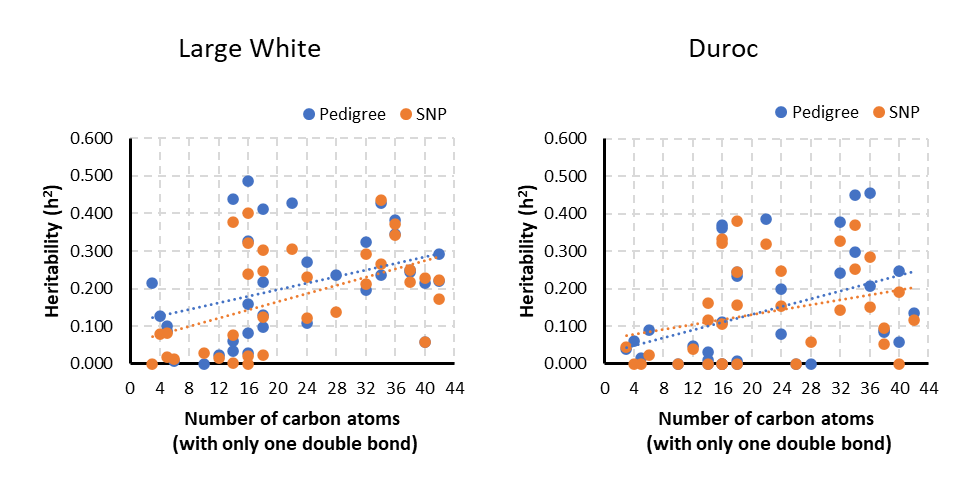


**Figure S3.** **Relationship between heritability and number of carbon atoms or double bounds within the phosphatidylcholine group.** a) Phosphatidylcholine acyl-alkyls with three double bounds (PC ae CX:3). b) Phosphatidylcholines acyl-alkyls with 36 carbon atoms and 1 to 5 double bonds (PC ae C36:X, X=1,..,5). c) Phosphatidylcholines acyl-alkyls with 38 carbon atoms and 1 to 6 double bonds (PC ae C38:X, X=1,..,6).


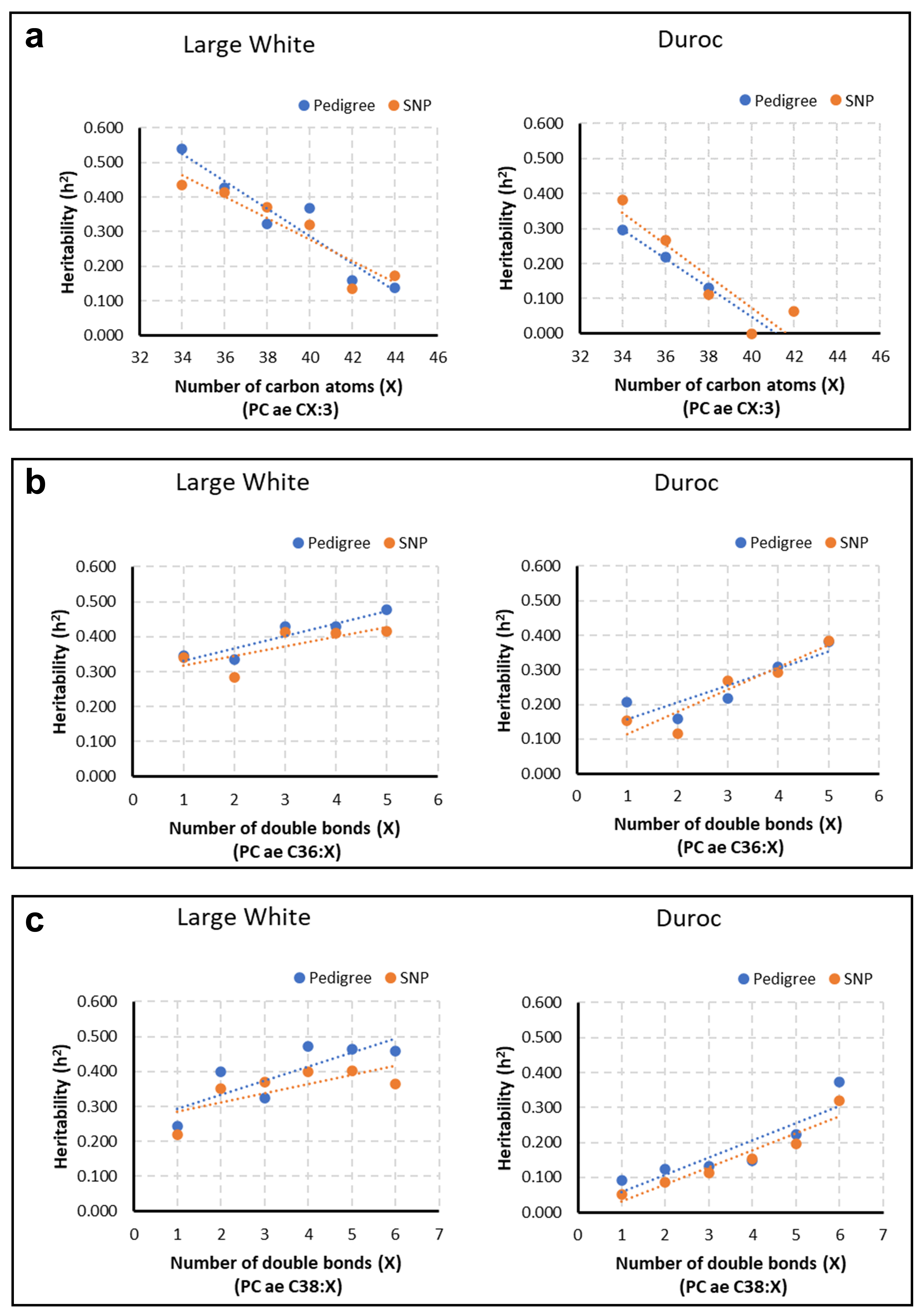


**Figure S4.** **Complete profiles of associations of the mQTL (with *FADS6* as candidate gene) over the metabolite pairs (ratios) of lysophosphatidylcholines and phosphatidylcholines.** The -log_10_(*P*) is reported.

**
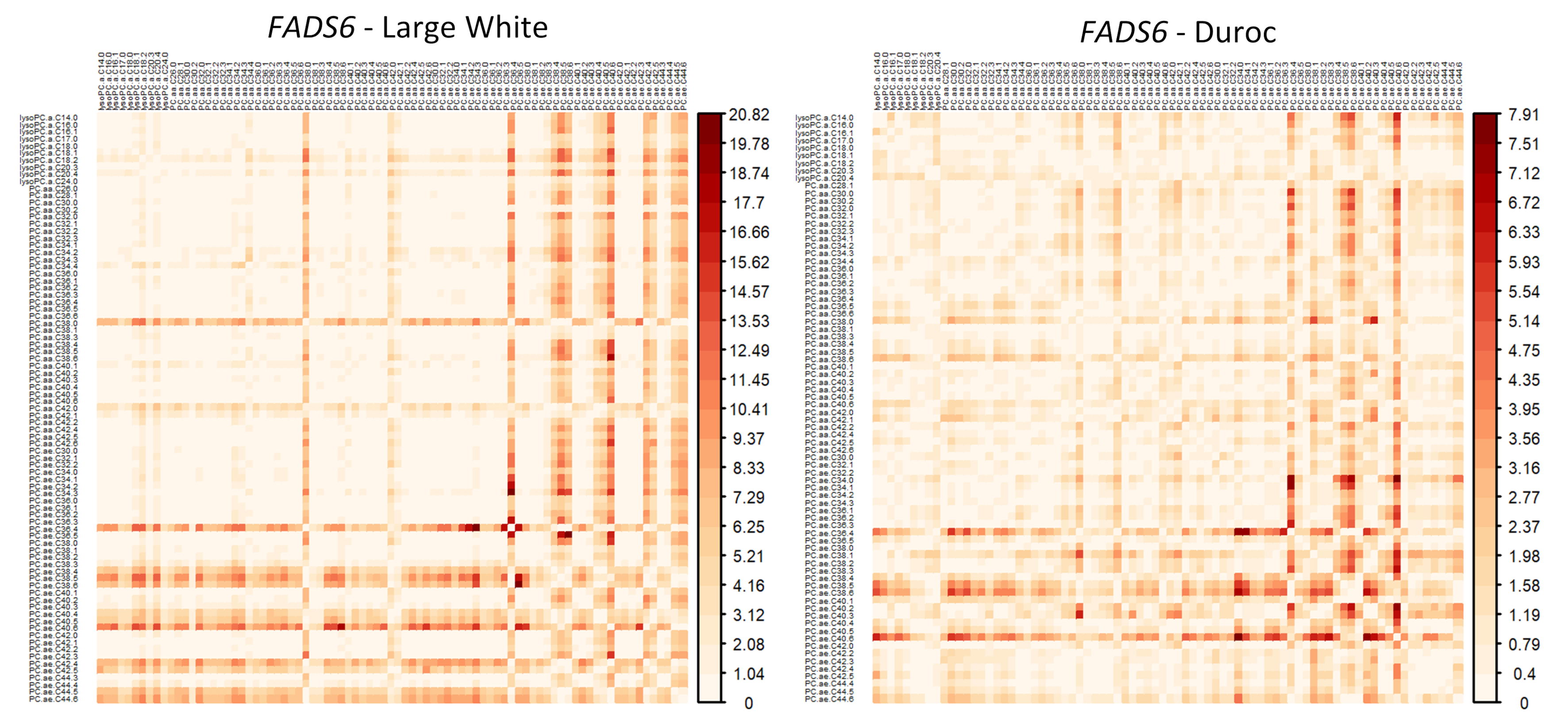
**

**Figure S5.** **Complete profiles of associations of the mQTL (with *PLIN1* as candidate gene) over metabolite pairs (ratios) of lysophosphatidylcholines and phosphatidylcholines.** The -log_10_(*P*) is reported.

**
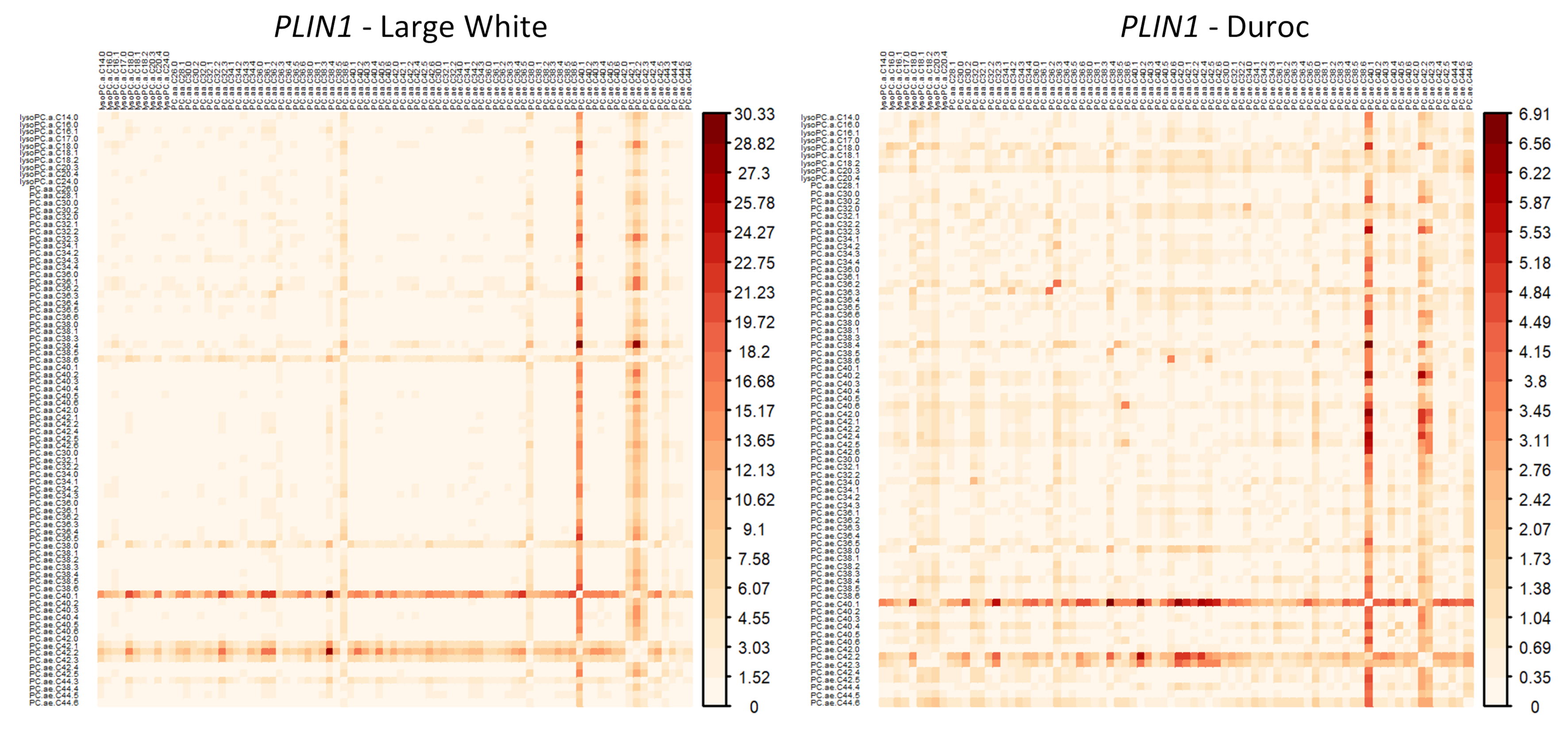
**

**Figure S6. Complete profiles of associations of the mQTL (with *FADS1/FADS2/FADS3* as candidate genes) over metabolite pairs (ratios) of lysophosphatidylcholines and phosphatidylcholines.** The -log_10_(*P*) is reported.

**
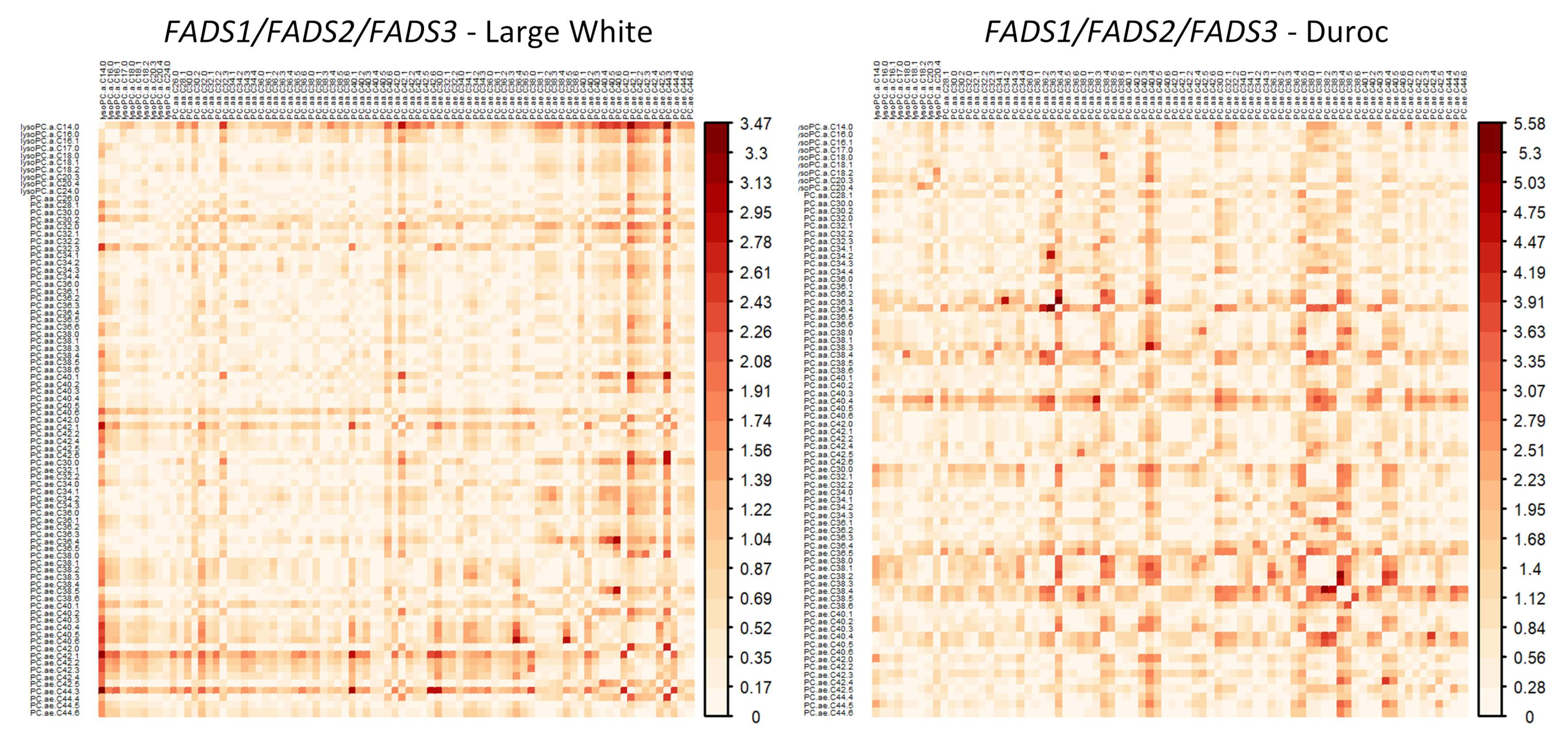
**

**Figure S7.** **Complete profiles of associations of the mQTL (with *FAR1* as candidate gene) over metabolite pairs (ratios) of lysophosphatidylcholines and phosphatidylcholines.** The -log_10_(*P*) is reported.

**
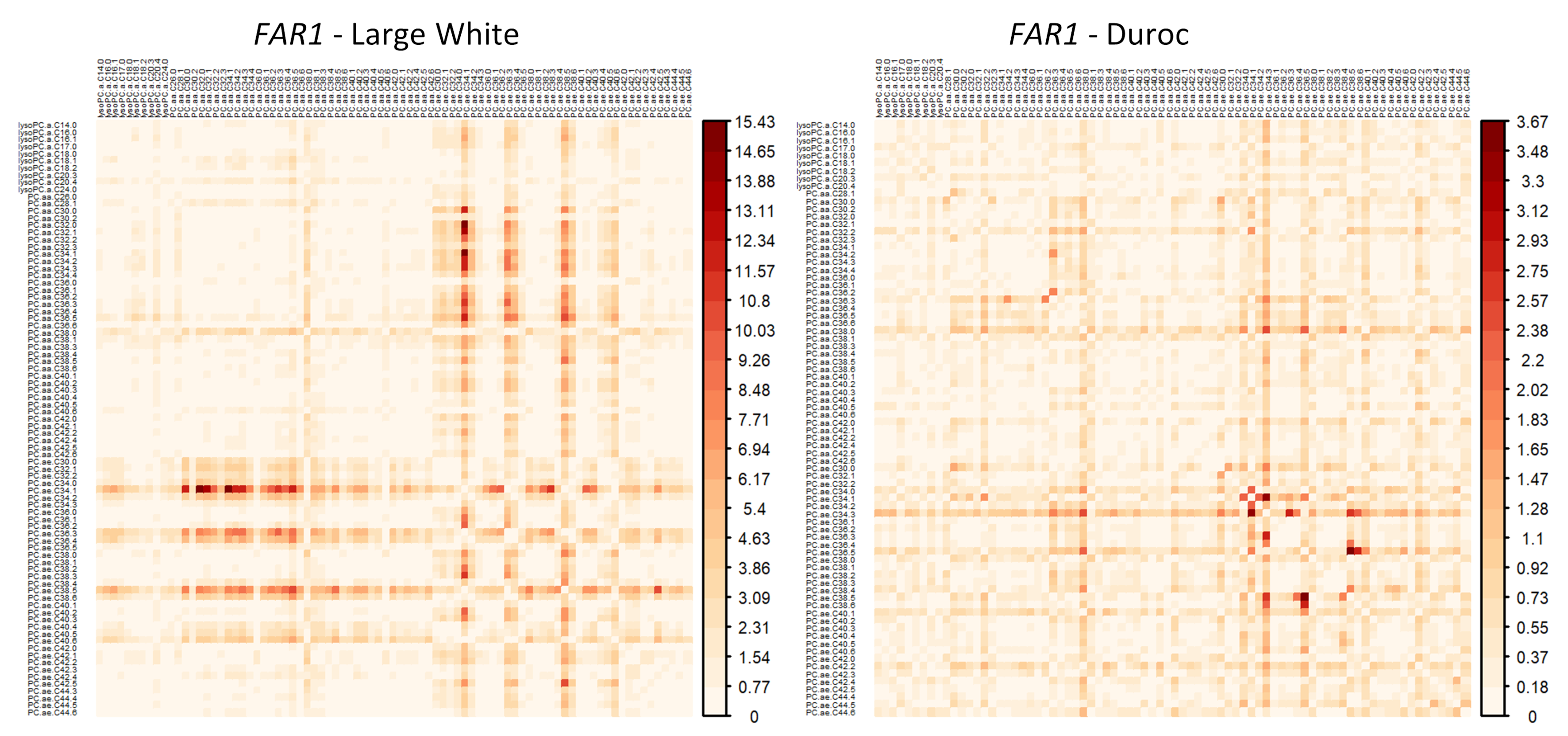
**

**Figure S8.** **Complete profiles of associations of the mQTL (with *LPCAT2/SMPD3* as candidate genes) over metabolite pairs (ratios) of lysophosphatidylcholines and phosphatidylcholines.** The -log_10_(*P*) is reported.


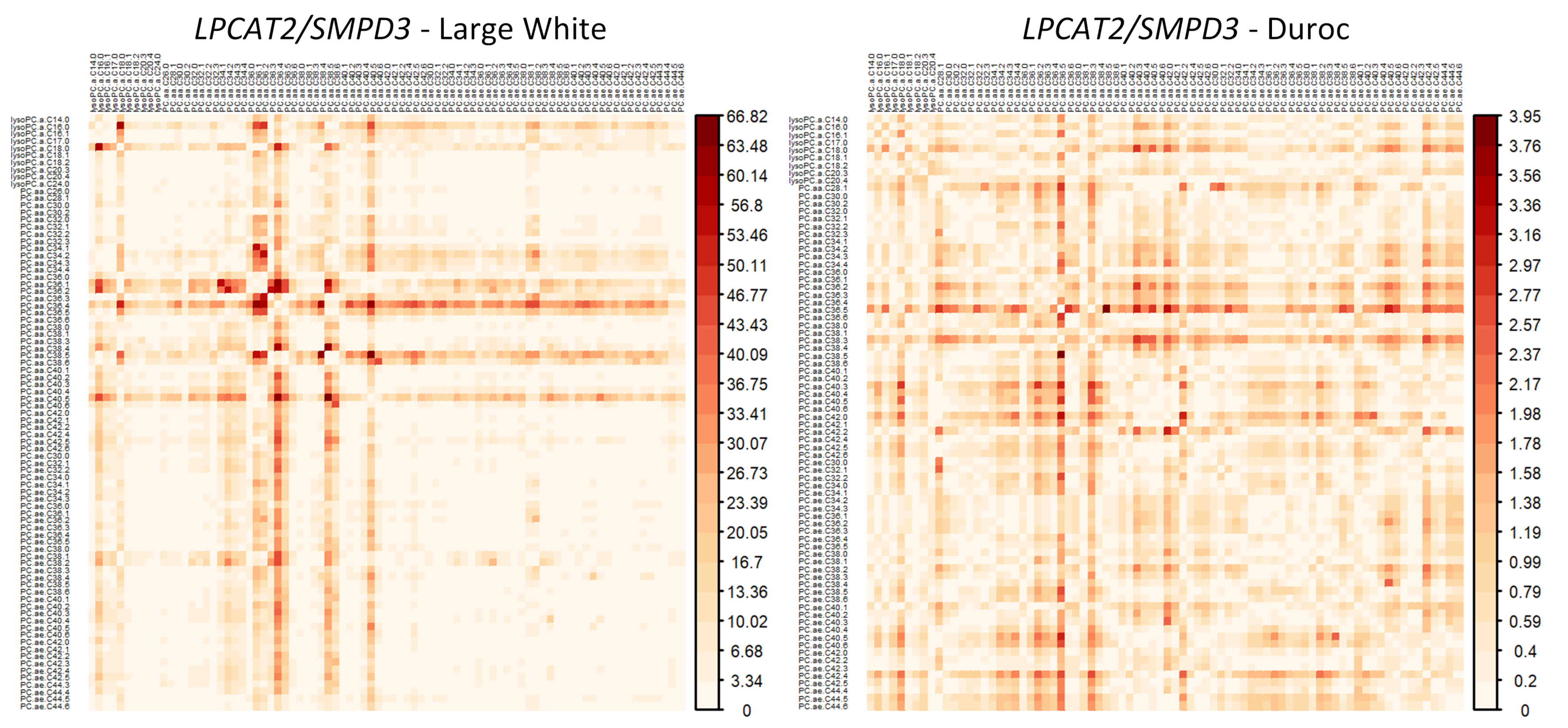


**Figure S9.** **Plasma concentration of metabolites of the kynurenine pathway (KP) in Large White × Landrace piglets homozygous for the alternative *KMO* haplotypes (indicated with the tag SNP: rs81278711-AA and rs81278711-GG) at the basal tryptophan level (no tryptophan supplementation).** Abbreviations: Trp, tryptophan; KYN, kynurenine; XA, xanthurenic acid; AA, anthranilic acid; KA, kynurenic acid; HK, 3-hydroxykynurenine; HAA, 3-hydroxyanthranilic acid; QUIN, quinolinic acid.

**
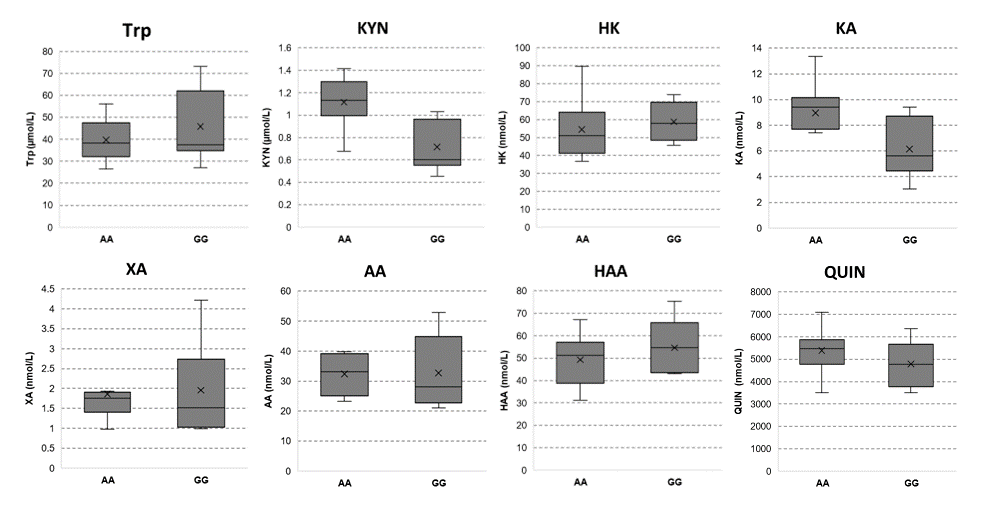
**

**Figure S10.** **Plasma concentration of metabolites of the kynurenine pathway (KP) in Large White × Landrace piglets homozygous for the alternative *KMO* haplotypes (indicated with the tag SNP: rs81278711-AA and rs81278711-GG) after tryptophan supplementation.** Abbreviations: Trp, tryptophan; KYN, kynurenine; XA, xanthurenic acid; AA, anthranilic acid; KA, kynurenic acid; HK, 3-hydroxykynurenine; HAA, 3-hydroxyanthranilic acid; QUIN, quinolinic acid.


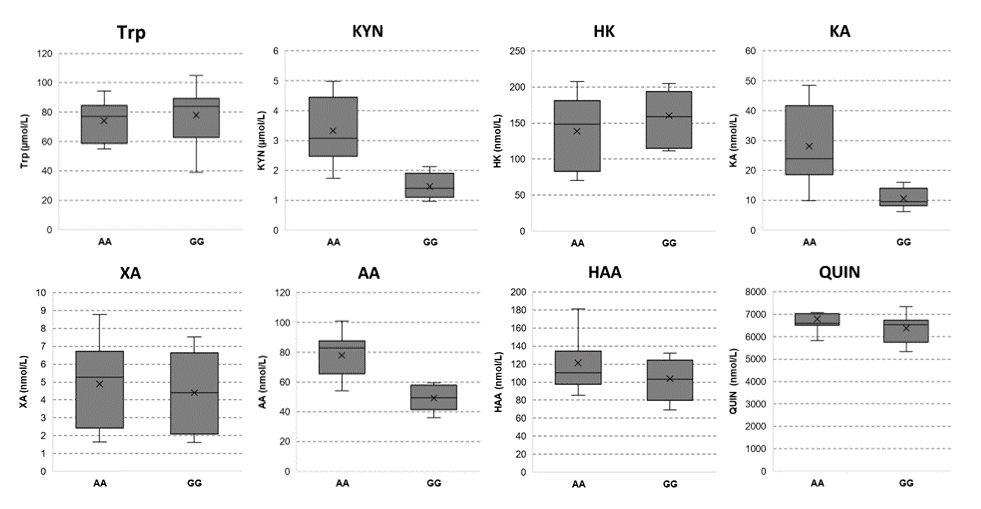


**Figure S11.** **Relationships between kynurenine (KYN) levels and its first neighbor metabolites as stratified for the alternative *KMO* haplotypes (indicated with the tag SNP: rs81278711-AA and rs81278711-GG).** Data are from the nutrigenetic study in the Large White × Landrace piglets, after tryptophan supplementation. a) Haplotypes differs in [KYN] but not in [Trp]. b) Haplotypes differs in [KYN] but not in [HK]. c) Haplotypes differs in [KYN] and in [KA]. d) Haplotypes differs in [KYN] and in [AA]. Abbreviations: Trp, tryptophan; KYN, kynurenine; AA, anthranilic acid; KA, kynurenic acid; HK, 3-hydroxykynurenine.

**
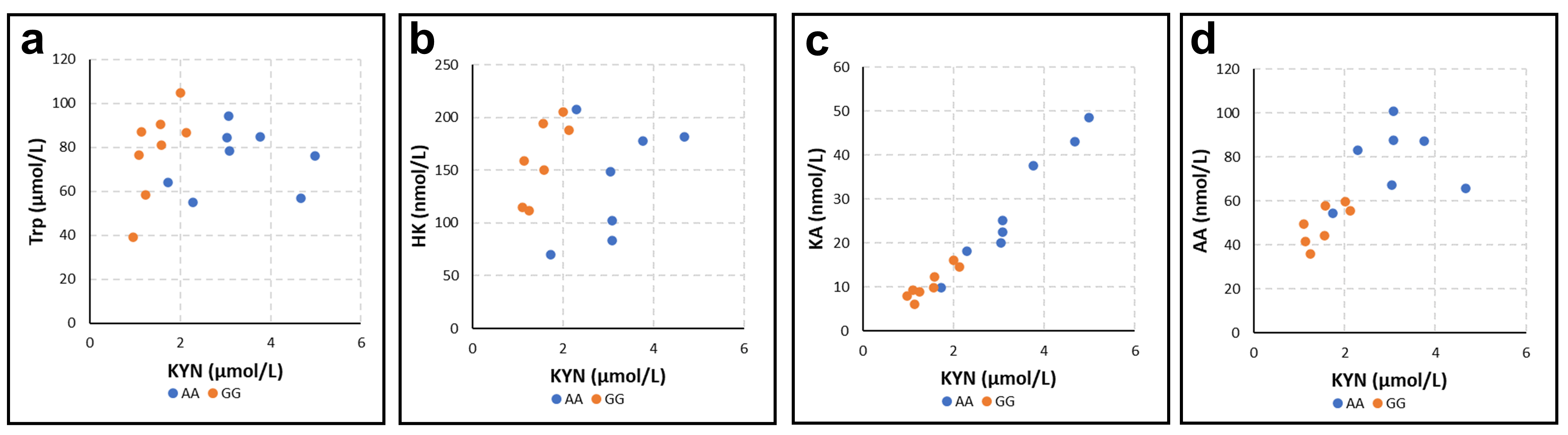
**

**Figure S12. Results of gene expression and Western blotting analyses for KMO.** Liver samples of pigs carrying two different *KMO* genotypes (rs81278711-AA and rs81278711-GG) were analysed. A) Results of the qPCR analyses including average Ct values for *KMO* and *B2M* genes obtained for the pigs with different *KMO* genotypes. The relative gene expression was obtained as 2−∆∆Ct and presented as averaged measures, considering all animals with the same genotype (as no gene expression differences were observed between the two genotypes in both experimental designs, namely the performance tested Large White gilts and the crossbred pigs of the nutrigenetic experiment; t-test, *P* = 0.65). B) Examples of Western blot images from 6 pigs (pigs 1, 2 and 3 with genotype rs81278711-AA; pigs 4, 5 and 6 with genotype rs81278711-GG) and the corresponding band lines stained with Coomassie Brilliant Blue used to normalize the samples. The statistical analysis showed no significant band differences between the two *KMO* genotypes (P = 0.202). M: Molecular weight marker.


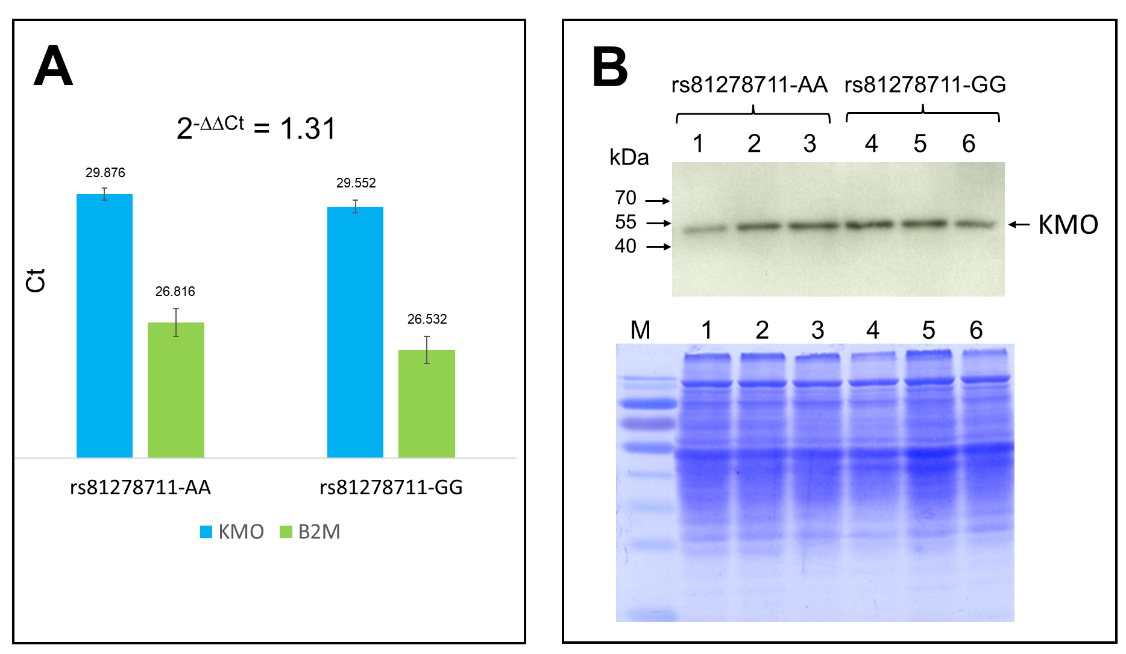


**Figure S13.** **Domains of the pig KMO protein.** Domains are annotated with Interpro tool on the protein KMO_PIG (Q9MZS9). FAD-binding domain is detected by the PFAM entry PF01494 (FAD_binding_3), a member of the clan NADP_Rossmann. Positions corresponding to the differences in the two haplotypes are indicated. The variations S95F, Q135R and V178L occur in the FAD binding domain and may influence the cofactor binding.


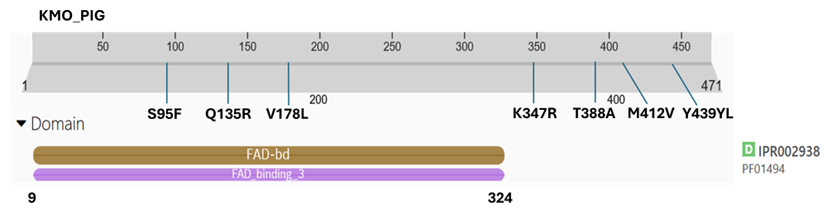


**Figure S14.** **Inclusion of the mQTL information in GGM and correlation analyses.** a) Partial Correlation Coefficients (PCC) vs. genotype-corrected PCC. b) Pearson’s correlations (*r*) vs. genotype-corrected Pearson’s correlations.


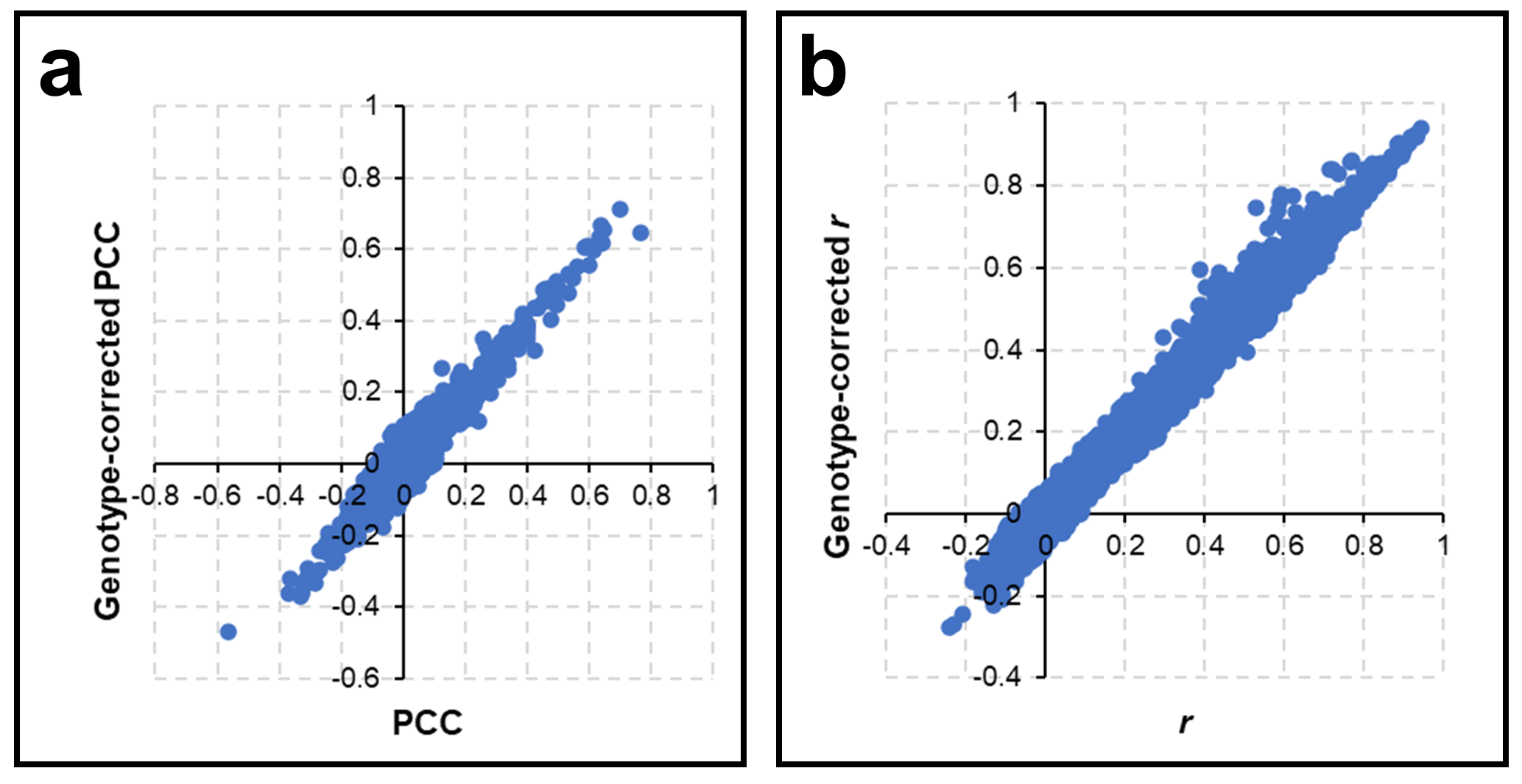


**Figure S15.** **Inclusion of the effect of the *SMPD3/LPCAT2* genotypes in the estimation of metabolite correlations.** a) Effect of the *SMPD3/LPCAT2* genotypes on PC aa C36:4 and PC aa C36:1 metabolites (different direction of the *β* of association). b) Relationship between PC aa C36:4 and PC aa C36:1 metabolites. c) Effect of the *SMPD3/LPCAT2* genotypes on PC aa C36:4 and PC aa C36:1 metabolites after inclusion of genetic information. d) Relationship between PC aa C36:4 and PC aa C36:1 metabolites after inclusion of genetic information. In b) and d), different colors represent the pigs with different *SMPD3/LPCAT2* genotypes.


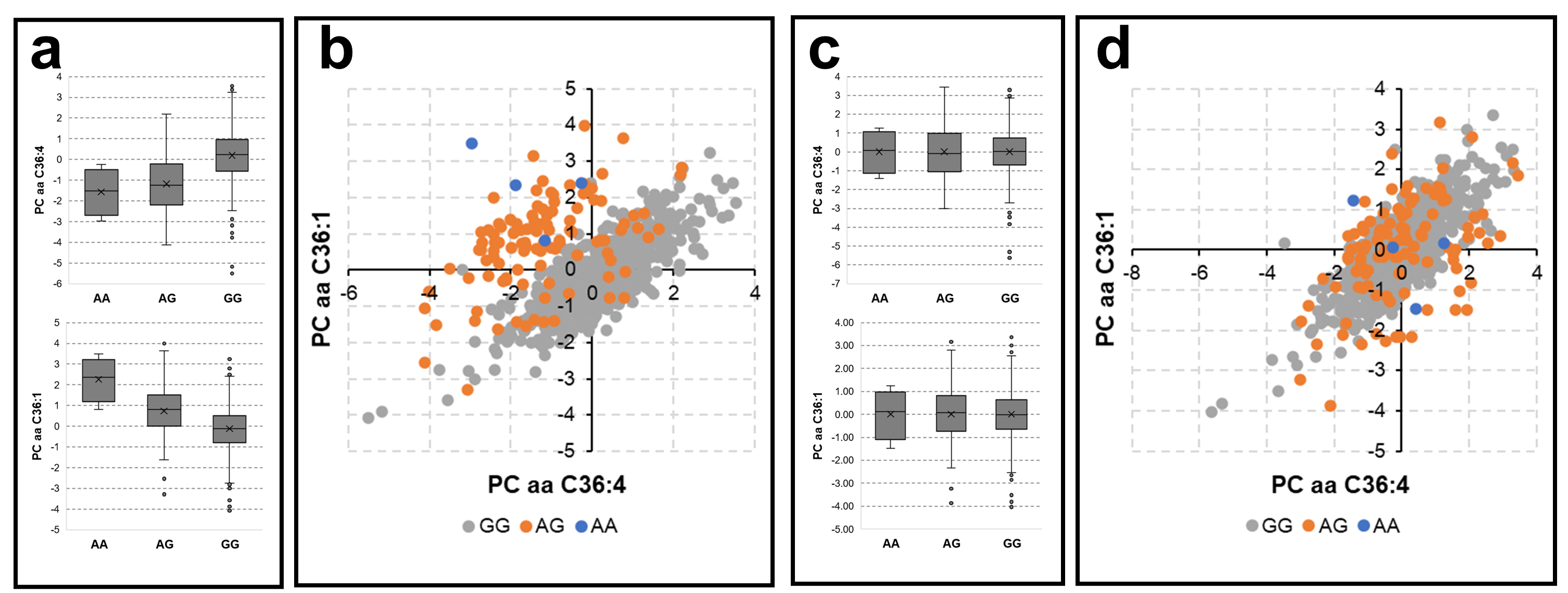


**Figure S16.** **Inclusion of the effect of the *SLC6A4* genotypes in the estimation of metabolite correlations.** a) Effect of the *SLC6A4* genotypes on serotonin and taurine metabolites (same direction of the β of association). b) Relationship between serotonin and taurine metabolites. c) Effect of the *SLC6A4* genotypes on serotonin and taurine metabolites after inclusion of genetic information. d) Relationship between serotonin and taurine metabolites after inclusion of genetic information. In b) and d), different colors represent the pigs with different *SLC6A4* genotypes.


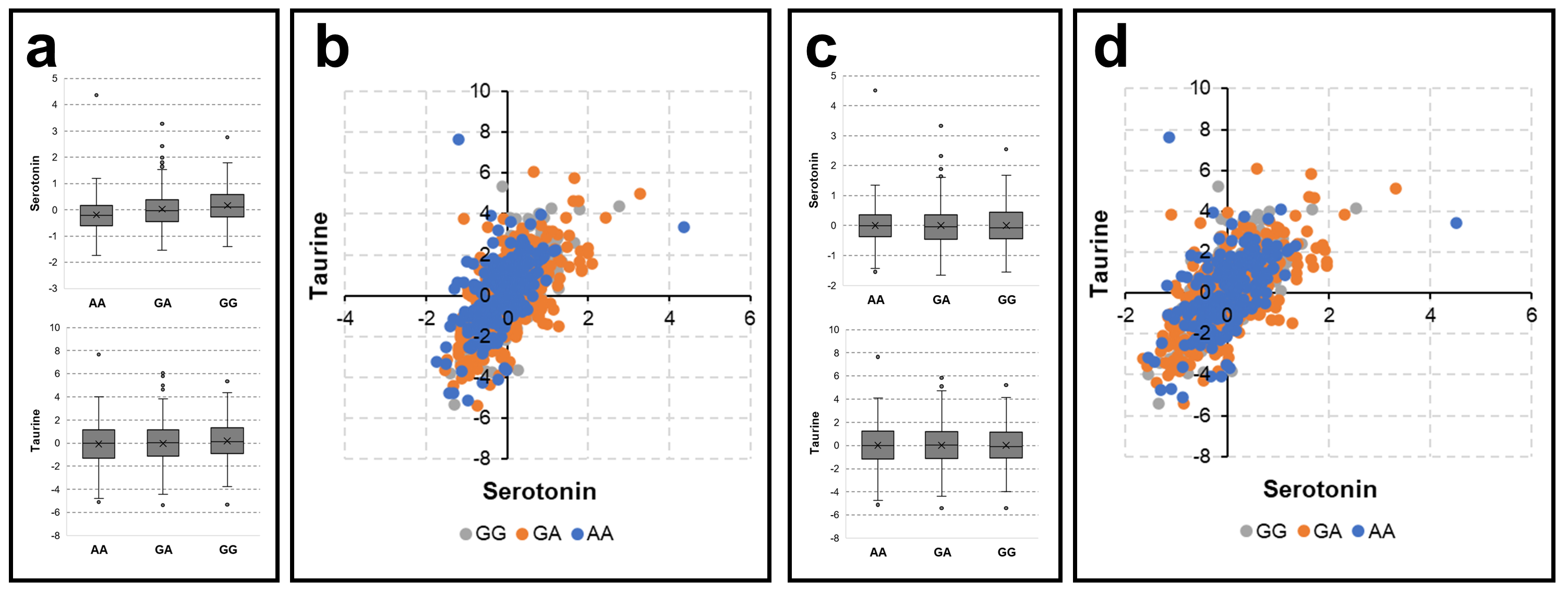


**Figure S17.** **Comparative analysis of Partial Correlation Coefficients (PCC) and Pearson’s correlation coefficients between a few sphingomyelins and information on the associated genes as reported in the GWAS.** Results are from the Large White population. a) Partial correlation coefficients (|PCC|≥0.3). b) Correlation coefficients (|*r*|≥0.3). c) mQTL associated with single metabolites. Gray edges represent correlation coefficients. d) mQTL associated with metabolite ratios (arrows indicates affected metabolite pairs). Gray edges represent correlation coefficients. Information on the relevant mQTL number is reported in Additional file 1: Table S11.


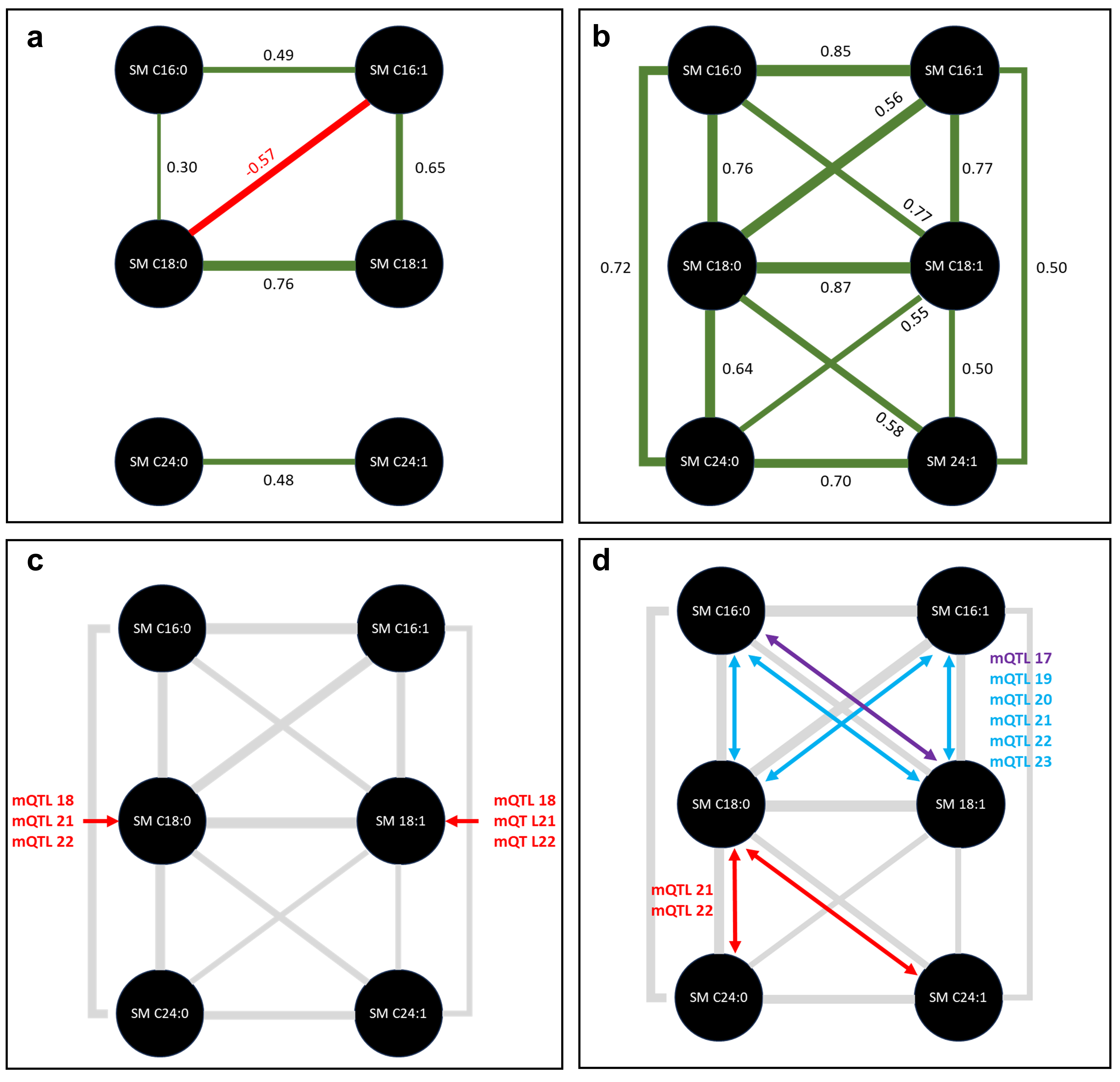


**Figure S18.** **Comparative analysis of partial correlation coefficients (PCC) and Pearson’s correlation coefficients between a few lysophosphatidylcholines and information on the associated genes as reported in the GWAS.** Results are from the Large White population. Gray edges represent correlation coefficients. Metabolites and ratios targeted by mQTL 41 (*LPCAT2/SMPD3*) are highlighted by red and blue arrows, respectively. Information on the relevant mQTL number is reported in Additional file 1, Table S11.


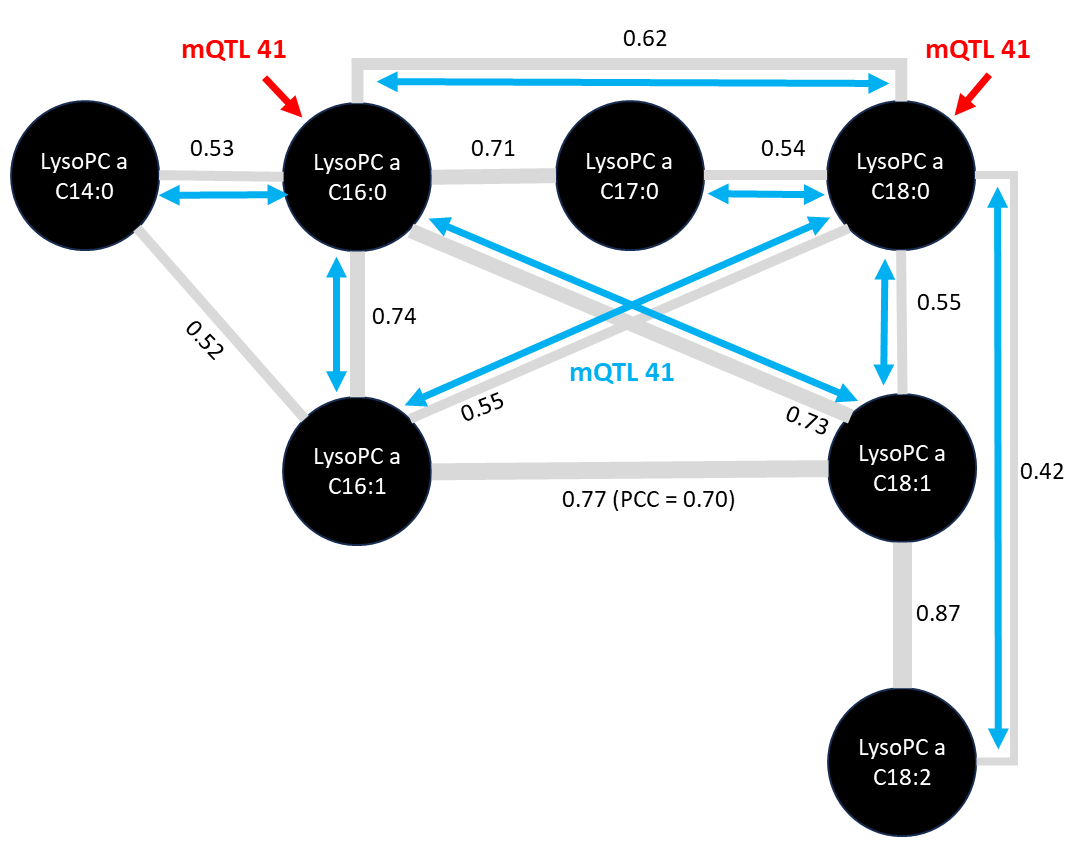


**Figure S19.** **Manhattan plot results for serotonin and taurine GWAS in Large White pigs.** Green dots represent suggestively and significantly associated SNPs (*P*<5.0×10^-5^).


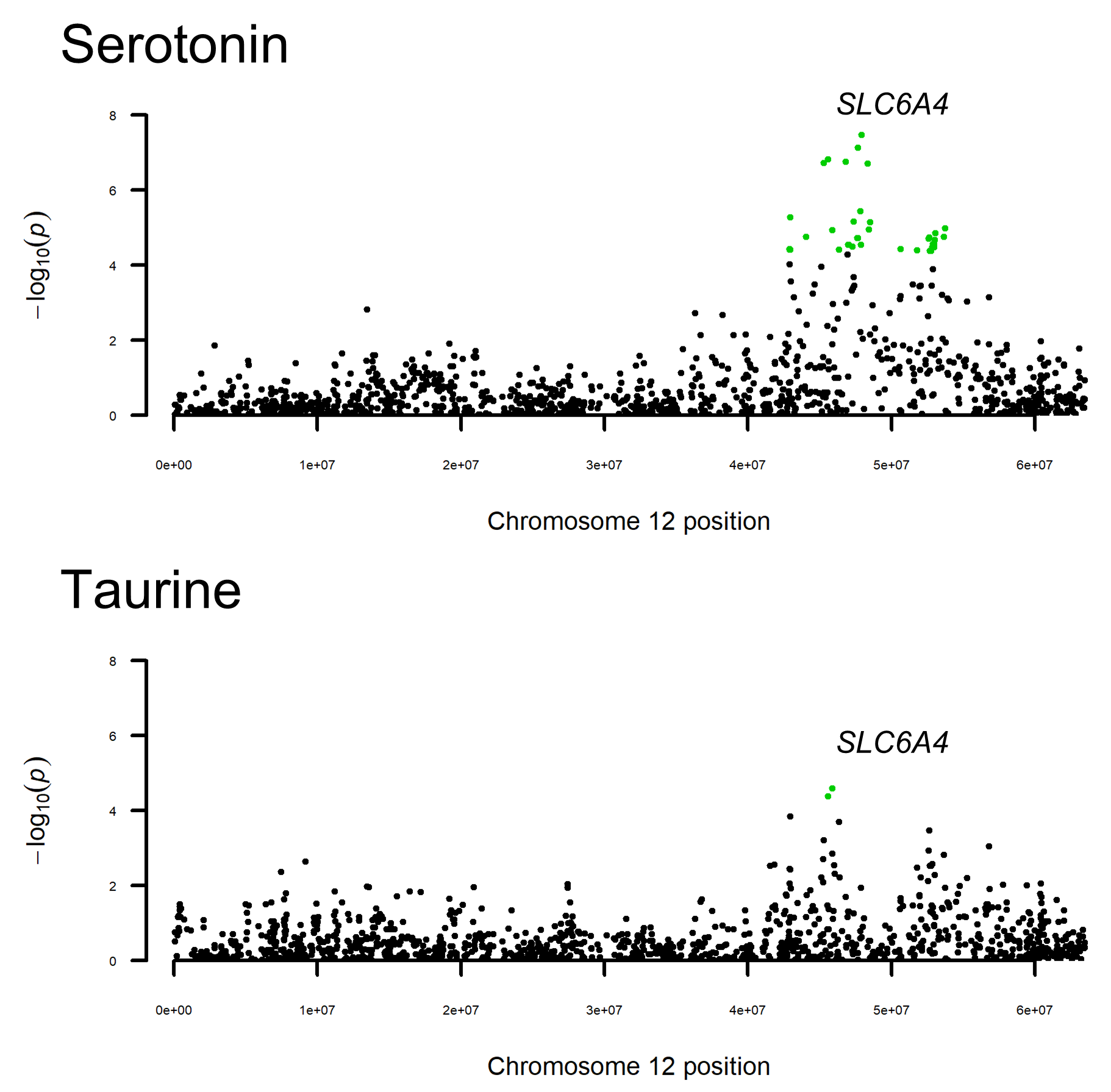

Supplement: Supplementary file 3 — Additional file 3: Figure S1. Simplified representation of the kynurenine pathway (KP), with information used in the kinetic modelling. Description: Metabolites are shown in boxes and reactions directions are shown by arrows labelled with the acronym of the enzymes. Abbreviations for the metabolites: Trp, tryptophan; KYN, kynurenine; XA, xanthurenic acid; AA, anthranilic acid; KA, kynurenic acid; HK, 3-hydroxykynurenine; HAA, 3-hydroxyanthranilic acid; QUIN, quinolinic acid. Abbreviations for the enzymes: TDO, tryptophan 2,3-dioxygenase; KYNU1, kynureninase; KYNU2, kynureninase; KMO, kynurenine 3-monoxygenase; KAT1, kynurenine aminotransferase; KAT2, kynurenine aminotransferase; 3HAO, 3-hydroxyanthranilate 3,4-dioxygenase. Figure S2. Relationship between heritability and number of carbon atoms (with only one double bound) present in acylcarnitines, glycerophospholipids and sphingomyelins. Figure S3. Relationship between heritability and number of carbon atoms or double bounds within the phosphatidylcholine group. Description: a) Phosphatidylcholine acyl-alkyls with three double bounds (PC ae CX:3). b) Phosphatidylcholines acyl-alkyls with 36 carbon atoms and 1 to 5 double bonds (PC ae C36:X, X=1,..,5). c) Phosphatidylcholines acyl-alkyls with 38 carbon atoms and 1 to 6 double bonds (PC ae C38:X, X=1,..,6). Figure S4. Complete profiles of associations of the mQTL (with FADS6 as candidate gene) over the metabolite pairs (ratios) of lysophosphatidylcholines and phosphatidylcholines. Description: The -log10(P) is reported. Figure S5. Complete profiles of associations of the mQTL (with PLIN1 as candidate gene) over metabolite pairs (ratios) of lysophosphatidylcholines and phosphatidylcholines. Description: The -log10(P) is reported. Figure S6. Complete profiles of associations of the mQTL (with FADS1/FADS2/FADS3 as candidate genes) over metabolite pairs (ratios) of lysophosphatidylcholines and phosphatidylcholines. Description: The -log10(P) is reported. Figure S7. Compl [file 12711_2025_960_MOESM3_ESM.docx]
