# Supplementary material for: Merging metabolomics and genomics provides a catalog of genetic factors that influence molecular phenotypes in pigs linking relevant metabolic pathways
Source: Genet Sel Evol. 2025 Mar 6;57:11. doi: 10.1186/s12711-025-00960-8 (PMC11887101; doi:10.1186/s12711-025-00960-8)
Supplement: Supplementary file 4 — Additional file 4: Text S1. Detail on quantitative real time PCR and western blotting analyses of KMO. Text S2. Relationships between heritability estimates of some metabolites in pigs and their chemical structures. Text S3. Comparative information on candidate genes between humans and pigs. Text S4. Identification and annotation of variants from whole genome resequencing data. Text S5. Evaluation of KMO mutations in relation to its protein sequence and structure. Text S6. Inclusion of the genetic effect in the Gaussian Graphical Model (GGM) construction. Text S7. Merging GGM and GWAS results. [file 12711_2025_960_MOESM4_ESM.docx]

Additional file 4

**Merging metabolomics and genomics provides a catalog of genetic factors that influence molecular phenotypes in pigs linking relevant metabolic pathways**

Samuele Bovo^1*^, Anisa Ribani^1^, Flaminia Fanelli^2,3^, Giuliano Galimberti^4^, Pier Luigi Martelli^5^, Paolo Trevisi^6^, Francesca Bertolini^1^, Matteo Bolner^1^, Rita Casadio^5^, Stefania Dall’Olio^1^, Maurizio Gallo^7^, Diana Luise^6^, Gianluca Mazzoni^1^, Giuseppina Schiavo^1^, Valeria Taurisano^1^, Paolo Zambonelli^1^, Paolo Bosi^6^, Uberto Pagotto^2,3^, Luca Fontanesi^1*^

^1^Animal and Food Genomics Group, Division of Animal Sciences, Department of Agricultural and Food Sciences, University of Bologna, Bologna, Italy

^2^Endocrinology Research Group, Center for Applied Biomedical Research, Department of Medical and Surgical Sciences, University of Bologna, Bologna, Italy

^3^Division of Endocrinology and Prevention and Care of Diabetes, IRCCS Azienda Ospedaliero-Universitaria di Bologna, Policlinico di Sant’Orsola, Bologna, Italy

^4^Department of Statistical Sciences “Paolo Fortunati”, University of Bologna, Bologna, Italy

^5^Biocomputing Group, Department of Pharmacology and Biotechnology, University of Bologna, Bologna, Italy

^6^Laboratory on Animal Nutrition and Feeding for Livestock Sustainability and Resilience, Division of Animal Sciences, Department of Agricultural and Food Sciences, University of Bologna, Bologna, Italy

^7^Associazione Nazionale Allevatori Suini, Roma, Italy

*Corresponding authors: Samuele Bovo (SB); Luca Fontanesi (LF).

E-mail addresses: [samuele.bovo@unibo.it](mailto:samuele.bovo@unibo.it); [luca.fontanesi@unibo.it](mailto:luca.fontanesi@unibo.it)

**Text S1.** **Details on quantitative real time PCR and Western blotting analyses of KMO**

Liver is considered one of the most relevant tissues in amino acid metabolism and is a tissue where *KMO* is highly expressed [1, 2]. Liver samples of the pigs indicated in Methods and stored at -80 °C were used to extract total RNA using the RNeasy kit (QIAGEN, Hilden, Germany), following the manufacture’s indications. Contaminating DNA was subsequentially removed using the TURBO-DNase (Thermo Fisher Scientific, Waltham, MA, USA). Quality of the RNA was then evaluated using the 2100 Bioanalyzer Instrument (Agilent, Santa Clara, CA, USA). RNA (700 ng from each sample) was then reverse transcribed to cDNA with the GeneAmp RNA Core Kit (Thermo Fisher Scientific) containing MuLV Reverse Transcriptase using random hexamers. The reaction was carried out in a SimpliAmp thermal cycler (Thermo Fisher Scientific) with the following cycle: 25 °C 10 min, 37 °C 30 min, 95 °C 5 min. Porcine *KMO* was amplified using a primer pair that targeted a 163 bp region that spans from exon 9 to exon 11 (forward: 5’-AGTACATTCCTCACGGCTACA-3’; reverse: 5’-TCAAAGGGCATGAACAAAGTA-3’). The porcine *B2M* was used as housekeeping gene for the liver gene expression, according to Nygard et al. [3]. PCR primers (forward: 5’-CAAGATAGTTAAGTGGGATCGAGAC-3’; reverse: 5’-TGGTAACATCAATACGATTTCTGA-3’), that amplified a fragment of 161 bp, were from Park et al. [4]. For each transcript a standard curve was constructed using the purified PCR product generated for each specific primer pair. Single reactions were prepared for each cDNA along with each serial dilution using the Kapa SYBR Fast qPCR Master Mix kit (Kapa Biosystems, Roche, Basel, Switzerland). Each PCR reaction included 3 replicates per sample, a negative control (without reverse transcriptase) to confirm the absence of genomic DNA and the housekeeping gene. Each reaction consisted of 20 μL containing 1 μL of cDNA products (250 ng) and 5 pmol of each primer. The real time qPCR was run on a QuantStudio 7 instrument (Thermo Fisher Scientific) with the following cycling conditions: 1 cycle of denaturation at 95 °C for 10 min, followed by 40 cycles of amplification (95 °C for 15 sec and 60 °C for 1 min). A dissociation curve was constructed for each primer pair after the PCR reaction to verify the presence of one gene-specific peak and the absence of primer dimers. An average Ct value was then calculated for the three replicates and for each group of pigs (the two genotypes) and for each experimental design (piglets and adult Large White gilts). The relative gene expression was obtained as 2^−∆∆Ct^ and presented as averaged measures, considering all animals with the same genotype (as no gene expression differences were observed between the two genotypes in both experimental designs; t-test, *P* = 0.65).

Proteins were extracted from the same liver tissues of the same pigs, with 3 technical replicates for each sample, using the protocol described in Bovo et al. [5]. Briefly, extraction was carried out from ~100 mg of liver tissue homogenized in 1 mL of extraction buffer [0.1% SDS (Thermo Fisher Scientific), 100 mM Tris/HCl (Thermo Fisher Scientific) pH 7.6, 10 mM DTT (Thermo Fisher Scientific)]. Liver samples were homogenized for 2 min using a homogenizer (G50 Tissue Grinder, Coyote Bioscience, Inc., China) in a 1.5 mL tube immersed into liquid nitrogen. The lysates were vortexed at room temperature for 3 min and then centrifuged at 16,000 × rcf at 4 °C for 5 min. Samples were heated at 56 °C for 30 min and then centrifuged at 16,000 × rcf at 20 °C for 20 min. The supernatants were transferred into new labelled tubes, were mixed, aliquoted and stored at -20 °C until subsequent analyses. Qubit™ Fluorometric Quantitation was used to determine the protein concentration according to the manufacturer’s protocol (Thermo Fisher Scientific). Extracted proteins were separated by SDS-PAGE using 12% polyacrylamide resolving gels with a 4% stacking gel (Gibco BRL/Thermo Fisher Scientific). Mouse brain tissue lysate - total protein validated for Western Blotting (ab4022; Abcam, Cambridge, UK), were used as controls. Anti-KMO antibody (ab130959; Abcam, Cambridge, UK) was used in the Western blotting analyses. Twenty micrograms of protein were loaded in each lane for the samples that were later incubated with the KMO primary antibody. Proteins were electrophoretically transferred to 0.45 µm PVDF membranes, blocked with 5% non-fat milk and then incubated overnight with the primary antibodies (2-8 °C) with a concentration of 2µg/mL. The secondary antibody used was Anti-Rabbit IgG (H+L), HRP Conjugate (1:10000; W4011; Promega). Membranes were finally subjected to electrochemiluminescent detection using ECL Plus Western Blotting Detection Reagent (GE Healthcare; Chicago, IL, USA), scanned and analyzed using Image J software [6]. No signals were detected for the mouse brain proteins. The average band density was then normalized to the average band density of the same lane to control for any loading inaccuracies [7]. The normalized average band density of KMO protein was analyzed across the two genotypes using ANOVA and Tukey’s test in R [8].

**Text S2. Relationships between heritability of some metabolites in pigs and their chemical structures**

Following the report of Hagenbeek et al. [9] that reviewed the heritability of blood metabolites derived from several studies in humans, we evaluated whether the heritability we estimated in pigs for certain analyzed metabolites (acylcarnitines, glycerophospholipids and sphingomyelins) was correlated with their chemical structure properties. We focused on these three families of analytes because we produced data in pigs on numerous molecules within these groups, each with different chemical structures and characteristics. When we examined the relationship between heritability and the number of carbon atoms and double bonds, we observed some correlations. Specifically, when looking at metabolites with one double bond, we found a positive correlation with an increasing number of carbon atoms (Large White, *r* =0.70 with h^2^_P_ and *r* = 0.66 with h^2^_SNP_; Duroc, *r* = 0.46 with h^2^_P_ and *r* = 0.56 with h^2^_SNP_; Figure S2). This overall pattern, showing an increase in heritability with a higher number of carbon atoms in the biomolecules, may be attributed to the greater number of biochemical conversion steps needed to produce more chemically complex molecules [10].

However, when we went into more details into this analysis, some specific features emerged. When considering only glycerophospholipids, the review analysis in humans showed that a higher heritability was associated with a lower number of carbon atoms [9]. In pigs, however, there was, generally, a very low correlation within the glycerophospholipid family between the number of carbon atoms and heritability, when this information was not dissected based on the number of double bonds (*r* = 0.16–0.25). When double bonds were considered, heritability in pigs increased for the molecules of this family that had a higher number of unsaturated positions (*r* = 0.42–0.50), similarly to what was also reported in humans [9]. Other trends in heritability were noted for some specific sub-groups of phosphatidylcholines. For example, within the phosphatidylcholine acyl-alkyl with 3 double bonds (PC ae CX:3; X is the number of carbon atoms) heritability decreased as the number of carbon atoms increased (-0.91 ≤ *r* ≤ -1.00). Additionally, within the phosphatidylcholines acyl-alkyls with 36 and 38 carbon atoms (PC ae C36:X and PC ae C38:X; X is the number of double bonds), heritability increased with an increasing number of double bonds (PC ae C36:X, 0.74 ≤ r ≤ 0.93; PC ae C38:X, 0.72 ≤ r ≤ 0.96) (Additional file 3, Figure S3). When focusing solely on phosphatidylcholines, similarities between humans and pigs were observed in relation to the level of unsaturation, as molecules with a higher number of double bonds had greater heritability. However, when examining more specific sub-groups of phosphatidylcholines in pigs, higher heritability was associated with a lower degree of unsaturation (Additional file 3, Figure S3).

From these results, we noted that there may not be a general relationship between the number of carbon atoms in glycerophospholipids. The analysis of the trends of heritability related to the complexity of the molecular structures of these metabolites should focus on more specific sub-groups.

**Text S3. Comparative information on candidate genes between humans and pigs**

For a total of 64 out of 97 mQTL that we identified in pigs (66%), analysis of the information retrieved in several databases and extensive literature mining (see Methods) made it possible to identify candidate genes affecting the metabolite levels in this livestock species, primarily by comparing results previously reported in humans. Among these mQTL, 41 had candidate genes that were already reported in humans to be associated with metabolites within the same family as those found in pigs. Additionally, 15 of these mQTL were associated with the same metabolites in human studies. The functions and roles of the candidate genes for the remaining 26 mQTL could be useful to further expand their influence on different metabolites, benefiting from the similarities between humans and pigs. Some differences in associated metabolites between humans and pigs may be attributed to differences in the metabolomic platforms used in humans, which could not detect all specific metabolites that the Biocrates platform can analyze. For 23 mQTL where no human studies indicated associations with any metabolites within the same analyte family as those shown to be associated in pigs, an extensive literature review, helped identify relevant candidate genes, as explained in Additional file 1, Table S11.

**Text S4. Identification and annotation of variants from whole genome resequencing data**

Mining whole genome resequencing data obtained from 88 Large White and 35 Duroc pigs of the same metabolized pig populations, and an additional 35 Landrace pigs led to the discovery of 1,420,757 single nucleotide variants (SNV), 105,364 deletions and 156,588 insertions, encompassing the 97 mQTL regions (±1 Mb from the top markers reported in Table 2). The distribution of the predicted consequences of these variants, based on VEP, is reported in Additional file 2, Table S13. Variants affecting the protein coding sequence are given in Additional file 1, Table S14. Considering the canonical form of transcripts, a total of 1,797 missense mutations were predicted by SIFT as deleterious. Another 596 disrupted the encoded proteins (frameshift variants, start lost and stop gained) or altered their sequence (161 in-frame insertions or deletions).

**Text S5. Evaluation of *KMO* mutations in relation to its protein sequence and structure**

Sequence analysis of the protein KMO_PIG (Q9MZS9) was performed using InterPro to identify protein domains [11]. The analysis showed the presence of a FAD-binding domain (a member of the Rossmann fold clan) in the region 9-324 (Additional file 3, Figure S13), as detected by the PFAM entry PF01494 (FAD_binding_3). The structure of the pig KMO has not yet been resolved. However, a structural model is available in SwissModel [12] and covers the portion 6-443 of the 471-residue long pig protein and the template is the structure of the rat KMO (PDB: 6LKD) resolved with X-ray diffraction at 3Å (Figure 5f). There is a sequence identity of 77.6% between the rat and the pig proteins. The seven variations that characterize the two main haplotypes are located along the entire sequence. Notably, three of these variations (S95F, Q135R, and V178L) are located in the FAD-binding domain and could potentially affect the cofactor binding and, therefore, the enzyme kinetics. The KMO protein works as dimer (chain A and chain B) [13] as depicted in Figure 5f.

**Text S6. Inclusion of the genetic effect in the Gaussian Graphical Model (GGM) construction**

The inclusion in the GGM of the information derived from the GWAS for the considered metabolites did not substantially change the observed network (Additional file 3, Figure S14). When considering the GGM for the Large White breed, including its specific mQTL, only 7 PCC increased in strength (ΔPCC>0.10). None of these were significant neither before nor after inclusion in the model the considered mQTL (Additional file 2, Table S17). Similarly, only other 7 PCC decreased their strength (ΔPCC<-0.10). These included only 2 PCC (SM C18:0–SM C18:1 and PC ae C36:5–PC ae C38:6) that were significant in the original GGM and still retained their significance after the inclusion of the genetic information (Additional file 2, Table S17). Only 8 PCC targeting a few biologically linked nodes (e.g. C2–C3; Spermine–Spermidine) increased in strength and surpassed the significance threshold, with a minimal gain in strength (Additional file 2, Table S17).

The impact of the genetic information was more pronounced in the network constructed using simple Pearson’s correlations (*r*; Additional file 3, Figure S14b). A total of 25 correlations had Δ*r*>0.1. Among the metabolites included in these correlations, several metabolites were recurrent, including PC aa C36:4 (in 8 correlations) and PC aa C36:1 (in 6 correlations). The pair PC aa C36:4–PC aa C36:1 had the highest Δ*r* (0.220), moving from 0.528 to 0.742 (not significant change evidenced in ΔPCC). Both metabolites have been associated with an mQTL that could be explained by the *SMPD3/LPCAT2* genes, with the genotype that had a large impact on the single metabolites (Additional file, Figure S15a). Scatterplot analysis showed that the pigs clustered separately based on their SMPD3/LPCAT2 genotypes (Additional file, Figure S15b) promoting a low correlation. Inclusion of the genetic factor removed the genotype effect (Additional file, Figure S15c) and eliminated the clustering (Additional file, Figure S15d), leading to a strong increase in correlation. A similar, but less pronounced effect was observed with the mQTL (*SLC6A4* gene) linked to serotonin and taurine (Additional file: Fig. S16), which increased the PCC from 0.593 to 0.612 and the simple Pearson’s correlation from 0.622 to 0.626. In this second example, the effect of the tag SNP does not modify the plot distribution of the pigs with different genotypes, probably due to the same direction of the effect (*β* of association) for both metabolites.

**Text S7. Merging GGM and GWAS results**

In the GGM, metabolites of the same family tended to be more interconnected to each other than with metabolites of other classes. Therefore, the GGM were further analyzed considering the mQTL affecting the single metabolite abundance or their ratios (as derived from GWAS), by evaluating the sub-networks related to sphingomyelins and lysophosphatidylcholines.

In the first example we evaluated the reconstruction of relationships among few metabolites belonging to the sphingomyelins class. The obtained network (Additional file 3, Figure S17a) was similar to the network obtained by Krumsiek et al. [14] with human data, and characterized by the high positive PCC between SM C16:1–SM C18:1 and SM C18:0–SM C18:1, and the negative PCC between SM C18:1 and SM C16:1 due to the mathematical properties of partial correlation coefficients. To fully evaluate the link between metabolite-metabolite and gene-metabolite relationships, we revisited to the network (Additional file 3, Figure S17b) based on simple Pearson’s correlation coefficients (as PCC could indirectly absorb the genetic effect). Additional file 3, Figure S17c and Figure 17d show the shared mQTL acting on the metabolites entering in the network. We noted that highly correlated metabolites (such as SM C18:0–SM C18:1), also presenting a high PCC, showed association only when used as single molecule in GWAS but did not show any association when they were used with the ratios. Again, the ratio could not capture any variability, further supporting the GGM reconstruction of the network pathway.

In the second example we evaluated the reconstruction of relationships among few metabolites belonging to the lysophosphatidylcholines class. Based on the GGM, only two metabolites (LysoPC a C16:1 and LysoPC a C18:1) were linked (PCC = 0.70). Thus, also in this case, we went back to the network (Additional file 3, Figure S18) based on simple Pearson’s correlation coefficients and studied the metabolite-metabolite and gene-metabolite relationships. Similarly to what was previously described, several metabolites and metabolite ratios resulted associated with the same mQTL (mQTL 41; *LPCAT2/SMPD3* genes) but high correlated metabolites, as their ratio could not capture any variability.

**References**

1. Hirai K, Kuroyanagi H, Tatebayashi Y, Hayashi Y, Hirabayashi-Takahashi K, Saito K, et al. Dual role of the carboxyl-terminal region of pig liver L-kynurenine 3-monooxygenase: mitochondrial-targeting signal and enzymatic activity. J Biochem. 2010;148:639–50.
2. Brawand D, Soumillon M, Necsulea A, Julien P, Csárdi G, Harrigan P, et al. The evolution of gene expression levels in mammalian organs. Nature. 2011;478:343–8.
3. Nygard AB, Jørgensen CB, Cirera S, Fredholm M. Selection of reference genes for gene expression studies in pig tissues using SYBR green qPCR. BMC Mol Biol. 2007;8:67.
4. Park SJ, Kwon SG, Hwang JH, Park DH, Kim TW, Kim CW. Selection of appropriate reference genes for RT-qPCR analysis in Berkshire, Duroc, Landrace, and Yorkshire pigs. Gene. 2015;558:152–8.
5. Bovo S, Di Luca A, Galimberti G, Dall’Olio S, Fontanesi L. A comparative analysis of label-free liquid chromatography-mass spectrometry liver proteomic profiles highlights metabolic differences between pig breeds. PLoS One. 2018;13:e0199649.
6. Schindelin J, Arganda-Carreras I, Frise E, Kaynig V, Longair M, Pietzsch T, et al. Fiji: an open-source platform for biological-image analysis. Nat Methods. 2012;9:676–82.
7. Byrne JC, Downes MR, O’Donoghue N, O’Keane C, O’Neill A, Fan Y, et al. 2D-DIGE as a strategy to identify serum markers for the progression of prostate cancer. J Proteome Res. 2009;8:942–57.
8. R Core Team, *R* Foundation for Statistical Computing 2023.
9. Hagenbeek FA, Pool R, van Dongen J, Draisma HHM, Jan Hottenga J, Willemsen G, et al. Heritability estimates for 361 blood metabolites across 40 genome-wide association studies. Nat Commun. 7 2020;11:39.
10. Draisma HHM, Beekman M, Pool R, van Ommen GJB, Adamski J, Prehn C, et al. Familial resemblance for serum metabolite concentrations. Twin Res Hum Genet. 2013;16:948–61.
11. Paysan-Lafosse T, Blum M, Chuguransky S, Grego T, Pinto BL, Salazar GA, et al. InterPro in 2022. Nucleic Acids Res. 6 2023;51:D418–27.
12. Waterhouse A, Bertoni M, Bienert S, Studer G, Tauriello G, Gumienny R, et al. SWISS-MODEL: homology modelling of protein structures and complexes. Nucleic Acids Res. 2018;46:W296–303.
13. Mimasu S, Yamagishi H, Kubo S, Kiyohara M, Matsuda T, Yahata T, et al. Full-length in meso structure and mechanism of rat kynurenine 3-monooxygenase inhibition. Commun Biol. 2021;4:159.
14. Krumsiek J, Suhre K, Illig T, Adamski J, Theis FJ. Gaussian graphical modeling reconstructs pathway reactions from high-throughput metabolomics data. BMC Syst Biol. 2011;5:21.
